# Constructing and decoding tree-ring-like surface patterns on self-assembled polymer platelets

**DOI:** 10.1038/s41467-026-76483-7

**Published:** 2026-08-20

**Authors:** Laihui Xiao, Tianlai Xia, Andrew P. Dove, Rachel K. O’Reilly

**Affiliations:** School of Chemistry, University of Birmingham, Birmingham, UK

**Keywords:** Polymers, Self-assembly, Polymer synthesis

## Abstract

Precise control of surface patterns in assembled nanostructures remains a significant challenge in materials science. Here, we introduce a scalable bottom-up strategy utilizing living crystallization-driven self-assembly (CDSA) to fabricate tunable 3D surface patterns on polymer platelets. Inspired by biological growth increments, our approach leverages temperature-regulated, time-dependent crystallization to direct nanoscale organization, mimicking how nature constructs layered architectures. By exploiting polymer systems with temperature-dependent crystallization kinetics, we achieved diverse morphologies—including layered, concave, and convex features through controlled co-assembly and kinetic self-sorting. Temporal-thermal modulation was implemented in a continuous flow reactor, where precisely programmed residence times and temperature profiles enabled spatially resolved material deposition and growth history encoded in tree-ring-like patterns - control that is challenging or impossible to achieve using conventional batch methods. These features achieved lateral and vertical resolutions of ~73 nm and ~2 nm, respectively, allowing quantitative determination of directional crystallization rates (22.8 nm/s along the long axis and 13.4 nm/s along the short axis). Furthermore, modulating the number of layers provided a practical means to tune surface wettability. Our bioinspired design framework bridges synthetic self-assembly and natural structural logic, expanding opportunities for programmable materials in nanotechnology and functional systems.

## Abstract (web summary)

Control of surface patterns in assembled nanostructures is desirable, but challenging to achieve. Here, the authors report the development of a flow-based crystallisation-driven self-assembly method for the preparation of polymer platelets with controlled 3D surface patterns.

## Introduction

Growth increments—periodic structural features formed through time-regulated deposition—are prevalent in nature, encoding environmental and physiological information^1^. For example, tree rings record terrestrial climate signals through cellulose growth patterns^2^, while coral density bands archive marine environmental changes via seasonal aragonite deposition rates^3^. These biological patterns inspire synthetic strategies for constructing temporally structured materials. Surface patterns of assembled nanostructure are also crucial for nanomaterial functionality, however achieving precise control remains challenging^4–6^. Conventional top-down methods, like photolithography and etching, can create three-dimensional (3D) patterns on 2D substrates with high precision but offer limited control over the third dimension^7^. Additionally, these techniques are often complex, equipment-intensive, and challenging to apply directly to assembled nanostructures.

Crystallization-driven self-assembly (CDSA) has emerged as a robust method for constructing well-defined anisotropic nanostructures^8–11^. Through this technique, polymers with crystalline blocks undergo directional crystallization, and the nanostructure size can be precisely tuned via a seeded growth process, known as living CDSA. Specifically, when preparing two-dimensional (2D) platelet structures, co-assembly of homopolymers and diblock copolymers is commonly employed to achieve complex, tunable architectures, with each polymer type contributing distinct structural and functional roles to the final assembly^12–14^. Importantly, varying the polymer composition and processing conditions impacts key structural features, such as morphology, stability, and size, allowing precise engineering of functional nanostructures^12,15–17^. Our previous work demonstrated a graft-from strategy to extend corona length in specific areas of CDSA platelets to engineer defined 3D patterns^18^. However, this approach involves laborious synthesis and post-modification.

Potentially, an alternative strategy is to form layered structures by leveraging crystallization kinetics to control spatial and temporal polymer crystallization during assembly. Homopolymers often crystallize faster than diblock copolymers^12^, and thus enable spatial separation during co-assembly and formation of layered structures. This self-sorting behavior, where different components organize into distinct domains, is reminiscent of biological self-organization and enables hierarchical structure formation^19–21^. External stimuli such as temperature, pH, or solvent composition can further modulate this process^22,23^. When regulated temporally, such self-sorting offers a synthetic analogy to the natural growth increments at the nanoscale.

Here, mixtures of poly(ε-caprolactone) (PCL)-based homopolymers and diblock copolymers were assembled via living CDSA to generate platelets with controlled surface morphology. Variations in polymer molar mass and chemistry result in distinct crystallization rates in response to temperature^24,25^, thereby enabling kinetically driven self-sorting and co-assembly during seeded growth. This process was precisely regulated in flow reactors, where precise control over temperature and residence time facilitated reproducible spatial organization of polymer within platelets, leading to the formation of platelets with well-defined 3D surface features. Notably, flow reactors are most often employed to translate optimized batch reactions into continuous mode for purposes such as scale-up, reproducibility, and safety, which limits their application in exploratory research. In contrast, our work demonstrates that flow can be directly leveraged to engineer complex nanostructures inaccessible in batch, by exploiting programmable thermal and temporal profiles. This strategy mimics nature’s ability to generate layered structures through time-dependent material deposition—an engineered analog of biological incremental growth. Through this bioinspired approach, we establish a route to program hierarchical surface morphology in assembled nanostructures.

## Results and discussion

**Fig. 1:**
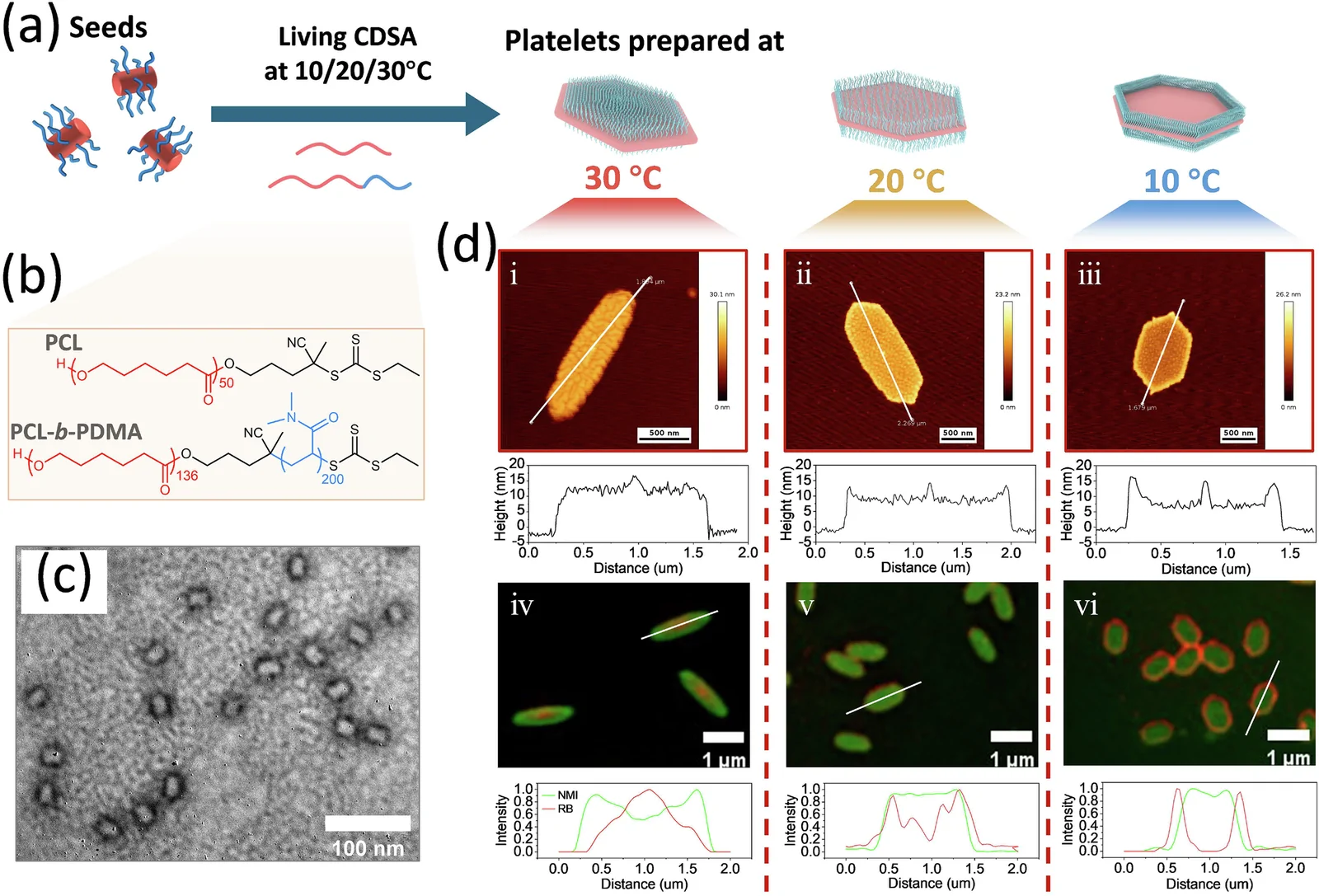
Batch living CDSA with 10 eqv. unimer (PCL_50_/PCL_136_-*b*-PDMA_200_, 1:1wt%) at various temperatures. **a** Schematic illustration for batch living CDSA, **b** Chemical structures of homopolymer (PCL_50_) and diblock copolymer (PCL_136_-*b*-PDMA_200_), **c** TEM image of seeds, and **d** Characterized platelets prepared at 30, 20, and 10 °C. The top row (ⅰ–ⅲ) show AFM images with corresponding height profiles. The bottom row (iv–vi) are SIM images of dye-labeled platelets prepared from naphthalene monoimide (NMI)-modified PCL_50_ (green) and rhodamine B (RB)-modified PCL_136_-*b*-PDMA_200_ (red), along with the corresponding normalized fluorescence intensity profiles. TEM image of seeds was stained by uranyl acetate aqueous solution (1%). Scale bar = 100 nm (**c**), 500 nm (**d**, ⅰ–ⅲ), and 1 µm (**d**, ⅳ–ⅵ).

Before conducting CDSA (Fig. 1a), polymeric building blocks were synthesized initially. PCL homopolymer (PCL_50_, the subscript refers to the number-average polymerization degree) and diblock copolymer (poly(ε-caprolactone)-*b*-poly(N,N-dimethylacrylamide), PCL_136_-*b*-PDMA_200_) were prepared by the combined ring opening and reversible addition-fragmentation chain-transfer (RAFT) polymerization (Fig. 1b and Fig. S1)^26–29^. The polymerization degree was confirmed by proton nuclear magnetic resonance (^1^H NMR) spectroscopy (Figs. S2 and S3), and the molar mass and dispersity were characterized using size exclusion chromatography (SEC, Fig. S4 and Table S1). In contrast to our previous work^12,14,18,26,29^, a diblock copolymer (PCL_136_-*b*-PDMA_200_) with a much longer PCL block was prepared to increase the crystallization capacity to a level comparable to that of the PCL_50_ homopolymer, thereby enabling external stimuli to modulate their relative crystallization rates.

Temperature is a readily tunable external stimulus that can significantly influence the crystallization behavior of polymers^24^. To explore the effect of temperature on particle formation, living CDSA was conducted over a range of temperatures from 10 to 30 °C, to control the dynamics of particle growth. The same seed sample previously reported (Fig. 1c and Fig. S5), with an average size of 25.2 nm, was used for this process^26^. Following the addition of unimer (PCL_50_/PCL_136_-*b*-PDMA_200_, 1:1 weight ratio, 10 mg mL^−1^ in CHCl_3_; Fig. 1a) to seed solution (0.01 mg mL^−1^ in ethanol), platelets with diverse morphologies were formed, as shown in the atomic force microscopy (AFM) images (Fig. 1d(ⅰ–ⅲ) and Fig. S6, and Table S2).

Previous results indicated that platelet patterns are formed by polymer coronas, representing the distribution of diblock copolymers^12,18^. Therefore, height profiles were recorded to map this distribution and characterize the 3D patterns. At 30 °C, the central region of the platelet (13.5 nm) was higher than the edge (11.1 nm), indicating substantial incorporation of diblock copolymers at the center due to the relatively small difference in growth kinetics between the two polymers at this temperature. When the temperature was reduced to 20 °C, the growth kinetics of the homopolymer became faster relative to those of the diblock copolymer, resulting in a decreased central height (9.9 nm) and increased accumulation of diblock copolymers at the platelet edges (12.8 nm), consistent with previous observations^12,18,30^. At 10 °C, the kinetic disparity increased further, forming distinct layered platelets with a thin central region having a height of 7.8 nm—corresponding to the PCL single crystal—and a thicker edge (15.5 nm) enriched in diblock copolymers^31^. To confirm that these temperature-dependent patterns were due to the increased crystallization tendency of the diblock copolymer, control samples were prepared using PCL_50_/PCL_50_-*b*-PDMA_200_ unimers (Fig. S7 and Table S3). In these controls, regardless of preparation temperature, the platelet edge consistently remained higher than the center.

Although AFM can map the distribution of diblock copolymers by detecting the height of the particle, differing in the presence or absence of coronal blocks, it cannot precisely determine the distribution of homopolymers. Therefore, fluorescence labeling was used as a complementary method to further investigate the distribution of polymers across platelets. Naphthalene monoimide (NMI, green) and rhodamine B (RB, red) were attached to the ends of homopolymers and diblock copolymers (Figs. S8–10)^32^, respectively, and the same procedure was followed for living CDSA. As shown in the structured illumination microscopy (SIM) images (Fig. 1d(ⅳ–ⅵ)), diblock copolymers exhibited the same temperature-dependent distribution as observed in AFM, concentrating at the platelet center at 30 °C and gradually extending toward the edges as temperature decreased. In contrast, PCL homopolymers were distributed throughout the platelets regardless of temperature and were observed in the same regions as the diblock copolymers for platelet samples prepared at 30 and 20 °C.

Next, we used Förster resonance energy transfer (FRET) to assess the degree of overlap between the two polymers on different platelet samples (Fig. S11a), as FRET occurs when the donor and acceptor are in close proximity, typically within 10 nm^32–35^. The fluorescent emission spectra were recorded upon excitation of the FRET donor at *λ* = 410 nm. To facilitate comparison, the spectra were normalized by the total fluorescence intensity at *λ* = 530 nm and 576 nm, corresponding to the maximum emission of the FRET donor and acceptor, respectively (Fig. S11b). The results showed significant FRET in all samples, indicated by a prominent emission peak at *λ* = 576 nm. As the temperature for platelet preparation increased, the decreasing intensity of donor and the increasing intensity of acceptor suggested enhanced FRET efficiency and greater overlap between the polymers^35^. These findings, coupled with the AFM results, indicate that self-sorting occurs at 10 °C, while co-crystallization takes place at 30 °C.

The different crystallization behaviors of homopolymers and diblock copolymers over the range of temperatures studied were explored to elucidate thermodynamic and kinetic aspects of the assembly formation. Solution-based crystallization enthalpy was measured by nano DSC, and used to determine crystallinity (Fig. S12 and Table S4)^36^. Interestingly, the crystallinity of the homopolymer (80%) and diblock copolymer (92%) were comparable across the solution-based crystallizations at all temperatures, and much higher than those obtained from their bulk state (homopolymer: 56%, and diblock copolymer: 42%, Fig. S13 and Table S4). We reasoned that the enhanced diffusion and chain mobility in the solution state, together with reduced chain entanglement compared to the bulk state, promote to form perfect polymer crystals. Thus, we focused on the seeded growth kinetics instead, which were characterized using UV-Vis spectroscopy^37,38^. The growth kinetics of the two polymers were tested separately to evaluate their temperature dependence (Fig. S14a, b). The homopolymer exhibited faster growth kinetics at lower temperatures due to increased supercooling, consistent with prior reports on crystallization in the bulk state^24,39^. In contrast, the diblock copolymer showed the opposite trend, with lower temperatures resulting in slower growth kinetics. Consequently, the difference in growth kinetics between the two polymers was relatively small at 30 °C, but became substantially larger at 10 °C (Fig. S14d–f), even though the diblock copolymer measurements at 10 °C were conducted at elevated concentration. These observations are consistent with the experimentally observed co-assembly and self-sorting behavior at 30 °C and 10 °C, respectively. Nevertheless, when a mixture of homopolymer and diblock copolymer was used as the unimer, all systems investigated between 10 and 30 °C completed the growth process within minutes (Fig. S14c). We attribute these differing kinetic behaviors to hydrogen bonding between the PDMA corona and ethanol, which acts as a drag on molecular motion^40,41^. This hindrance is reduced at higher temperatures, where hydrogen bonds are more readily disrupted.

Differences in crystallization kinetics at varying temperatures play a critical role in determining the aspect ratio of the resulting platelets (Fig. 1d and Table S2). PCL crystallizes along the (110) and (200) planes, and at elevated temperatures the overall crystallization rate is reduced, favoring growth along the (110) plane, which possesses lower surface energy^38^. This preferential growth leads to platelets with higher aspect ratios at 30 °C. In contrast, under cooling conditions (e.g., 10 °C), the overall crystallization rate increases significantly, providing sufficient driving force to overcome the energy barriers associated with both crystallographic planes more uniformly, resulting in platelets with lower aspect ratios.

Next, we systematically varied the homo-to-diblock ratio (0–100 wt%) and temperature to investigate their combined influence on platelet morphology, particularly in the vertical dimension (Figs. S15 and S16, and Table S5). AFM analysis quantified the height profiles at the platelet centers and edges across all ratios and temperatures (Fig. S17 and Table S6). Whereas previous reports suggested that the homo-to-diblock ratio strongly impacts morphology through concentration-dependent crystallization kinetics^13,42^; our results indicate that temperature is the dominant factor (Fig. S18). For example, at 10 °C, edge heights consistently increased while central heights remained constant at ~7.8 nm, indicating persistent self-sorting even at 80 wt% diblock content. Conversely, co-assembly was still observed when content of diblock copolymer was reduced to 20 wt%. Notably, varying unimer composition remains an effective strategy to fine-tune pattern height in the third dimension and achieve tailored structural features.

In order to leverage temperature-controlled self-assembly, we aimed to precisely modulate 3D platelet patterns through a simplified method. Previously, creating layered patterns on platelets required multiple unimer additions, which was time-consuming and produced low-resolution patterns^12,18^. Inspired by growth increment in nature forming periodic structural features like tree rings, we sought to precisely regulate the growth history of platelets, despite their epitaxial growth typically being complete within 2 min. We expected that if temperature-switching can be efficiently adjusted to regulate the polymer assembly behavior over short time periods, distinct layered patterns would form on the platelets, which would also show their growth history.

The proof-of-concept living CDSA process was conducted in batch that initiated at 30 °C and then cooled to 10 °C. This led to a 3-layer pattern on platelets (Fig. S19 and Table S7). The first layer formed through the co-assembly of homopolymer and diblock copolymer at 30 °C, followed by self-sorted layers at 10 °C to yield the second layer dominated by homopolymer and the third layer formed by diblock copolymer only, confirming the feasibility of this temperature-controlled approach for layered patterning.

**Fig. 2:**
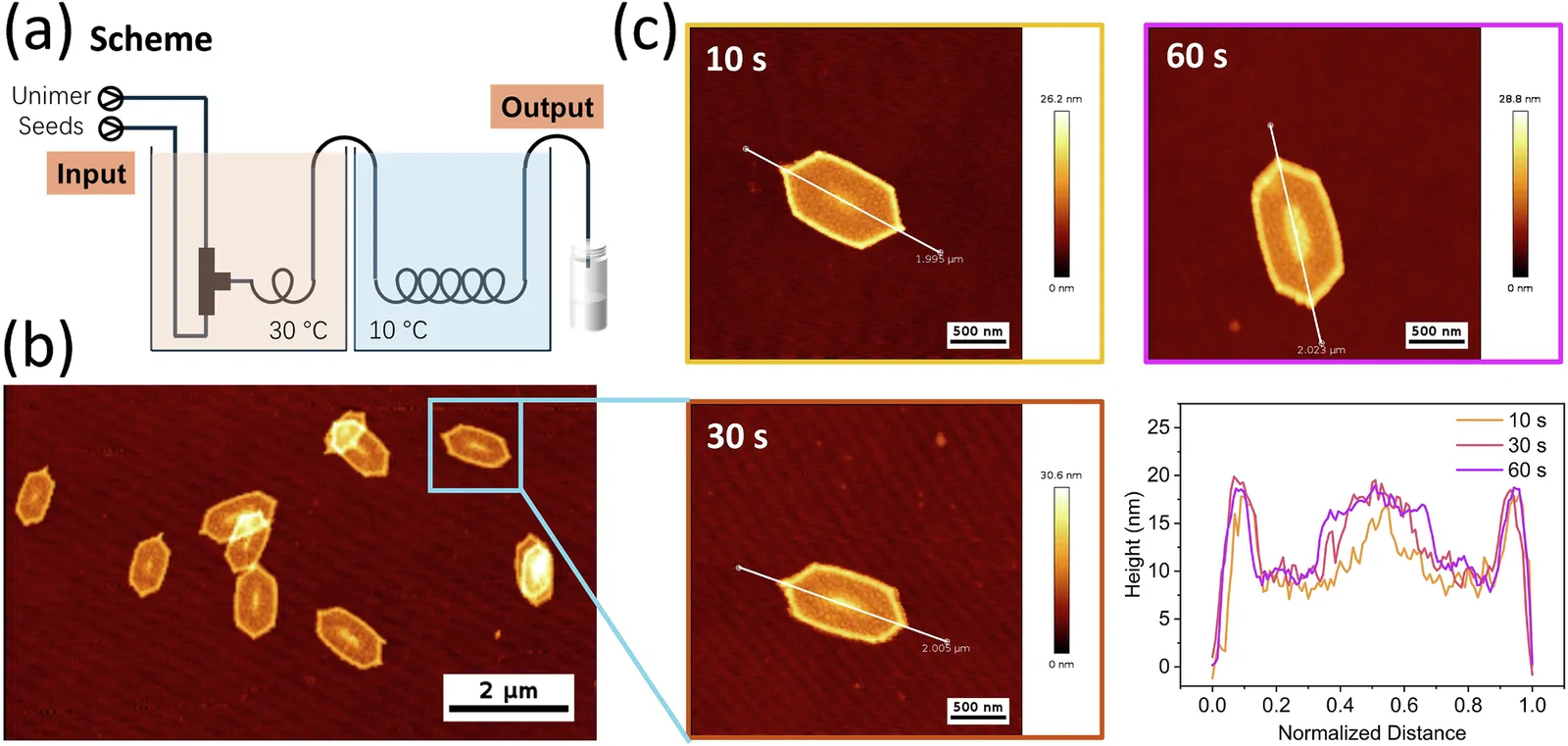
Discrete stage control of living CDSA via temperature switching in flow. **a** Schematic illustration of the flow setup with temperature-switching capability, **b** 3-Layer platelets prepared by sequential aging at 30 °C for 30 s, followed by 10 °C for 60 s, and **c** Tunable inner layer size achieved by varying the first-stage aging time at 30 °C (10, 30, and 60 s respectively), followed by aging at 10 °C. Scale bar = 2 μm (**b**) and 500 nm (**c**). Unimer (PCL_50_/PCL_136_-*b*-PDMA_200_, 1:1 wt%, 10 mg mL^−1^ in CHCl_3_) and seed solution (0.01 mg mL^−1^ in ethanol) were introduced at flow rates of 100 and 1 μL min^−1^, respectively.

To further refine the 3-layer structures, we addressed the challenge of achieving rapid temperature switching in batch processing, which led to non-uniform patterns and blurred interfaces between layers due to insufficient temperature control (Fig. S19). To overcome this limitation, we adapted the CDSA process into a tubular flow reactor with modular temperature-controlled channels (Fig. 2a and Table S8)^8,26,43^. As our previous results show no significant differences between living CDSA performed under batch and flow conditions^26^, and dilution does not markedly affect seed activity (Fig. S20)^14^, the flow setup and procedure used for living CDSA in this study was adapted from our earlier work. After the mixing of seed and unimer, epitaxial growth occurred as the combined stream passed through a channel with the temperature set to 30 °C in a water bath, before the flow proceeded into another water bath set at 10 °C. The high surface area-to-volume ratio of the flow reactor facilitated rapid heat transfer^44–46^, allowing precise temperature control that modulated the self-assembly behavior from co-assembly to self-sorting. Under these flow conditions, uniform three-layer platelets were consistently formed within 2 min (Fig. 2b and Table S9). Notably, a significant height variation (~9 nm) was observed between the layers, which we attribute to differences in corona distribution arising from the temperature-dependent self-assembly behavior. Although temperature can also influence the thickness of the PCL crystalline core, the variation observed is limited (~2 nm, Fig. S18e and Table S6), indicating that the observed height differences primarily arise from changes in block copolymer distribution rather than crystalline core thickness.

By varying the residence time at specific temperatures, we could program resulting patterns. To demonstrate this control, we programmed the flow to have different residence times at 30 °C, to regulate the domain size of the first layer. AFM images and height profiles clearly show that longer residence times lead to larger inner layers (Fig. 2c). Conversely, one can read the epitaxial growth history that co-assembly occurred initially at 30 °C followed by self-sorting at 10 °C, and also realize the difference between the residence time within samples.

**Fig. 3:**
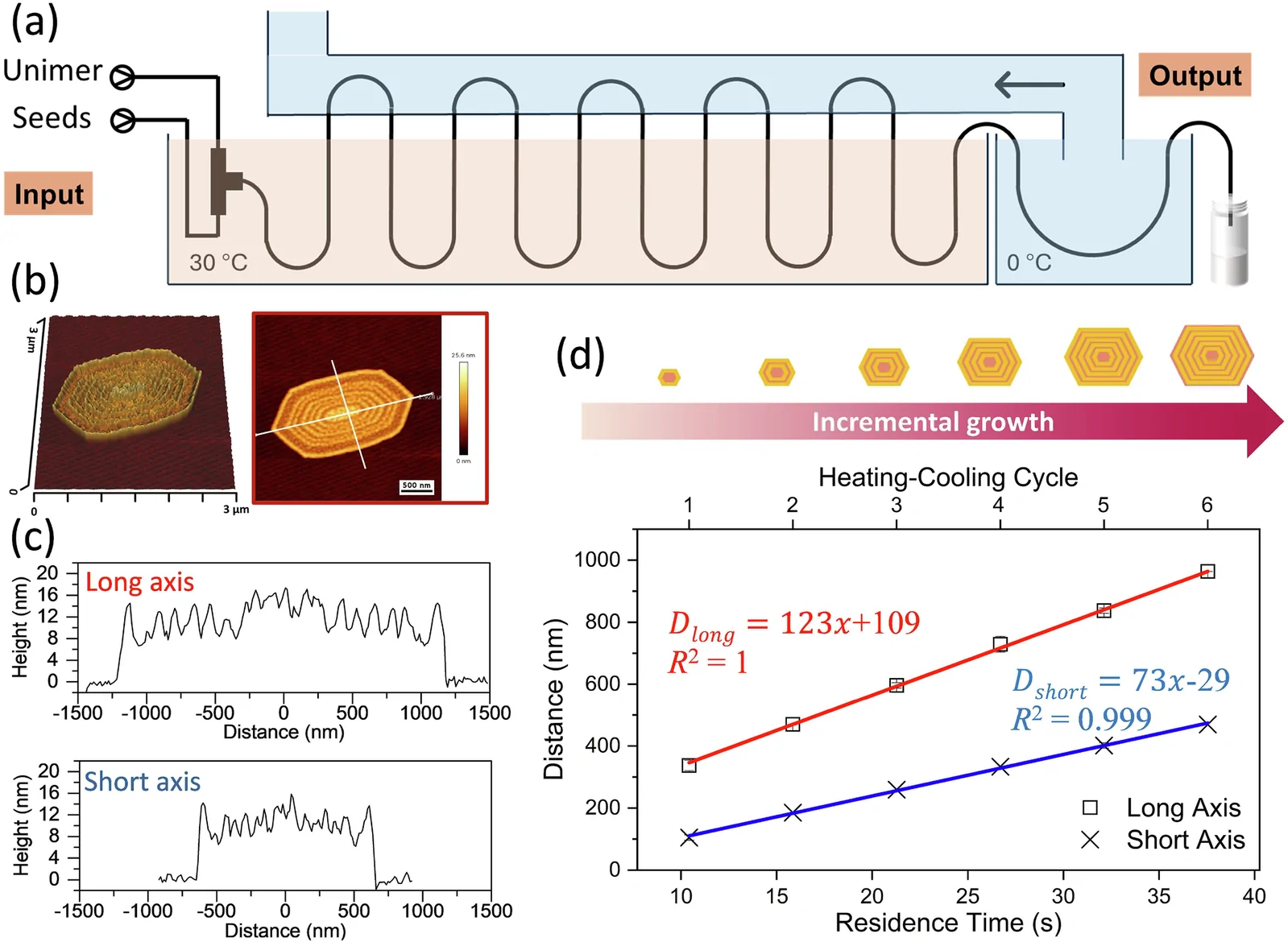
Cyclic temperature programming and growth kinetics in the formation of tree-ring-like multilayered platelets. **a** Schematic illustration of the flow setup with multistep temperature-switching capability, **b** AFM image of 13-layer platelets, shown as 3D rendering (left) and 2D height map (right), **c** Height profiles along the long (upper) and short (bottom) axes of platelets, and **d** Linear epitaxial growth of platelets. In the fitted equation, *D*_long_ and *D*_short_ represent the growth distance along the respective long and short axes, and *x* denotes the heating-cooling cycle. Data was collected at the troughs of height profiles. Scale bar = 500 nm. Unimer (PCL_50_/PCL_136_-*b*-PDMA_200_, 2:1 wt%, 5 mg mL^–1^ in CHCl_3_) and seed solution (0.0025 mg mL^–1^ in ethanol) were introduced at flow rates of 200 and 2 μL min^–1^, respectively. Further details of the flow conditions and concentrations are provided in the Supplementary Information.

Once the feasibility of temperature-switching for controlling epitaxial growth in living CDSA was established, we extended this strategy to incorporate multiple temperature transitions during growth, aiming to construct nanostructured platelets with tree-ring-like architectures. However, while conceptually simple, implementing rapid and accurate temperature switching poses a significant design challenge in continuous flow reactor development. In the conventional setup (Fig. 2a and Fig. S21a), tubing must pass through baths of differing temperatures, inevitably forming a transitional region with a thermal gradient. This gradual temperature change results in blurred interfaces between growth phases, producing platelets with indistinct or merged layers (Fig. S21b). To address this, we designed a dual-channel flow setup that sharply delineates temperature zones. A broad auxiliary tubing containing circulating cold water was suspended just above the surface of a heated water bath (Fig. 3a and Fig. S22). The reaction tubing—where epitaxial growth occurs—was guided through both channels, creating an abrupt thermal interface. Moreover, the cooling bath temperature was reduced to 0 °C to increase the thermal contrast and thereby enhance the structural distinction between the layers.

This optimized setup enabled the fabrication of multilayered platelets with distinct, alternating surface features reminiscent of natural tree rings, achieved in just 1 min at a flow rate of 200 µL min^–1^ (Table S10). As confirmed by AFM (Fig. 3b), a total of 13 clear layers were formed via 6 heating-cooling cycles (12 switching events). The height profiles along the platelet’s long and short axes (Fig. 3c) exhibited periodic undulations, reflecting alternating co-assembly and self-sorting phases. Initially, epitaxial co-assembly of homopolymer and diblock copolymer occurred at 30 °C. Upon switching to 0 °C, only partial crystallization of the homopolymer occurred, due to the short residence time (~0.43 s). This cycle repeated, gradually consuming unimers over six iterations. In the final cooling phase, the residual homopolymer and diblock copolymer self-sorted to form the 12th and 13th layers respectively—consistent with earlier observed mechanisms (Fig. 2).

Building on the successful formation of multilayered platelets, we next sought to extract growth kinetics. Analyzing the kinetics of platelet growth during living CDSA remains a significant challenge in the field, as noted in previous reports^14,47^. The fast assembly dynamics—often completed within minutes—combined with the nanoscopic scale and anisotropic growth, make it difficult to track and quantify growth in real time. In our earlier work, we used interferometric scattering (iSCAT) microscopy to monitor platelet growth on a substrate^47^. This approach required unusually large platelets to enhance optical contrast and relied on complex mathematical modeling to resolve nonlinear area expansion. Moreover, because the experiment was conducted on immobilized platelets, it does not fully reflect the growth behavior in solution, where self-assembly typically occurs. Prior studies have shown that growth kinetics can differ significantly between surface-bound and solution-phase systems^48^, underscoring the importance of conducting kinetic analyses under solution conditions.

Despite the overall growth completing within 1 min, the layered structure obtained in this study provided a distinct opportunity to retrospectively analyze the assembly kinetics. Much like dendrochronology in trees, each nanoscopic layer physically records a discrete growth increment. Height values at troughs in the height profiles, representing the conclusion of each heat-cooling cycle, were extracted and plotted against both cycle number and residence time (Fig. 3d and Table S11). Notably, these measurements revealed a linear increase in platelet length, indicating a constant axial growth rate. Using the cycle period (5.4 s), defined as the residence time for one complete heating-cooling cycle at a flow rate of 200 μL min^–1^, we calculated growth rates of 123 nm per cycle (22.8 nm/s) along the long axis and 73 nm per cycle (13.4 nm/s) along the short axis. These growth rates were highly consistent across multiple platelets, despite some variability in the dimensions of the first layer (Figs. S23 and S24, Tables S12 and S13). This variability may arise from differences in seed initiation efficiency, likely due to partial shielding of growth fronts by coronas, or from delayed homogenization of the seed and unimer streams under laminar flow conditions^49,50^. Such delayed initiation may also account for the negative y-axis intercepts observed in the linear fittings, as an induction period is required before unimers can efficiently access the active growth fronts. Once initiated, epitaxial growth proceeded at a uniform rate, independent of initial size. By segmenting the growth into short, discrete intervals, our approach enabled the resolution of subtle early-stage variations that would otherwise be obscured in continuous growth systems.

We further tuned the interlayer distance by varying the flow rate (Table S10). Theoretically, interlayer spacing is inversely correlated with flow rate, assuming the time-averaged growth rate per cycle remains constant (~22.8 nm/s along the long axis and ~13.4 nm/s along the short axis). As expected, decreasing the flow rate increased the interlayer spacing (Figs. S25–27, Tables S14–S15). Notably, although platelets prepared at lower flow rates (150 and 100 µL min⁻¹) did not exhibit all 13 layers observed at 200 µL min⁻¹, their average growth rates within each cycle (long axis: 22.8 and 24.0 nm/s; short axis: 12.2 and 13.1 nm/s) were consistent with the assumed values. These findings further demonstrate the flow reactor’s capability for precise nanoscale structural control.

Additionally, directional growth rates offer direct morphological insight into the anisotropic growth behavior of the platelets. Notably, the aspect ratio of the platelets decreased with increasing number of heating–cooling cycles (Fig. S28). As growth is initiated under heating conditions, the first layer exhibits a relatively high aspect ratio (3.21). With additional heating–cooling cycles, a larger fraction of the platelet area is formed under cooling conditions, resulting in a gradual reduction in the overall aspect ratio. This trend can be mathematically predicted that under conditions of sufficient unimer supply, increasing heating cooling cycles would drive the aspect ratio toward a limiting value of 1.79 (approximate to the theoretical value of 1.68, the ratio of growth rates along the long and short axes). Therefore, these findings provide rare, quantitative insight into directional crystallization kinetics in living CDSA and establish a robust method for resolving fast, anisotropic growth behavior through morphological analysis.

**Fig. 4:**
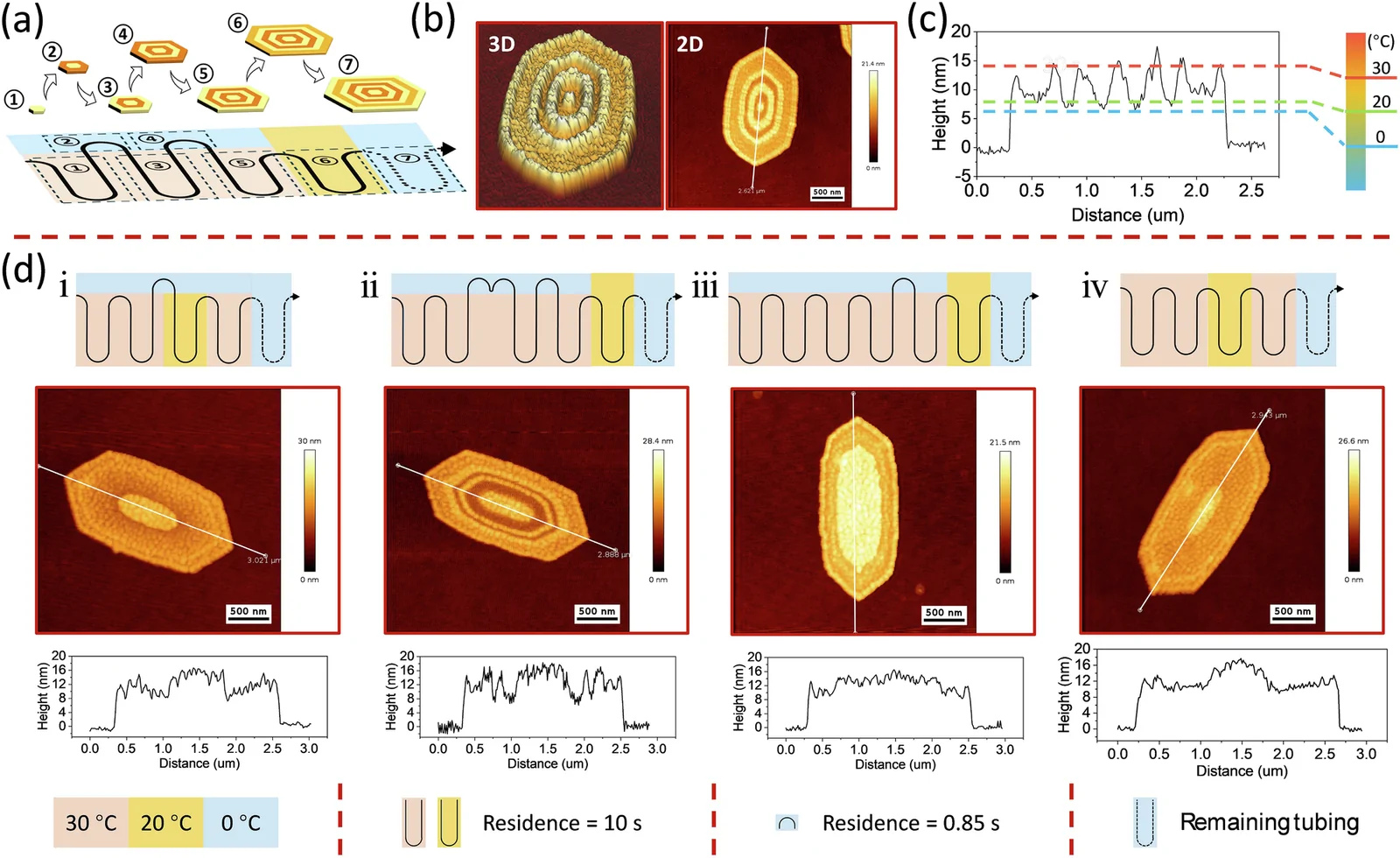
Encoding growth histories in platelets via programmable crystallization kinetics. **a** Schematic illustration of the flow setup used to fabricate multilayered platelets through controlled temperature and residence time programming, **b** AFM images of 7-layer platelets, shown as 3D rendering (left) and 2D height map (right), **c** Decoding growth histories from height profiles of multilayered platelets, and **d** Platelets prepared under various programmed temperature and residence time conditions. Top row: simplified illustrations of the flow setups; middle row: 2D AFM images; bottom row: corresponding height profiles. Scale bar = 500 nm. Unimer (PCL_50_/PCL_136_-*b*-PDMA_200_, 2:1 weight ratio, 5 mg mL^–1^ in CHCl_3_) and seed solution (0.0025 mg mL^–1^ in ethanol) were introduced at flow rates of 100 and 1 μL min^–1^, respectively.

It is well known that tree rings are not formed as uniform concentric circles; rather, each layer captures seasonal environmental variations. Inspired by this natural encoding, we sought to encode growth conditions into platelets by regulating temperature and residence time layer by layer, moving beyond the repetitive conditions by cyclic heating–cooling programs. As previous studies have shown that elevated temperatures facilitate mass diffusion and improve platelet uniformity^26^, all flow procedures were initiated at 30 °C, with a relatively larger proportion of the total residence time maintained at this temperature. To test this concept, we fabricated 7-layered platelets using a modified flow setup (Fig. 4a and Table S16). Epitaxial growth was initiated at 30 °C to drive co-assembly, followed by four cycles of temperature alternating between 0 °C and 30 °C to induce self-sorting and co-assembly, respectively. This sequence was then followed by a 20 °C channel to promote partial co-assembly, and a final 0 °C channel to induce self-sorting. Compared to samples prepared under purely cyclic conditions, these platelets exhibited a more pronounced hierarchical topography, as revealed by AFM images and the corresponding height profile (Fig. 4b, c).

We then attempted to decode the thermal history of platelet formation based on the observed height variations (Fig. 4c). Three distinct thickness regimes were identified: ~14.4 nm domains formed via co-assembly at 30 °C, ~10.2 nm regions resulting from partial co-assembly at 20 °C, and ~7.9 nm layers corresponding to homopolymer self-sorting at 0 °C—all consistent with control samples prepared in batch (Fig. S15). Although two distinct layers—one from homopolymer and one from diblock copolymer—were anticipated in the final cooling step, only a single outermost layer from the diblock copolymer was observed. This suggests that homopolymers were fully consumed during aging at 20 °C, and the residual diblock copolymers subsequently formed the terminal layer.

From these results, it is evident that temperature primarily determines vertical layer height, while residence time modulates lateral increment. To further explore the synergistic effects of temperature and residence time, we adjusted the flow program to generate platelets with varied surface architectures (Fig. 4d, Figs. S29 and S30, Tables S16 and S17). Once again, the height profiles enabled the reconstruction of the growth history. For instance, a gradual increase in thickness was observed when epitaxial growth proceeded sequentially at 0, 20, and 30 °C (Fig. 4d(ⅰ)), reflecting a progressive increase in diblock copolymer incorporation. This result underscores the effectiveness of programmed temperature modulation in guiding hierarchical assembly. Moreover, the ability to form multilayers within just 2 min enables rapid and efficient sequential information encoding, representing a notable advance in the design of CDSA-based information storage materials by combining high fabrication speed with enhanced pattern complexity^14,42,51^.

The key concept of this study is the temperature-dependent tuning of the relative crystallization rates of PCL-based homopolymer and block copolymer, coupled with flow processing to precisely control the thermal history, thereby enabling programmable nanoparticle morphologies during growth. In principle, this strategy can be extended to other CDSA systems comprising multiple components, provided that their self-assembly kinetics are sufficiently distinct yet responsive to external stimuli, allowing modulation of their relative rates. Such behavior may be achieved through polymer design or by tuning assembly conditions. More broadly, this flow-enabled approach offers a framework for achieving precise control over multicomponent self-assembly processes governed by nucleation-growth mechanisms.

Finally, in order to connect the nanoparticle structure with function, we prepared platelets with different numbers of layers (Figs. S31 and S32, Tables S18 and S19) and evaluated wettability as a representative interfacial property. Wettability is critical for nanoparticle interactions in aqueous and biological environments and was measured using quartz crystal microbalance (QCM), a sensitive technique for monitoring surface interactions under liquid^52^.

**Fig. 5:**
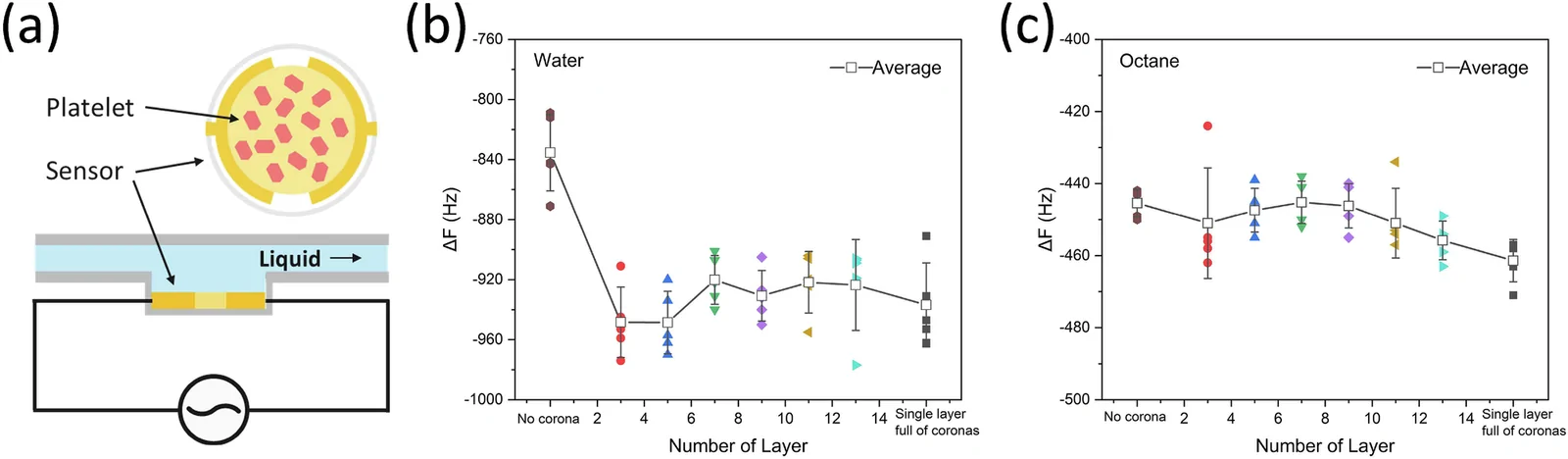
Platelet-liquid interactions studied by QCM. **a** Schematic illustration of the measurement setup, **b** Statistical resonance frequency changes upon transition from air to water, and **c** Statistical resonance frequency changes upon transition from air to octane. Data are presented as mean values ± standard deviation (*n* = 5).

Sensors were modified with different platelet samples and exposed to water flow (Fig. 5a). Upon transitioning from air to an aqueous environment, all modified sensors showed a decrease in resonance frequency (Fig. S33a and Table S20), reflecting the extent of water coupling at the surface of sensor and thus providing a relative measure of hydrophilicity^53^. The frequency shifts were statistically analyzed (Fig. 5b). When the sensor was modified with platelets lacking coronas, surface exhibited relatively lower hydrophilicity compared to corona-containing platelets, as reflected by a reduced QCM frequency shift response under identical conditions. Interestingly, among the corona-containing platelets, samples with a higher number of layers (7–13) exhibited lower apparent hydrophilicity than those with fewer layers (3–5). We attribute this behavior to an effect conceptually similar to the lotus effect, in which surface roughness combined with surface chemistry leads to incomplete wetting due to trapped air pockets^54,55^. In our system, although PDMA is intrinsically hydrophilic, the layered platelet architecture introduces nanoscale roughness that can partially suppress wetting by trapping air at the interface. As a result, increasing the number of layers diminishes the effective hydrophilic contribution of PDMA.

Because our platelets are amphiphilic particles containing both hydrophilic and hydrophobic domains, QCM measurements were also performed under a flow of octane to probe their affinity toward nonpolar solvents (Fig. S33b and Table S21). In this case, a larger decrease in resonance frequency indicates greater octane uptake and thus higher oleophilicity. Notably, the presence of a PDMA corona had little influence on the frequency shift compared to platelets without coronas, in contrast to the behavior observed under water (Fig. 5c). This result indicates that PDMA is not the dominant factor governing oleophilicity. Instead, variations in octane affinity are primarily associated with surface morphology, with an increasing number of layers leading to enhanced oil wetting. Together, these results demonstrate that nanoscale surface domain distribution and morphology play a key role in determining macroscopic wettability. This structure–property relationship highlights how flow reactors enable the fabrication of structurally precise nanoparticles with tunable interfacial properties, offering design principles for advanced applications in biomaterials, tissue engineering, and surface science.

In conclusion, we present a bioinspired polymer self-assembly strategy that couples temperature-responsive CDSA with precisely programmed flow reactors to encode growth history directly into nanoparticle morphology. By segmenting a tubular flow reactor into modular thermal zones with defined residence times, we establish flow reactors not only as scalable processing platforms but also as structure-encoding systems that enable incremental platelet growth on second timescales. This spatiotemporal control allows the fabrication of tree-ring-like surface architectures that are difficult to access using conventional batch methods. Analogous to dendrochronology, these layered surface patterns record both crystallization kinetics and environmental history, enabling direct extraction of directional growth rates and decoding of thermal and temporal assembly conditions from individual particles. Furthermore, we demonstrate that hierarchical surface patterning modulates platelet wettability solely through morphological variation, despite identical chemical composition, establishing a design principle that links nanoscale surface organization to macroscopic interfacial behavior. More broadly, this bioinspired strategy provides a scalable route to complex, programmable nanostructures, with implications for surface engineering, biomaterials, and functional nanotechnology.

## Methods

Detailed materials and instruments are given in the Supplementary Information.

### Synthesis of small molecules

2-Cyano-5-hydroxypentan-2-yl-ethyl carbonotrithioate (CHPET) and hydroxy-functionalized naphthalene monoimide (NMI-OH) were prepared through multistep syntheses. Detailed synthetic procedures and characterization data are provided in the Supplementary Information and Figs. S34–36.

### Polymer synthesis

#### Poly(ε-caprolactone) (PCL)

PCL_50_ and PCL_136_ were synthesized via ring-opening polymerization (ROP) in a nitrogen-filled glove box. Taking PCL_136_ as an example, caprolactone (7.33 g, 64.26 mmol), diphenylphosphate (DPP, 80.4 mg, 0.32 mmol), and CHPET (80 mg, 0.32 mmol) were accurately weighed in vials and then transferred to a 50-mL round-bottom flask. Toluene (22.54 mL) was used as the reaction solvent. Polymerization occurred at room temperature, with ^1^H NMR spectroscopy used for kinetic monitoring. After approximately 24 h, the reaction was quenched with Amberlyst, and the solution was removed from the glove box. The crude product underwent precipitation in cold diethyl ether three times, and the collected solid was obtained after centrifugation. A yield of 4.62 g of light-yellow solid was obtained and subsequently dried in the vacuum oven.

^1^H NMR (400 MHz, Chloroform-d, δ (ppm)): 4.12 (t, *J* = 6.2 Hz, 2H), 4.06 (t, *J* = 6.7 Hz, 272H), 3.35 (q, *J* = 7.4 Hz, 2H), 2.30 (t, *J* = 7.5 Hz, 272H), 1.88 (s, 3H), 1.71–1.59 (m, 544H), 1.44–1.31 (m, 272H).

^13^C NMR (101 MHz, Chloroform-d, δ (ppm)): 173.56, 77.35, 77.03, 76.71, 64.16, 34.13, 28.37, 25.55, 24.59.

SEC (Chloroform, PMMA standard): *M*_n_ = 31.0 kg mol^–1^, *Đ* = 1.06.

#### Poly(ε-caprolactone)-*b*-Poly(N,N-dimethylacrylamide) (PCL-*b*-PDMA) synthesized by RAFT polymerization

PCL_136_-*b*-PDMA_200_ was prepared by the chain extending using RAFT polymerization. As an example preparation for PCL_136_-*b*-PDMA_200_, macro-CTA (PCL_136_, 100 mg, 0.00634 mmol), DMA (150.8 mg, 1.52 mmol), and AIBN (0.104 mg, 0.000634 mmol, 10 mg mL^−1^ in dioxane) were dissolved in dioxane (0.76 mL) and then transferred into an ampoule. The solution was freeze-pump-thawed three times before the ampoule was immersed in an oil bath set at 70 °C. After 2 h, polymerization was quenched by immersing the ampoule in the liquid N_2_. The crude product was precipitated into the cold diethyl ether once it reached room temperature and then collected by centrifugation, and this process was repeated three times. After drying in a vacuum oven for 3 days, 215.5 mg of a solid product was obtained.

^1^H NMR (400 MHz, Chloroform-d, δ (ppm)): 4.06 (t, *J* = 6.7 Hz, 272H), 3.21–2.75 (m, 1200H), 2.30 (t, J = 7.5 Hz, 272H), 1.71–1.58 (m, 544H), 1.44–1.32 (m, 272H).

^13^C NMR (101 MHz, Chloroform-d, δ (ppm)): 174.45, 173.55, 64.15, 38.93–34.99 (m), 35.80, 35.67, 34.65, 34.12, 28.35, 25.53, 24.58.

SEC (Chloroform, PMMA standard): *M*_n_ = 43.8 kg mol^−1^, *Đ* = 1.18.

### Naphthalene monoimide-modified PCL (PCL-NMI)

PCL_53_-NMI prepared and used in this work were the same as we previously reported^8^. Specifically, NMI (78.2 mg, 0.24 mmol), caprolactone (1.93 g, 16.87 mmol), DPP (60.3 mg, 0.24 mmol), and toluene (16.9 mL) were charged into a 50-mL round-bottom flask. Polymerization was conducted at room temperature for approximately 10 h. Subsequently, the crude product was precipitated into the cold diethyl ether once it reached room temperature and then collected by centrifugation, and this process was repeated three times. Finally, 1 g of yellow solid was obtained after complete drying in a vacuum oven.

^1^H NMR (400 MHz, Chloroform-d, δ (ppm)): 4.06 (t, *J* = 6.7 Hz, 106H), 3.24 (t, *J* = 5.3 Hz, 4H), 2.30 (t, *J* = 7.5 Hz, 106H), 1.89 (t, *J* = 5.6 Hz, 4H), 1.72–1.59 (m, 212H), 1.43–1.33 (m, 106H).

^13^C NMR (101 MHz, Chloroform-d, δ (ppm)): 173.55, 77.35, 77.03, 76.71, 64.16, 34.13, 28.37, 25.55, 24.59.

SEC (Chloroform, PMMA standard): *M*_n_ = 13.6 kg mol^−1^, *Đ* = 1.07.

Fluorescence spectroscopy (Chloroform): maximum excitation at *λ* = 408 nm and maximum emission at *λ* = 537 nm.

### Red dye-modified PCL-*b*-PDMA (PCL-*b*-PDMA-RB)

Rhodamine B was grafted to PCL-*b*-PDMA by esterification reaction, which was catalyzed by 4-(dimethylamino)pyridine (DMAP) and N-ethyl-N'-(3-dimethylaminopropyl)carbodiimide hydrochloride (EDAC). In brief, PCL_136_-*b*-PDMA_200_ (100 mg, 0.00281 mmol), rhodamine B (6.73 mg, 0.014 mmol), EDAC (5.39 mg, 0.0281 mmol), DMAP (0.343 mg, 0.00281 mmol, 10 mg mL^–1^ in DCM), and DCM (2 mL) were added into a vial and stirred at room temperature for 2 days. After the reaction, the undissolved substance was removed by filtration, and the filtrate was precipitated into the cold diethyl ether and then the precipitated polymer was collected after centrifugation. Another five precipitation and centrifugation processes were conducted, and the final product was dried in a vacuum oven overnight. SEC equipped with a 540 nm UV detector was performed to confirm the successful attachment of rhodamine B to PCL-*b*-PDMA.

^1^H NMR (400 MHz, Chloroform-d, δ (ppm)): 4.06 (t, *J* = 6.7 Hz, 272H), 3.21–2.75 (m, 1200H), 2.30 (t, J = 7.5 Hz, 272H), 1.71–1.58 (m, 544H), 1.44–1.32 (m, 272H).

^13^C NMR (101 MHz, Chloroform-d, δ (ppm)): 173.56, 64.16, 37.30–36.94 (m), 35.96–35.47 (m), 34.85–34.65 (m), 34.13, 28.36, 25.54, 24.59.

SEC (Chloroform, PMMA standard): *M*_n_ = 44.7 kg mol^–1^, *Đ* = 1.12.

Fluorescence spectroscopy (Chloroform): maximum excitation at *λ* = 558 nm and maximum emission at *λ* = 580 nm.

### General procedure for crystallization-driven self-assembly (CDSA)

#### Seed preparation

Seeds prepared and used in this work were the same as we previously reported^26^. In this procedure, 10 mg of PCL_50_-*b*-PDMA_196_ was added to 2 mL of ethanol and heated at 75 °C for 3 h. After cooling to room temperature, the sample was aged for 7 days, resulting in long polydisperse cylindrical structures. Then the cylinder sample was diluted to 0.5 mg mL^–1^ and sonicated at 2 mL scale in a dry ice-acetone bath using cycles of 20 s sonication followed by a 100 s pause, for a total sonication time of 20 min. The size and distribution are shown in Fig. S5.

#### General procedure to prepare platelets by living CDSA

Living CDSA was performed in 2-mL vials. Briefly, seed solutions (1 mL, 0.01 mg mL^–1^ in ethanol) was equivalent for 2 min in water bath set at various temperatures (10, 20, and 30 °C), then unimer solution (10 µL, 10 mg mL^–1^ in CHCl_3_) was added. After being vigorously stirred for 10 s, solutions were aged at the same temperature for another 2 min. After that, samples were prepared for microscopy test.

#### Flow cascade to prepare three-layered platelet with single heating-cooling cycle

Seed solution (0.01 mg mL^–1^ in ethanol, 100 µL min^−1^) was injected into the flow cascade using a HPLC pump, and initially passed a pre-heating channel (ID = 0.75 mm, 1 min) immersed in 30 °C water bath. Then in a reverse T micromixer, seed flow mixed with unimer flow (10 mg mL^–1^ in CHCl_3_, 1 µL min^–1^) that injected by syringe pump, and the ratio of unimer-to-seed was constant at 10. After that, epitaxial growth occurred in an aging channel (ID = 0.3 mm) with various heating and cooling (immersed in 10 °C water bath) periods. Samples were collected after passing 3 reactor volumes to make sure they reached the steady state. The detailed flow setups for different experiments were listed in Table S8.

#### Flow cascade to prepare multi-layered platelet with several heating-cooling cycles

Detailed procedures for preparing layered platelets for different applications are provided in the Supplementary Information. In general, seed solution (0.0025 mg mL^–1^ in ethanol) was injected into the flow cascade using a HPLC pump, and initially passed a pre-heating channel (ID = 0.75 mm, 1 min) immersed in 30 °C water bath. Then in a reverse T micromixer, seed flow mixed with unimer flow (5 mg mL^–1^ in CHCl_3_) that injected by syringe pump, and the ratio of unimer-to-seed was constant at 20. After that, epitaxial growth occurred in an aging channel (ID = 0.3 mm) with various heating and cooling periods. Samples were collected after passing 3 reactor volumes to make sure they reached the steady state. The detailed flow setups for different experiments were listed in Tables S10, S18.

## Supplementary information

Supplementary Information

Transparent Peer Review file

## Data Availability

The data supporting the findings of this study are available within this paper and its Supplementary Information. Data may be requested from the corresponding authors.
