# Supplementary material for: Constructing and decoding tree-ring-like surface patterns on self-assembled polymer platelets
Source: Nat Commun. 2026 Aug 20;17:9961. doi: 10.1038/s41467-026-76483-7 (PMC13586395; doi:10.1038/s41467-026-76483-7)
Supplement: Supplementary file 1 [file 41467_2026_76483_MOESM1_ESM.pdf]

Supplementary Information

Constructing and Decoding Tree-Ring-Like Surface Patterns on Self-Assembled Polymer Platelets

Laihui Xiao,[a#] Tianlai Xia,[a#] Andrew P. Dove,[a]* Rachel K. O'Reilly[a]*

[a] School of Chemistry, University of Birmingham, Edgbaston, Birmingham B15 2TT, UK

*Corresponding Author: r.oreilly@bham.ac.uk (ROR) and a.dove@bham.ac.uk (APD)

[#]L.X. and T.X. contributed equally to this work.

Table of Contents

Materials S3

Instrumentation S3

Methods S5

Supplementary Figure S11

Supplementary Table S47

Supplementary References S64

Materials

The dual-functional chain transfer agent (CTA), 2-cyano-5-hydroxypentan-2-yl-ethyl carbonotrithioate (CHPET), was synthesized following our previous work.[1] Diphenyl phosphate (DPP, 99%, Sigma Aldrich) underwent drying with P_2O_5 and was stored in the glove box. ε-Caprolactone (99%, ACROS Organics) was dried with CaH_2, distilled, and then stored in a N_2-filled glovebox. The initiator, 2,2'-azobis(2-methylpropionitrile) (AIBN, 98%, Sigma Aldrich), was recrystallized in methanol and stored at 4 °C in the dark. 1,4-Dioxane (anhydrous, 99.8%, Fisher Scientific) and N,N-dimethyl acrylamide (DMA, 99%, Sigma Aldrich containing 500 ppm monomethyl ether hydroquinone as an inhibitor) were purified with basic alumina before each use. A hydroxy-functionalized naphthalene monoimide (NMI-OH) was synthesized following the literature.[2] Rhodamine B (≥95%, Sigma Aldrich), 4-(dimethylamino)pyridine (DMAP, Sigma Aldrich), N-Ethyl-N′-(3-dimethylaminopropyl)carbodiimide hydrochloride (EDAC, ≥98%, Sigma Aldrich), Sodium ethanethiolate (80%, Sigma Aldrich), carbon disulfide (⩾99.9%, Sigma Aldrich), solid iodine (⩾99.99%, Sigma Aldrich), 4,4'-azobis(4-cyanovaleric acid) (ACVA, ⩾98%, Sigma Aldrich), borane tetrahydrofuran complex solution (1.0 M in THF, Sigma Aldrich), 4-Bromo-1,8-naphthalic anhydride (95%, Sigma Aldrich), ethanolamine (≥98%, Sigma Aldrich), piperidine (99%, Sigma Aldrich), ethanol (anhydrous, ≥99.5%, Fisher Scientific), diethyl ether (anhydrous, ≥99.0%, Fisher Scientific), dichloromethane (DCM, anhydrous, ≥99%, Sigma Aldrich) and chloroform ($CHCl_3$, anhydrous, ≥99%, Sigma Aldrich) were used as received.

Instrumentation

Nuclear magnetic resonance (NMR) spectra were acquired using a Bruker DPX-400 (400 MHz) spectrometer, with deuterated chloroform ($CDCl_3$) serving as the solvent (residual $CHCl_3$ at 7.26 ppm). The data were analysed and processed using MestReNova x64 software. Size exclusion chromatography (SEC) was performed on Agilent 390-MDS equipment, employing PLgel Mixed-D type columns and detecting signals through refractive index (RI) and ultraviolet (UV) detectors. Chloroform with 0.5% NEt_3 was used as the eluent, and the flow rate was maintained at 1 $mL\cdot min^{-1}$. Number-average (M_n) and weight-average (M_w) molar masses, along with dispersity ($Đ$), were calibrated against polystyrene (PS) standards utilizing Agilent SEC software. UV-Vis spectroscopy was carried out on an Agilent Cary 6300 UV-Vis Spectrometer, with a wavelength range of 250 – 800 nm. The seed solution (2 mL) was equilibrated at the set temperature for 2 minutes prior to initiating measurements after unimer addition. Fluorescence spectra were recorded using an Edinburgh FS5 spectrofluorometer with a xenon light source and processed in

Fluoracle software provided with the instrument. Dynamic light scattering (DLS) was performed at 25 °C using a Malvern Zetasizer Nano ZS with a 4 mW He-Ne 633 nm laser module. Measurements were carried out at an angle of 173° (back scattering), and Malvern DTS v7.03 software was used to determine results. Differential scanning calorimetry (DSC) was conducted using a Mettler Toledo HP DSC827 instrument. Samples weighing 5-10 mg were subjected to heating and cooling cycles between -100 °C and 100 °C at 10 $°C·min^{-1}$ under a nitrogen atmosphere. Three cycles were executed for each sample, and data from the second cycle were analysed. Nano differential scanning calorimetry (Nano DSC) was conducted on a TA NanoDSC with 300 µL samples (1 mg mL^{-1}), using a heating and cooling rate of 1 °C min^{-1} under a constant pressure of 3 atmospheres. Atomic force microscopy (AFM) was performed on a JPK Nanowizard 4 system fitted with Nanosensor PPP-NCHAuD tips. All samples were tested in the supplied acoustic enclosure and vibration isolation at 20 °C, and the results were analysed by Data Processing Software V.6.1.117 software. Samples were prepared by spin-coating 10 µL of solution on a freshly cleaved mica wafer, the spin model was 3000 rpm for 50 seconds and 5000 rpm for 10 seconds. Platelet samples were statistically analyzed using ImageJ software according to our previously reported procedure.[1] The number-average length (L_n), weight-average length (L_w), number-average width (W_n), weight-average width (W_w), number-average area (A_n), weight-average area (A_w), and aspect ratio were determined and reported. Fluorescent samples were imaged by structured illumination microscopy (SIM) using COMPARE N-SIM S system coupled with 2x Hamamatsu Flash4.0 sCMOS camera. Image data were acquired by NIKON NIS Elements 5 software and analysed by NIS Elements 5 workstation, WXG92. Samples were prepared by spin-coating 10 µL of solution on cover glass, the spin model was 3000 rpm for 50 seconds and 5000 rpm for 10 seconds. The hydrophilicity of platelets was evaluated using a 5 MHz quartz crystal microbalance (QCM) system (openQCM Q-1, Novaetech S.r.l., Italy). Platelet samples (5 × 10 µL) were deposited onto QCM sensors and dried overnight. During measurements, water was introduced into the test chamber via a peristaltic pump (Masterflex C/L) at 0.5 mL min^{-1}, and the resonance frequency shift was recorded upon transition from air to aqueous / octane environment. Five replicates were performed for each sample.

Methods

Synthesis of Small Molecules

Dual-headed chain transfer agent (CTA)

2-Cyano-5-hydroxypentan-2-yl-ethyl carbonotrithioate (CHPET), used as both the initiator and chain-transfer agent (CTA) for polymerization, was prepared *via* a three-step synthesis (Figure S34).

Step-1: Carbon disulfide (7.74 mL) was added to a solution of sodium ethanethiolate (10 g) in diethyl ether (500 mL), which is immersed in an ice bath with a constant stir. After stirring for another 2 hours, excess iodine was added to the flask until the colour of the reaction system became dark brown. The solution was kept stirred overnight, and then it was washed with a solution of sodium thiosulfate (1M, 3 × 100 mL) followed by brine (3 × 100 mL) after it was transferred into a separation funnel. The solvent was removed by rotary evaporation after the organic layer was dried with anhydrous magnesium sulfate. 15.27 g (yield: 93.7%) of bis-(ethylsulfanylthiocarbonyl) disulfide was obtained.

^{1}H NMR (400 MHz, Chloroform-d, δ (ppm)): 3.32 (q, J = 7.5 Hz, 2H), 1.36 (t, J = 7.5 Hz).

Step-2: Bis-(ethylsulfanylthiocarbonyl) disulfide (9.0265 g, 32.88 mmol), ACVA (13.8245 g, 49.32 mmol), and ethyl acetate (500 mL) were charged into a 1-L round bottle flask equipped with a condenser, and the reaction was heated to reflux overnight in an N_2 atmosphere. After being concentrated by rotary evaporation, the crude product was purified through silica gel column chromatography (EtOAc / DCM = 1:3). 21.58 g (yield: 73.7%) of 4-cyano-4-[(ethylsulfanylthiocarbonyl)sulfanyl] pentanoic acid (CEPA) was obtained after thorough drying.

^{1}H NMR (400 MHz, Chloroform-d, δ (ppm)): 3.35 (q, J = 7.4 Hz, 2H), 2.73 – 2.62 (m, 2H), 2.62 – 2.48 (m, 1H), 2.40 (m, 1H), 1.89 (s, 3H), 1.36 (t, J = 7.4 Hz, 3H).

Step-3: CEPA (3 g, 11.4 mmol) was charged into a two-neck flask coupled with a dropping flask, and then the reaction apparatus was kept in the nitrogen flow and cooled to -78 °C with a dry ice – acetone bath. Dry tetrahydrofuran was used as the solvent and added to the flask with a syringe, and borane tetrahydrofuran (12 mL, 0.12 mmol) was dropped into the solution subsequently. After an hour of reaction at -78 °C, the solvent was left at room temperature and kept stirred overnight. 2-propanol was added to react with the extra borane. After the reaction, the solvent was removed by rotary evaporation, and diethyl ether (200 mL)

was added to dissolve the crude product. The solution was washed with saturated $NaHCO_3$ (3 × 200 mL) and then with brine (1 × 200 mL). The organic layer was concentrated by rotary evaporation after it was dried by anhydrous $MgSO_4$. Silica gel column chromatography (n-hexane / EtOAc = 1:1) was used to purify the product further, and 2.13 g (yield: 74.9%) of orange oil was finally obtained.

^{1}H NMR (400 MHz, Chloroform-d, δ (ppm)): 3.72 (t, J = 6.1 Hz, 1H), 3.35 (q, J = 7.4 Hz, 2H), 2.34 – 2.22 (m, 1H), 2.16 – 2.06 (m, 1H), 1.90 (s, 3H), 1.89 – 1.79 (m, 2H), 1.36 (t, J = 7.4 Hz, 3H).

^{13}C NMR (101 MHz, Chloroform-d, δ (ppm)): 217.39, 119.57, 61.80, 46.99, 35.76, 31.31, 27.91, 24.93, 12.80.

Synthesis of hydroxy-functionalized naphthalene monoimide (NMI-OH)

Hydroxy-functionalized naphthalene monoamide was prepared via a two-step synthesis (Figure S35).

Step-1: 4-Bromo-1,8-naphthalic anhydride (1.50 g, 5.41 mmol), ethanolamine (0.66 g, 10.81 mmol), and ethanol (35 mL) were charged to a round-bottom flask, and the mixture was refluxed at a set temperature of 80 °C for 24 h. After cooling down to room temperature, the resulting precipitate was collected by filtration, washed with saturated $NaHCO_3$ solution, and dried under vacuum to afford Br-NMI-OH (1.03 g, 60.6% yield).

^{1}H NMR (400 MHz, DMSO-d6, δ (ppm)): 8.63 – 8.50 (m, 2H), 8.38 – 8.16 (m, 2H), 8.00 (dd, J = 8.6, 7.3 Hz, 1H), 4.80 (t, J = 6.0 Hz, 1H), 4.14 (t, J = 6.5 Hz, 2H), 3.62 (q, J = 6.4 Hz, 2H).

Step-2: Br-NMI-OH (1.03g, 3.22 mmol) and excess piperidine (29.53 g, 346.8 mmol) were charged into a round-bottom flask, and the mixture was refluxed at 110 °C for 48 h. After cooling to room temperature, the mixture was dissolved in ethyl acetate (100 mL), washed with brine four times, dried over anhydrous magnesium sulfate, and concentrated by rotary evaporation. Finally, 0.65 g of yellow solid was obtained.

^{1}H NMR (400 MHz, Chloroform-d, δ (ppm)): 8.58 (dd, J = 7.3, 1.2 Hz, 1H), 8.50 (d, J = 8.2 Hz, 1H), 8.40 (dd, J = 8.5, 1.2 Hz, 1H), 7.68 (dd, J = 8.4, 7.3 Hz, 1H), 7.18 (d, J = 8.1 Hz, 1H), 4.49 – 4.42 (m, 2H), 3.97 (dd, J = 5.7, 4.7 Hz, 2H), 3.28 – 3.21 (m, 4H), 1.89 (p, J = 5.8 Hz, 4H), 1.73 (p, J = 5.9 Hz, 2H).

^{13}C NMR (101 MHz, Chloroform-d, δ (ppm)): 165.58, 165.15, 157.71, 133.14, 131.41, 131.06, 130.10, 126.23, 125.39, 122.82, 115.43, 114.75, 62.29, 54.56, 42.78, 26.22, 24.34.

Polymer Synthesis

Poly(ε-caprolactone) (PCL)

PCL_{50} and PCL_{136} were synthesized via ring-opening polymerization (ROP) in a nitrogen-filled glove box (Figure S1). Taking PCL_{136} as an example, caprolactone (7.33g, 64.26 mmol), diphenylphosphate (DPP, 80.4 mg, 0.32 mmol), and CHPET (80 mg, 0.32 mmol) were accurately weighed in vials and then transferred to a 50-mL round-bottom flask. Toluene (22.54 mL) was used as the reaction solvent. Polymerization occurred at room temperature, with ^{1}H NMR spectroscopy used for real-time monitoring. After approximately 24 h, the reaction was quenched with Amberlyst, and the solution was removed from the glove box. The crude product underwent precipitation in cold diethyl ether three times, and the collected solid was obtained after centrifugation. A yield of 4.62 g of light-yellow solid was obtained and subsequently dried in the vacuum oven.

^{1}H NMR (400 MHz, Chloroform-d, δ (ppm)): 4.12 (t, J = 6.2 Hz, 2H), 4.06 (t, J = 6.7 Hz, 272H), 3.35 (q, J = 7.4 Hz, 2H), 2.30 (t, J = 7.5 Hz, 272H), 1.88 (s, 3H), 1.71 – 1.59 (m, 544H), 1.44 – 1.31 (m, 272H).

^{13}C NMR (101 MHz, Chloroform-d, δ (ppm)): 173.56, 77.35, 77.03, 76.71, 64.16, 34.13, 28.37, 25.55, 24.59.

SEC (Chloroform, PMMA standard): M_n = 31.0 kg mol^{-1}, $Đ$ = 1.06.

Poly(ε-caprolactone)-*b*-Poly(N,N-Dimethylacrylamide) (PCL-*b*-PDMA) Synthesized by RAFT Polymerization

PCL_{136}-*b*-$PDMA_{200}$ was prepared by the chain extending using RAFT polymerization (Figure S1). As an example preparation for PCL_{136}-*b*-$PDMA_{200}$, macro-CTA (PCL_{136}, 100 mg, 0.00634 mmol), DMA (150.8 mg, 1.52 mmol), and AIBN (0.104 mg, 0.000634 mmol, 10 mg mL^{-1} in dioxane) were dissolved in dioxane (0.76 mL) and then transferred into an ampoule. The solution was freeze-pump-thawed three times before the ampoule was immersed in an oil bath set at 70 °C. After 2 h, polymerization was quenched by immersing the ampoule in the liquid N_2. The crude product was precipitated into the cold diethyl ether once it reached room temperature and then collected by centrifugation, and this process was repeated three times. After drying in a vacuum oven for 3 days, 215.5 mg of a solid product was obtained.

^{1}H NMR (400 MHz, Chloroform-d, δ (ppm)): 4.06 (t, J = 6.7 Hz, 272H), 3.21 – 2.75 (m, 1200H), 2.30 (t, J = 7.5 Hz, 272H), 1.71 – 1.58 (m, 544H), 1.44 – 1.32 (m, 272H).

^{13}C NMR (101 MHz, Chloroform-d, δ (ppm)): 174.45, 173.55, 64.15, 38.93 – 34.99 (m), 35.80, 35.67, 34.65, 34.12, 28.35, 25.53, 24.58.

SEC (Chloroform, PMMA standard): M_n = 43.8 kg mol^{-1}, $Đ$ = 1.18.

Naphthalene Monoimide-Modified PCL (PCL-NMI)

PCL_{53}-NMI prepared and used in this work were the same as we previously reported.[3] Specifically, NMI (78.2 mg, 0.24 mmol), caprolactone (1.93 g, 16.87 mmol), DPP (60.3 mg, 0.24 mmol), and toluene (16.9 mL) were charged into a 50-mL round-bottom flask. Polymerization was conducted at room temperature for approximately 10 h. Subsequently, the crude product was precipitated into the cold diethyl ether and then collected by centrifugation, and this process was repeated three times. Finally, 1 g of yellow solid was obtained after complete drying in a vacuum oven.

^{1}H NMR (400 MHz, Chloroform-d, δ (ppm)): 4.06 (t, J = 6.7 Hz, 106H), 3.24 (t, J = 5.3 Hz, 4H), 2.30 (t, J = 7.5 Hz, 106H), 1.89 (t, J = 5.6 Hz, 4H), 1.72 – 1.59 (m, 212H), 1.43 – 1.33 (m, 106H).

^{13}C NMR (101 MHz, Chloroform-d, δ (ppm)): 173.55, 77.35, 77.03, 76.71, 64.16, 34.13, 28.37, 25.55, 24.59.

SEC (Chloroform, PMMA standard): M_n = 13.6 kg mol^{-1}, $Đ$ = 1.07.

Fluorescence spectroscopy (Chloroform): maximum excitation at λ = 408 nm and maximum emission at λ = 537 nm.

Red Dye-Modified PCL-*b*-PDMA (PCL-*b*-PDMA-RB)

Rhodamine B was grafted to PCL-*b*-PDMA by esterification reaction, which was catalyzed by DMAP and EDAC. In brief, PCL_{136}-*b*-$PDMA_{200}$ (100 mg, 0.00281 mmol), rhodamine B (6.73 mg, 0.014 mmol), EDAC (5.39 mg, 0.0281 mmol), DMAP (0.343 mg, 0.00281 mmol, 10 mg mL^{-1} in DCM), and DCM (2 mL) were added into a vial and stirred at room temperature for 2 days. After the reaction, the undissolved substance was removed by filtration, and the filtrate was precipitated into the cold diethyl ether and then the precipitated polymer was collected after centrifugation. Another five precipitation and centrifugation processes were conducted, and the final product was dried in a vacuum oven overnight. SEC equipped with a 540 nm UV detector was performed to confirm the successful attachment of rhodamine B to PCL-*b*-PDMA.

^{1}H NMR (400 MHz, Chloroform-d, δ (ppm)): 4.06 (t, J = 6.7 Hz, 272H), 3.21 – 2.75 (m, 1200H), 2.30 (t, J = 7.5 Hz, 272H), 1.71 – 1.58 (m, 544H), 1.44 – 1.32 (m, 272H).

^{13}C NMR (101 MHz, Chloroform-d, δ (ppm)): 173.56, 64.16, 37.30 – 36.94 (m), 35.96 – 35.47 (m), 34.85 – 34.65 (m), 34.13, 28.36, 25.54, 24.59.

SEC (Chloroform, PMMA standard): M_n = 44.7 kg mol^{-1}, $Đ$ = 1.12.

Fluorescence spectroscopy (Chloroform): maximum excitation at λ = 558 nm and maximum emission at λ = 580 nm.

General Procedure for Crystallization-Driven Self-Assembly (CDSA)

Seed Preparation

Seeds prepared and used in this work were the same as we previously reported.[1] In this procedure, 10 mg of PCL_{50}-*b*-$PDMA_{196}$ was added to 2 mL of ethanol and heated at 75 °C for 3 h. After cooling to room temperature, the sample was aged for 7 days, resulting in long polydisperse cylindrical structures. Then the cylinder sample was diluted to 0.5 mg mL^{-1} and sonicated at 2 mL scale in a dry ice-acetone bath using cycles of 20 s sonication followed by a 100 s pause, for a total sonication time of 20 min. The size and distribution are shown in Figure S5.

General Procedure to Prepare Platelets by Living CDSA

Living CDSA was performed in 2-mL vials. Briefly, seed solutions (1 mL, 0.01 mg mL^{-1} in ethanol) was equivalent for 2 minutes in water bath set at various temperatures (10, 20, and 30 °C), then unimer solution (10 µL, 10 mg mL^{-1} in $CHCl_3$) was added. After being vigorously stirred for 10 seconds, solutions were aged at the same temperature for another 2 minutes. After that, samples were prepared for microscopy test.

Flow Cascade to Prepare Three-layered Platelet with Single Heating-Cooling Cycle

Seed solution (0.01 mg mL^{-1} in ethanol, 100 µL min^{-1}) was injected into the flow cascade using a HPLC pump, and initially passed a pre-heating channel (ID = 0.75 mm, 1 min) immersed in 30 °C water bath. Then in a reverse T micromixer, seed flow mixed with unimer flow (10 mg mL^{-1} in $CHCl_3$, 1 µL min^{-1}) that injected by syringe pump, and the ratio of unimer-to-seed was constant at 10. After that, epitaxial growth occurred in an aging channel (ID = 0.3 mm) with various heating and cooling (immersed in 10 °C water bath) periods. Samples were collected after passing through 3 reactor volumes to make sure they reached the steady state. The detailed flow setups for different experiments were listed in Table S8.

Flow Cascade to Prepare Multi-Layered Platelet with Several Heating-Cooling Cycles

To prepare platelets with multi-layered structures using the same heating–cooling cycle duration, different flow rates were studied (Table S10). Using 9-layered platelets as an example, seed solution (0.0025 mg mL^{-1} in ethanol, 100 μL min^{-1}) was injected into the flow cascade using a HPLC pump, and initially passed a pre-heating channel (ID = 0.75 mm, 1 min) immersed in 30 °C water bath. Then in a reverse T micromixer, seed flow mixed with unimer flow (5 mg mL^{-1} in $CHCl_3$, 1 μL min^{-1}) that injected by syringe pump, and the ratio of unimer-to-seed was constant at 20. After that, epitaxial growth occurred in an aging channel (ID = 0.3 mm) with 6 heating–cooling cycles. Samples were collected after passing through 3 reactor volumes to make sure they reached the steady state.

To prepare platelets with different numbers of layers for QCM measurements, the number of heating–cooling cycles was adjusted accordingly (Table S18).

Flow Cascade to Encode Growth Histories in Multi-Layered Platelets

Similar to the preparation of platelets with multi-layered structures described above, flow pathways with different temperature and residence time distributions were adjusted (Table S16). Seed solution (0.0025 mg mL^{-1} in ethanol, 100 μL min^{-1}) was injected into the flow cascade using a HPLC pump, and initially passed a pre-heating channel (ID = 0.75 mm, 1 min) immersed in 30 °C water bath. Then in a reverse T micromixer, seed flow mixed with unimer flow (5 mg mL^{-1} in $CHCl_3$, 1 μL min^{-1}) that injected by syringe pump, and the ratio of unimer-to-seed was constant at 20. After that, epitaxial growth occurred in an aging channel (ID = 0.3 mm) with various heating and cooling periods. Samples were collected after passing through 3 reactor volumes to make sure they reached the steady state.

Supplementary Figure

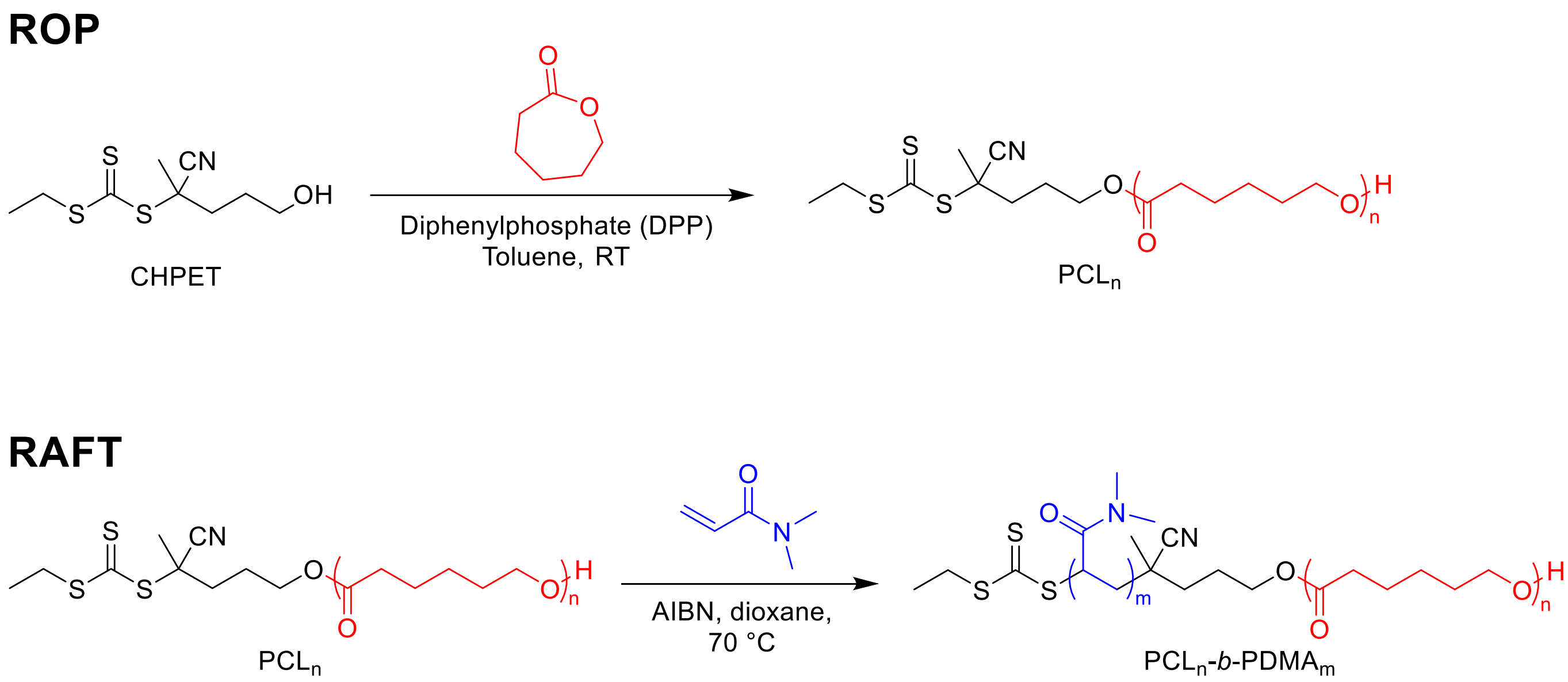

Figure S1. Synthetic route of PCL and PCL-*b*-PDMA.

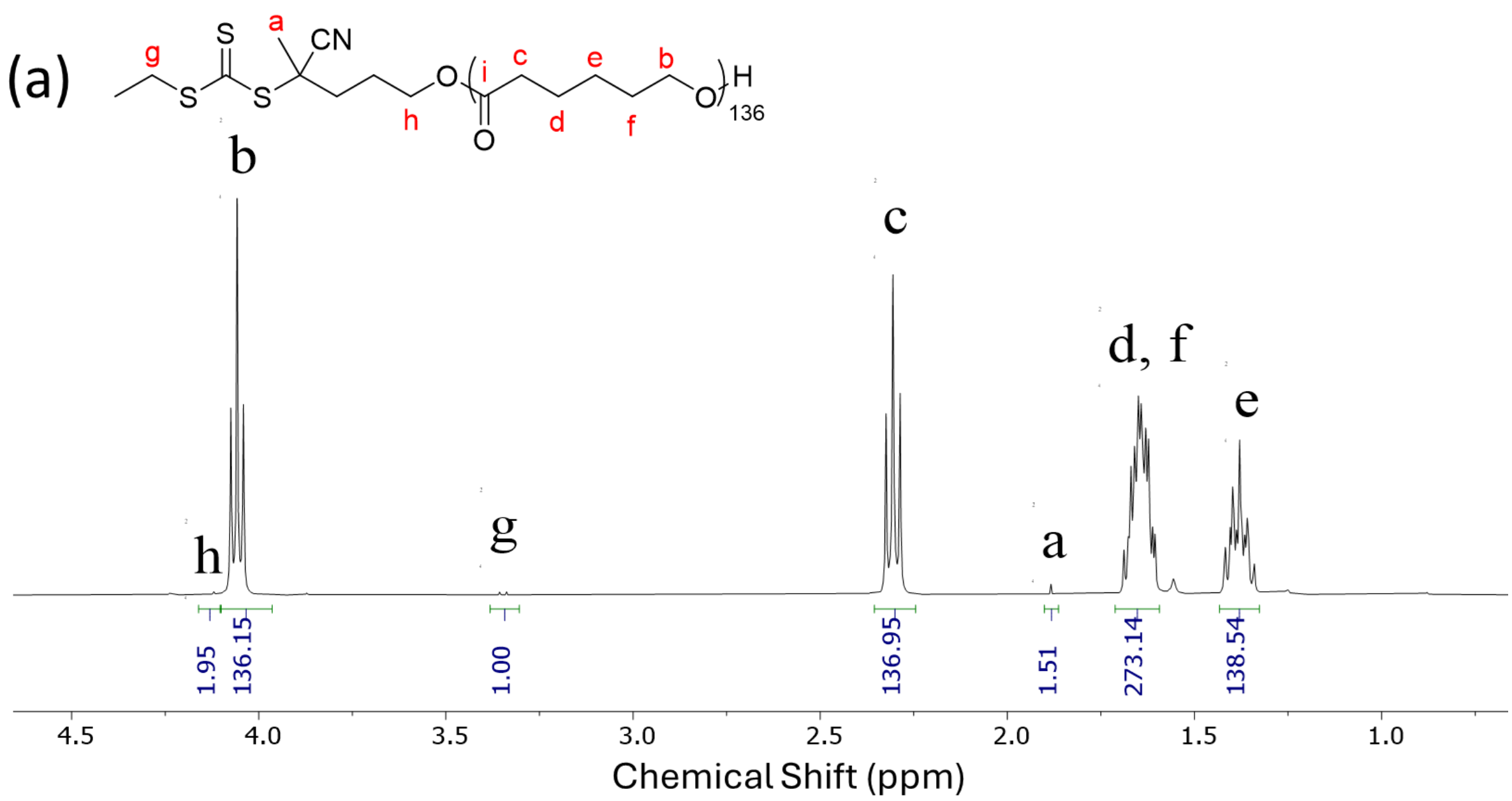

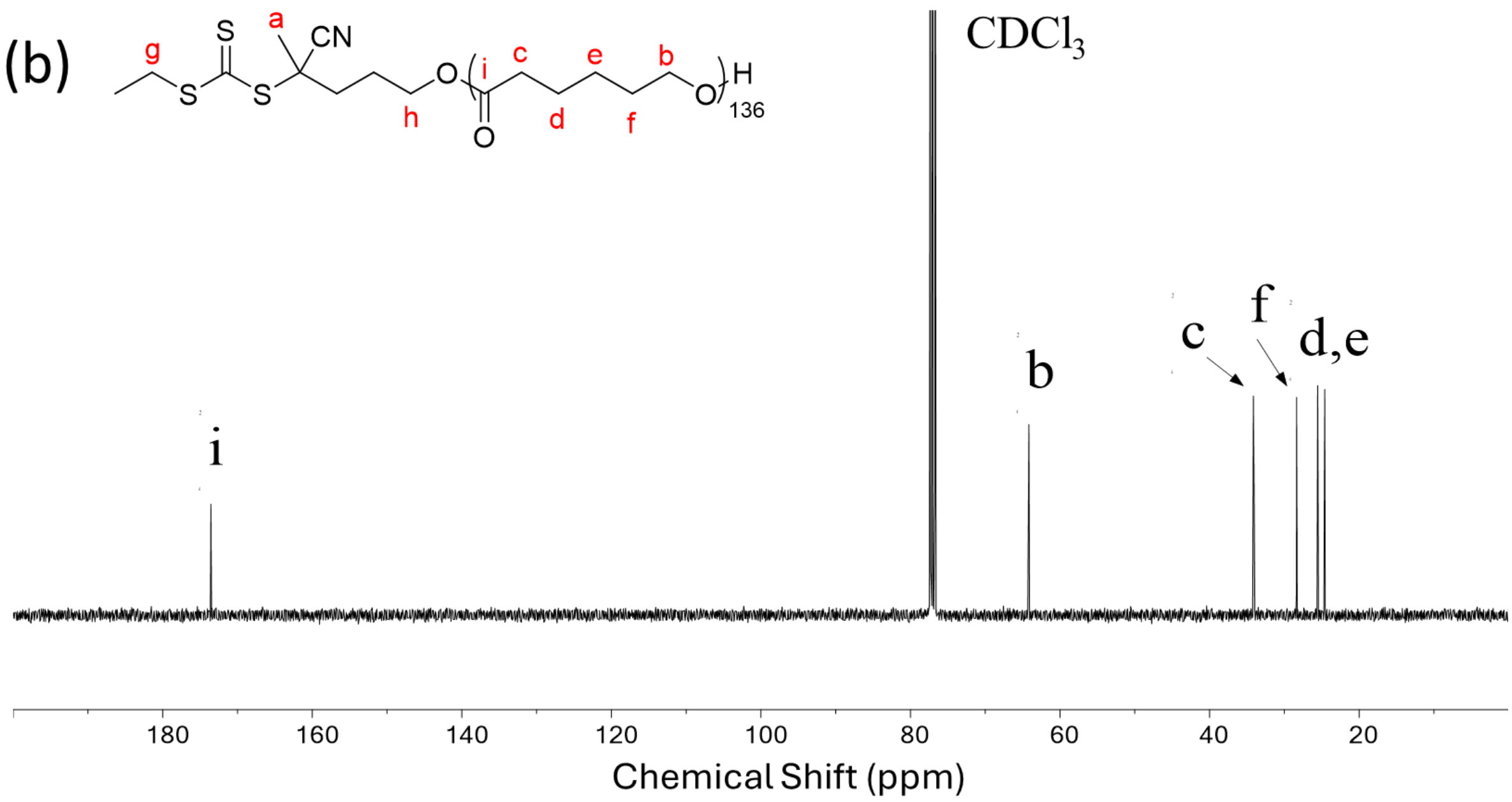

Figure S2. ^{1}H (a) and ^{13}C (b) NMR spectra of PCL_{136} (400 MHz; 298 K; in $CDCl_3$).

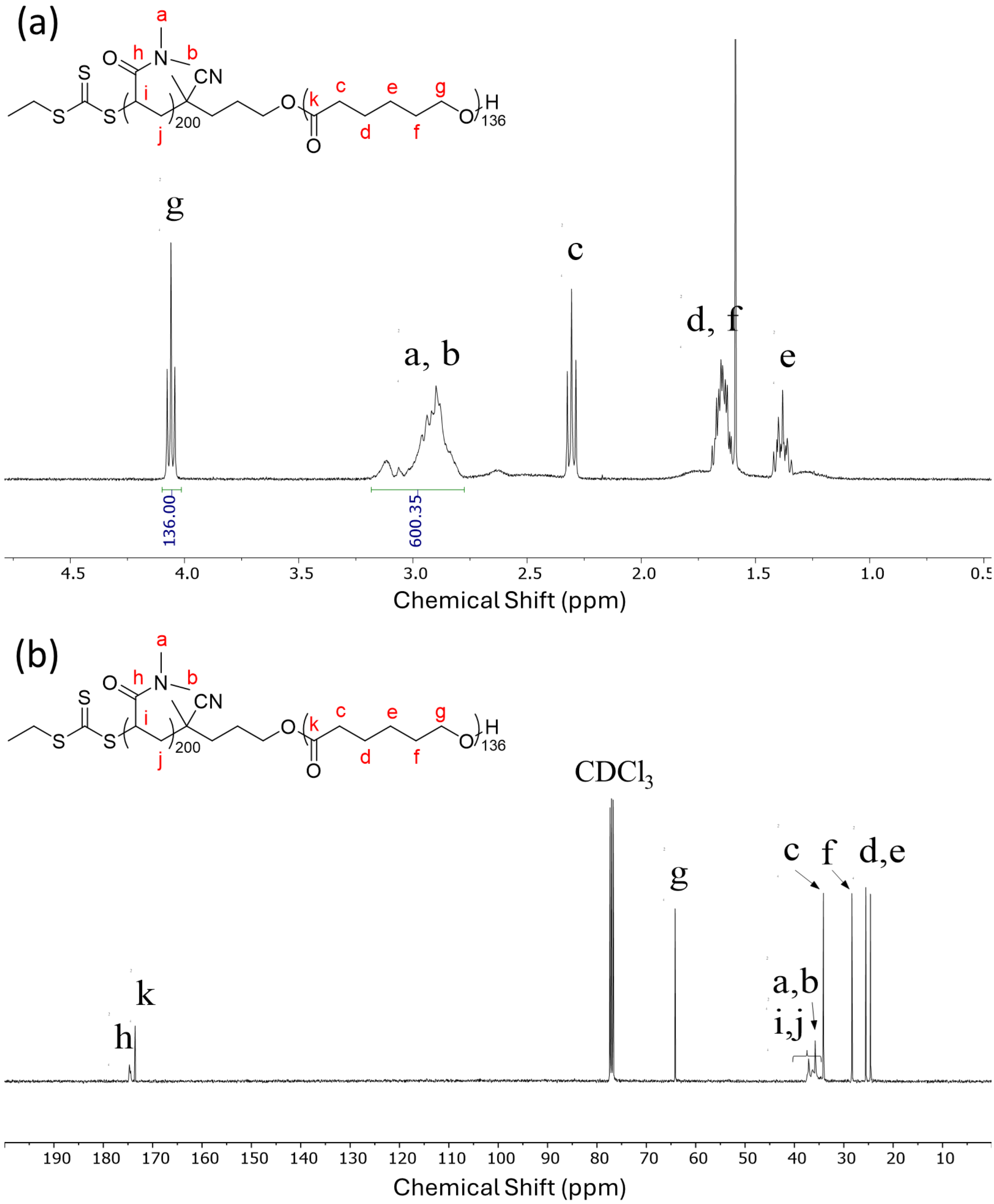

Figure S3. ^{1}H (a) and ^{13}C (b) NMR spectra of PCL_{136}-*b*-$PDMA_{200}$ (400 MHz; 298 K; in $CDCl_3$).

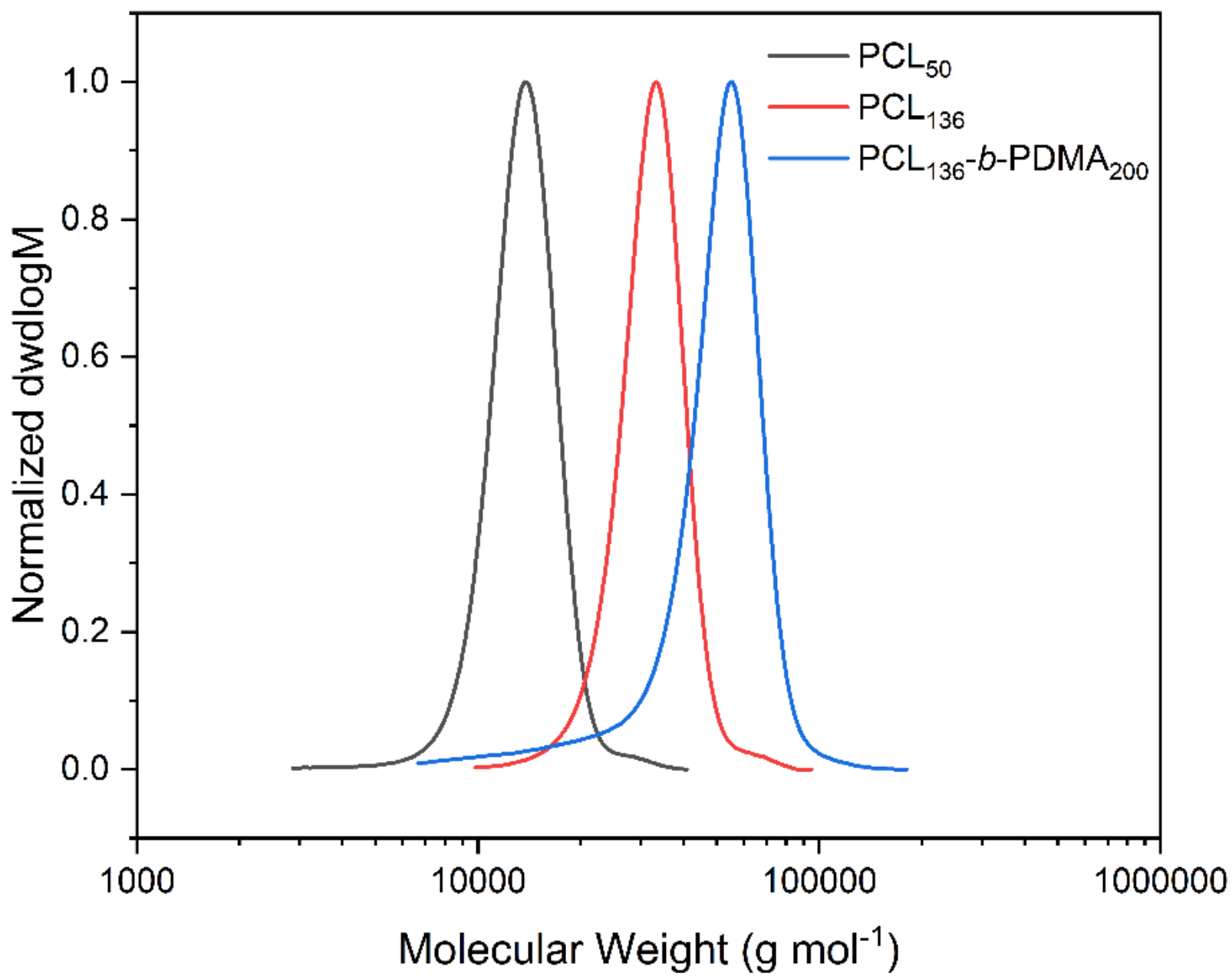

Figure S4. Apparent molar mass distribution of PCL_{50}, PCL_{136}, and PCL_{136}-*b*-$PDMA_{200}$.

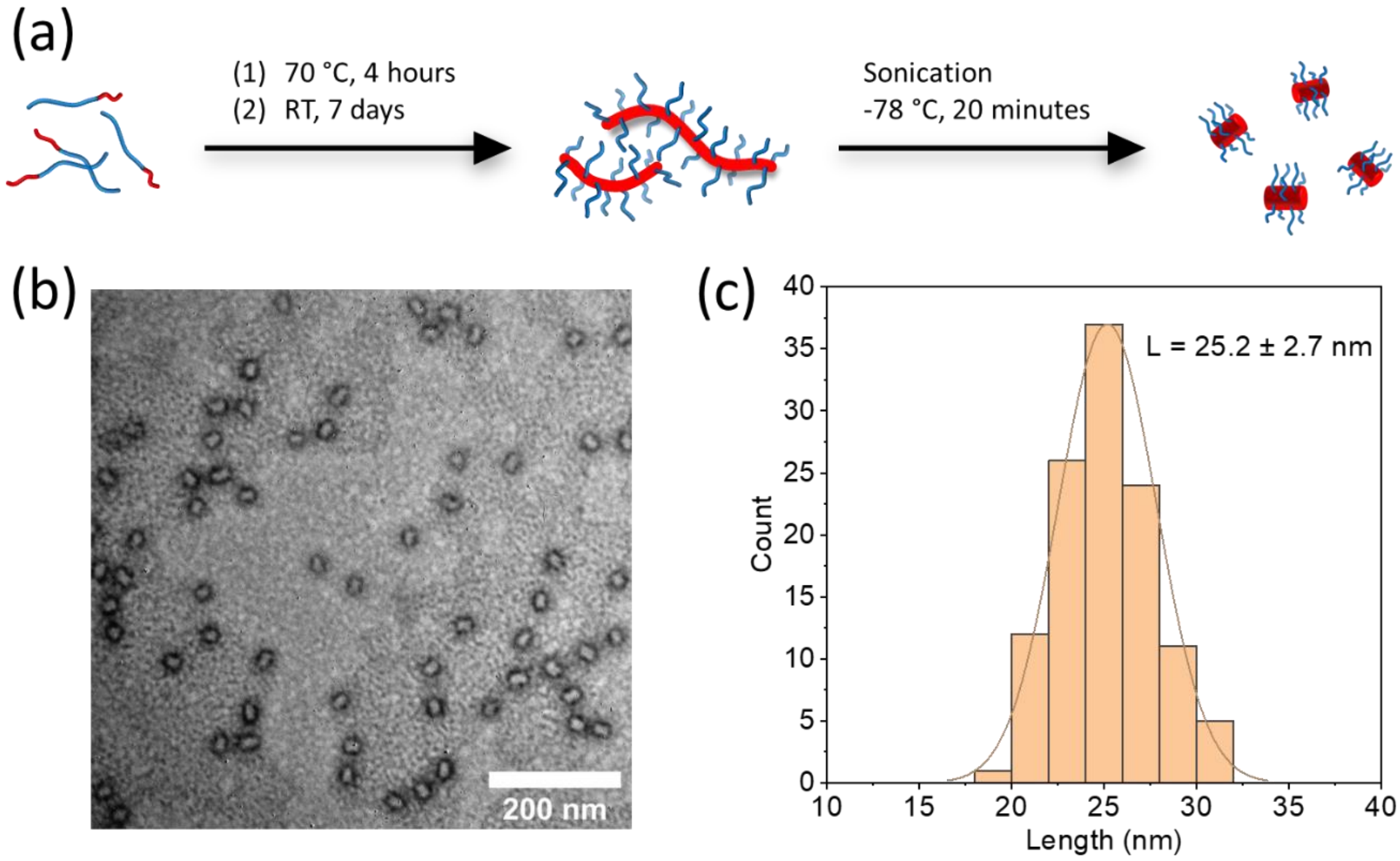

Figure S5. (a) Scheme to prepare seeds from PCL_{50}-*b*-$PDMA_{196}$. (b) TEM images of seeds. Uranyl acetate aqueous solution (1 wt%) was used as staining agent. (c) Length distribution of seeds. At least 100 particles were analysed to obtain statistical results.

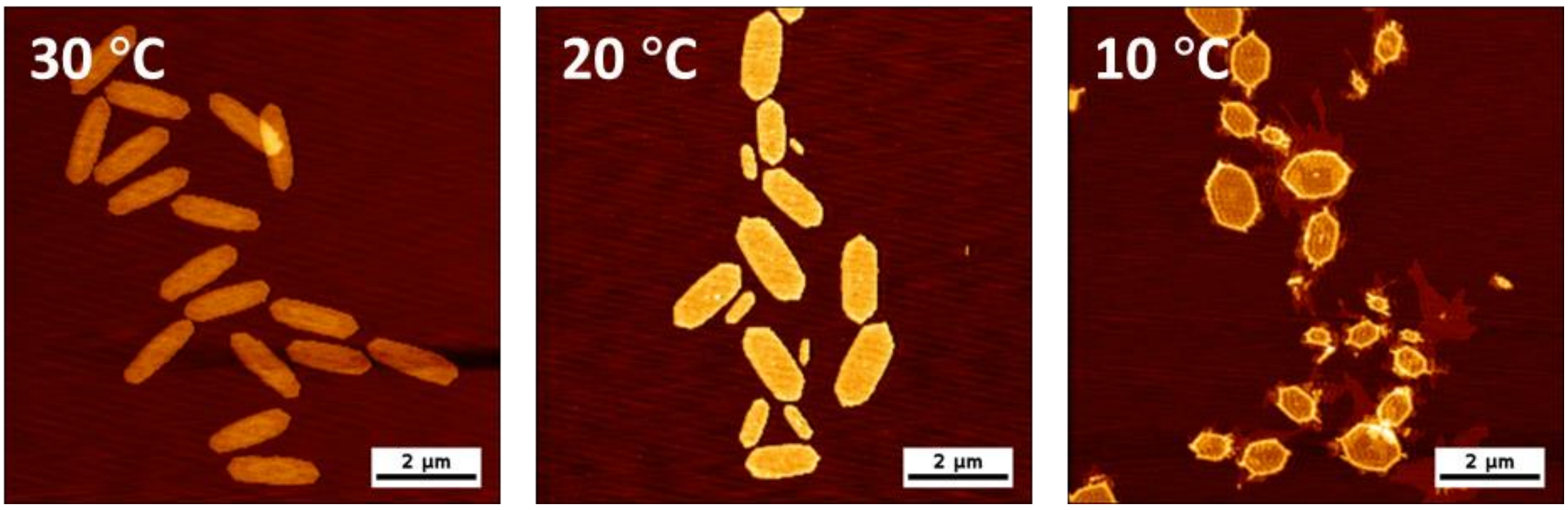

Figure S6. Low-magnification AFM images of a collection of platelets prepared at various temperatures (unimer: 1:1 wt % PCL_{50} / PCL_{136}-*b*-$PDMA_{200}$; unimer-to-seed ratio = 10). Scale bar = 2 μm.

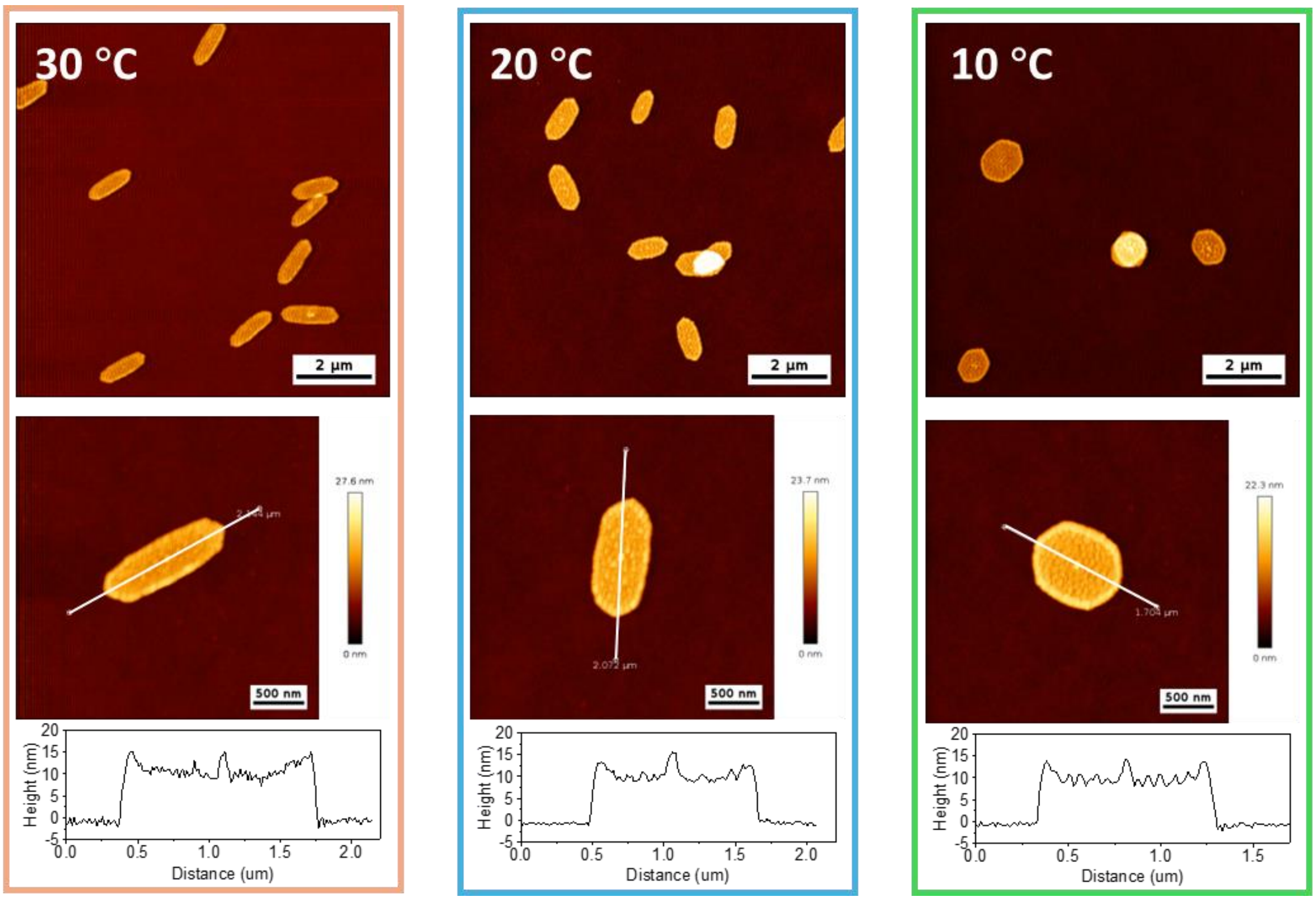

Figure S7. AFM images of batch platelets prepared at various temperatures using well-established PCL_{50} / PCL_{50}-*b*-$PDMA_{200}$ unimer (unimer: 1:1 wt % PCL_{50} / PCL_{50}-*b*-$PDMA_{200}$; unimer-to-seed ratio = 10). Scale bar = 2 μm (upper, low-magnification), and 500 nm (middle, magnified).

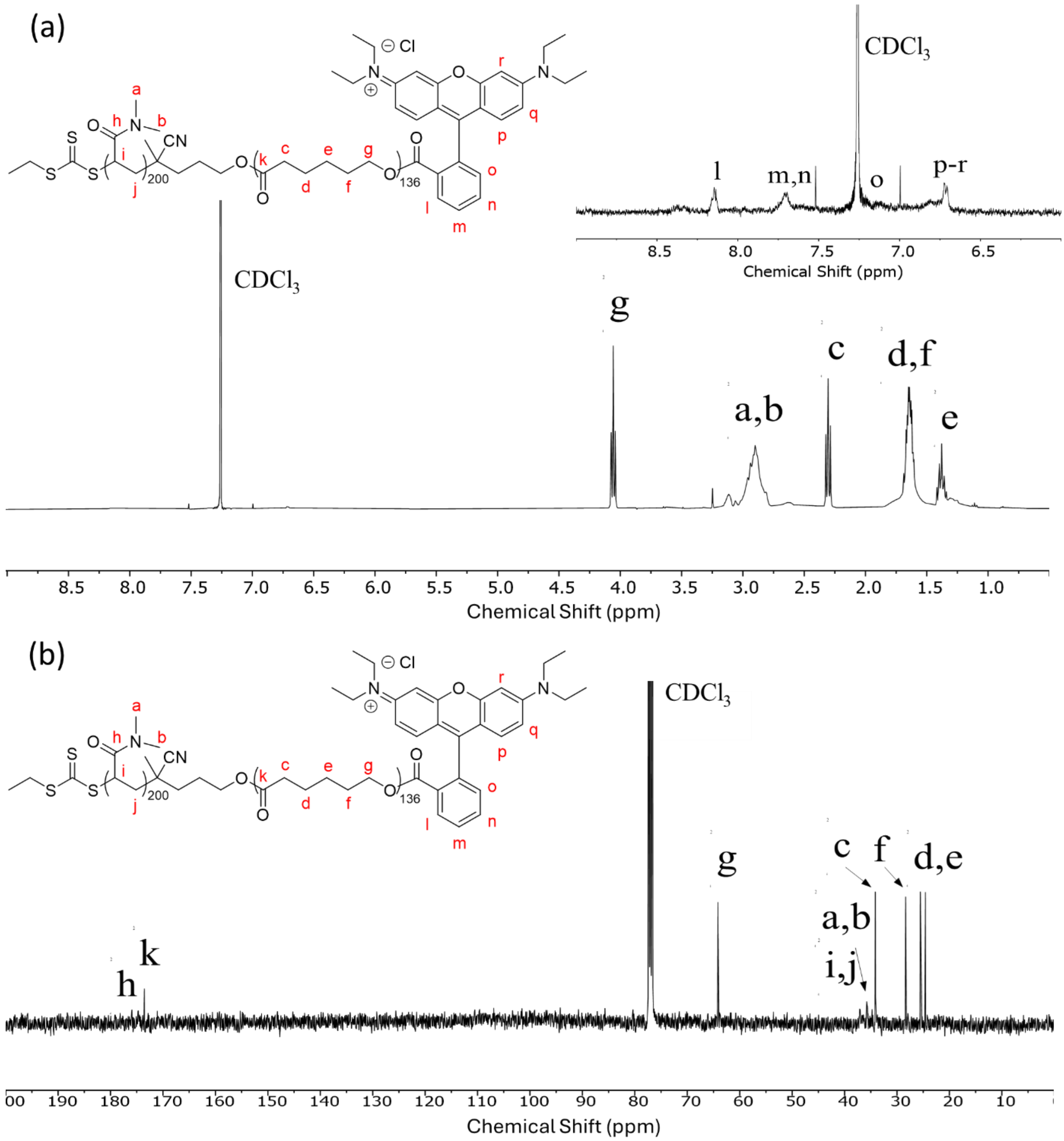

Figure S8. ${}^{1}H$ (a) and ${}^{13}C$ (b) NMR spectra of PCL_{136}-*b*-$PDMA_{200}$-RB (400 MHz; 298 K; in $CDCl_3$).

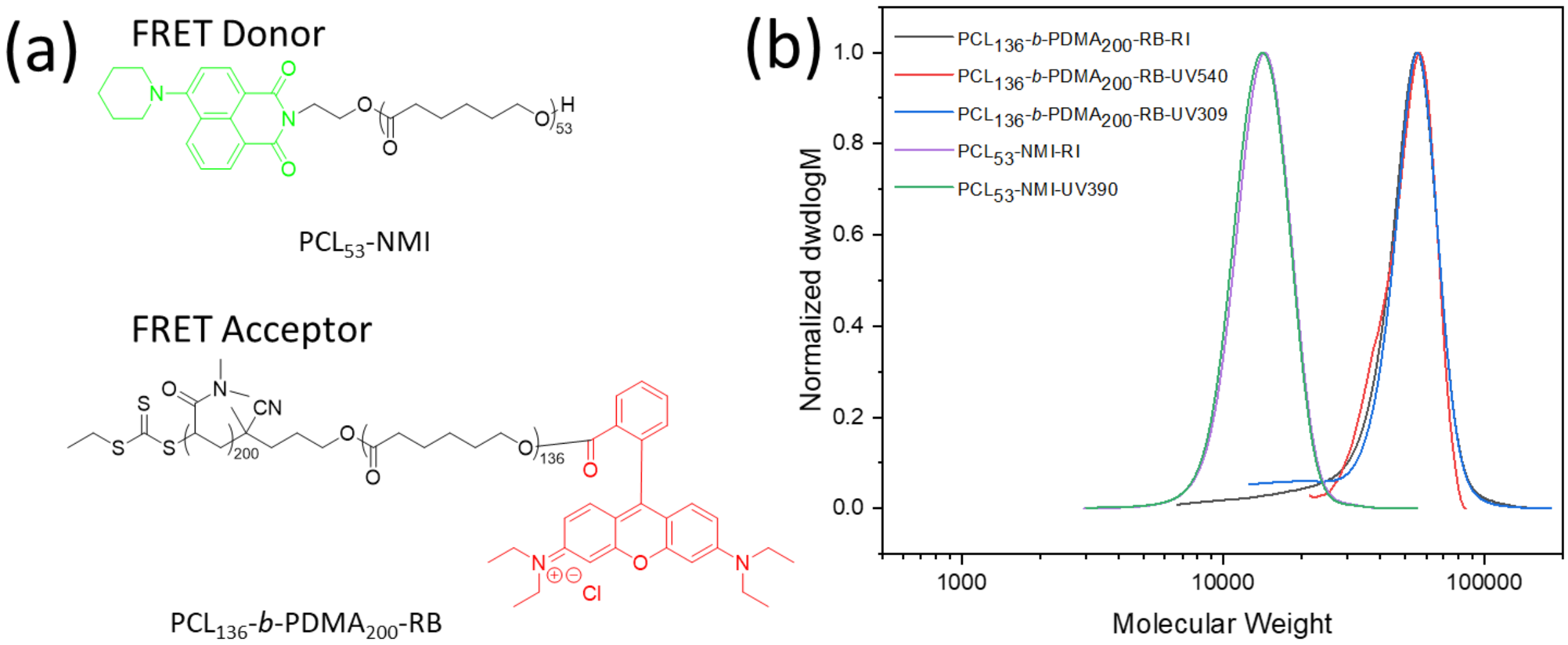

Figure S9. (a) Molecular structures of fluorescent dye-modified polymers, and (b) Apparent molar mass distribution of fluorescent dye-modified polymers detected by different signals.

(a)

	Excitation (nm)	Emission (nm)
PCL_{53}-NMI	408	537
PCL_{53}-*b*-$PDMA_{200}$-RB	558	580

(b)

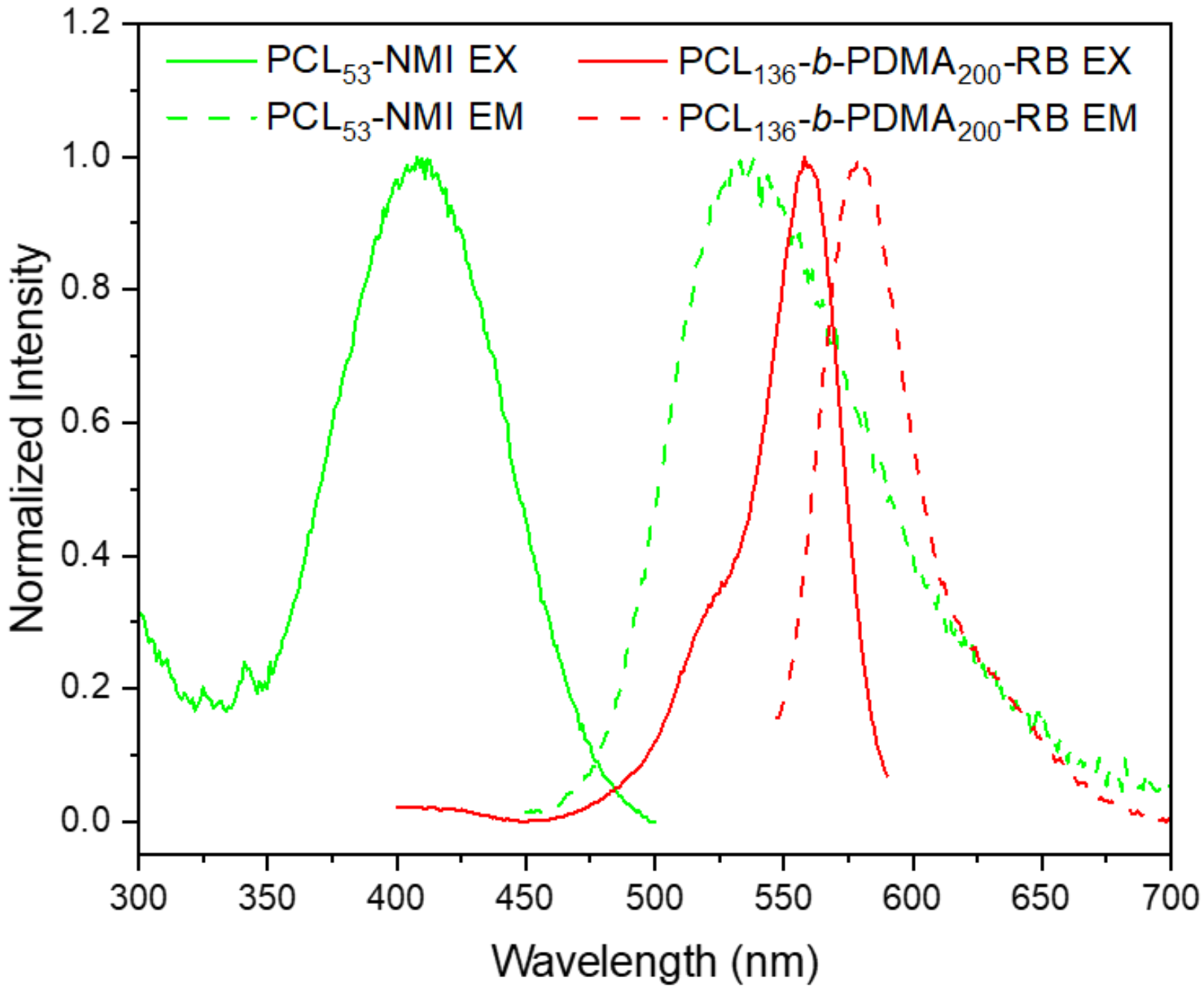

Figure S10. (a) Table of maximum excitation and emission wavelengths of fluorescent dye-modified polymers, and (b) Fluorescence spectra with solid lines for excitation and dashed lines for emission.

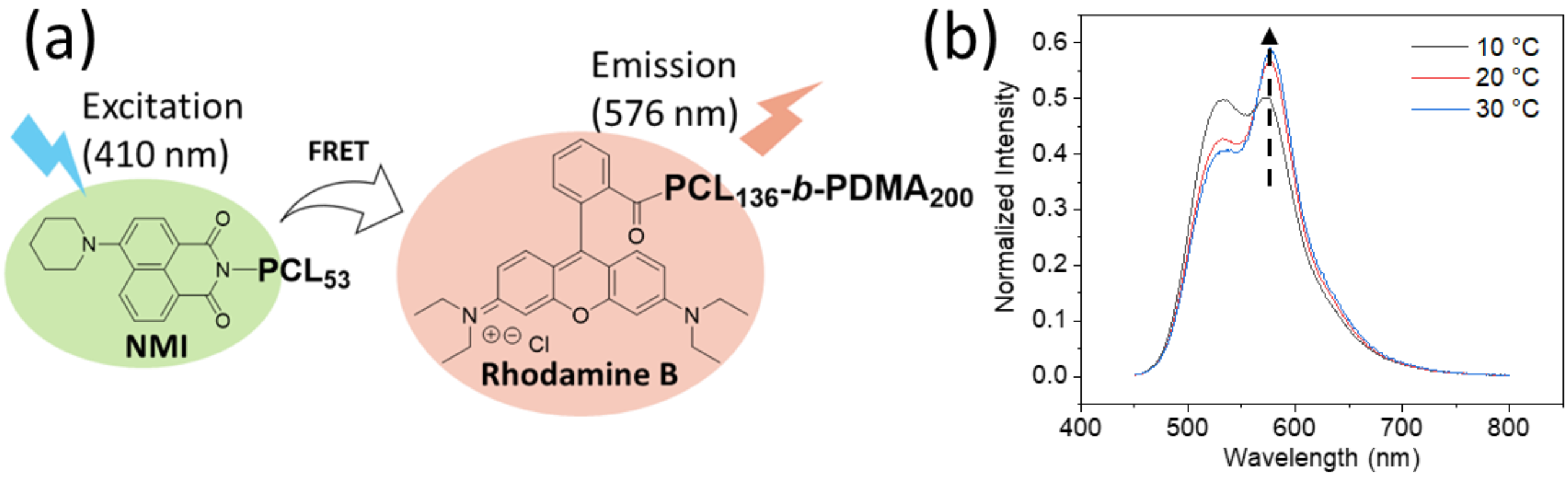

Figure S11. FRET on fluorescent dye-labelled platelets: (a) Schematic illustration of FRET between PCL_{53}-NMI (green) and PCL_{136}-*b*-$PDMA_{200}$-rhodamine B (red), and (b) Emission spectra of platelets excited at 410 nm, with peaks observed at λ = 530 nm (NMI) and λ = 575 nm (rhodamine B).

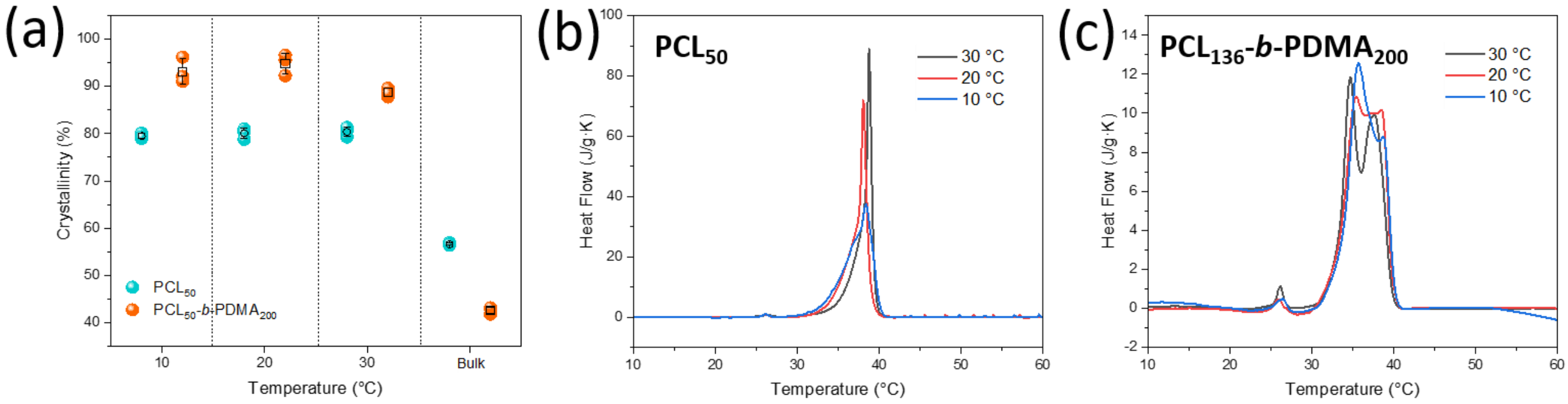

Figure S12. Thermodynamic analysis: (a) Comparison of the crystallinity of homopolymers and diblock copolymers crystallized at various temperatures in ethanol (Nano DSC) and in bulk (DSC), where data are presented as mean values +/- standard deviation (n = 3), and corresponding Nano DSC thermograms of (b) PCL_{50} and (c) PCL_{136}-*b*-$PDMA_{200}$ under those conditions.

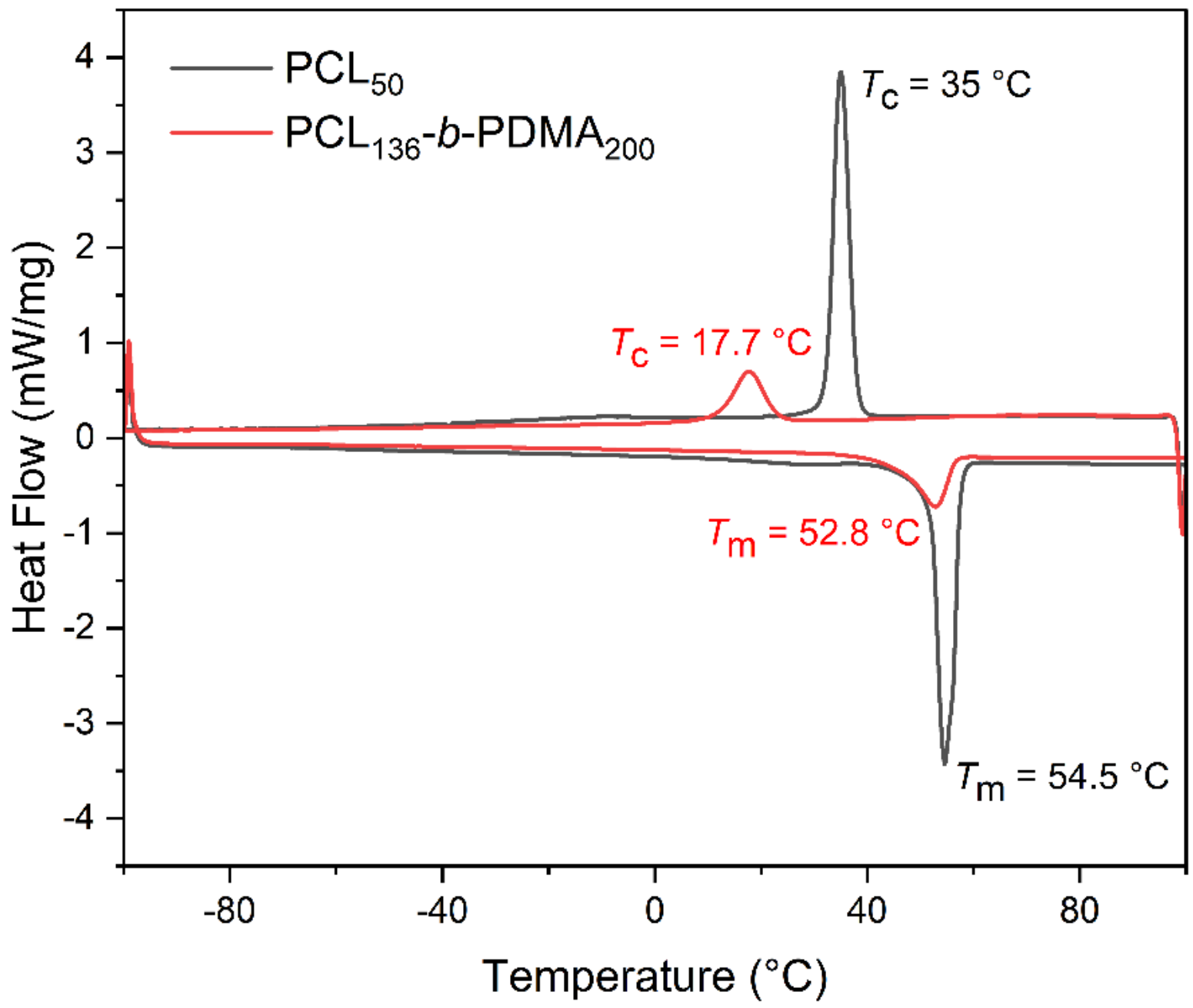

Figure S13. DSC thermograms of PCL_{50} and PCL_{136}-*b*-$PDMA_{200}$.

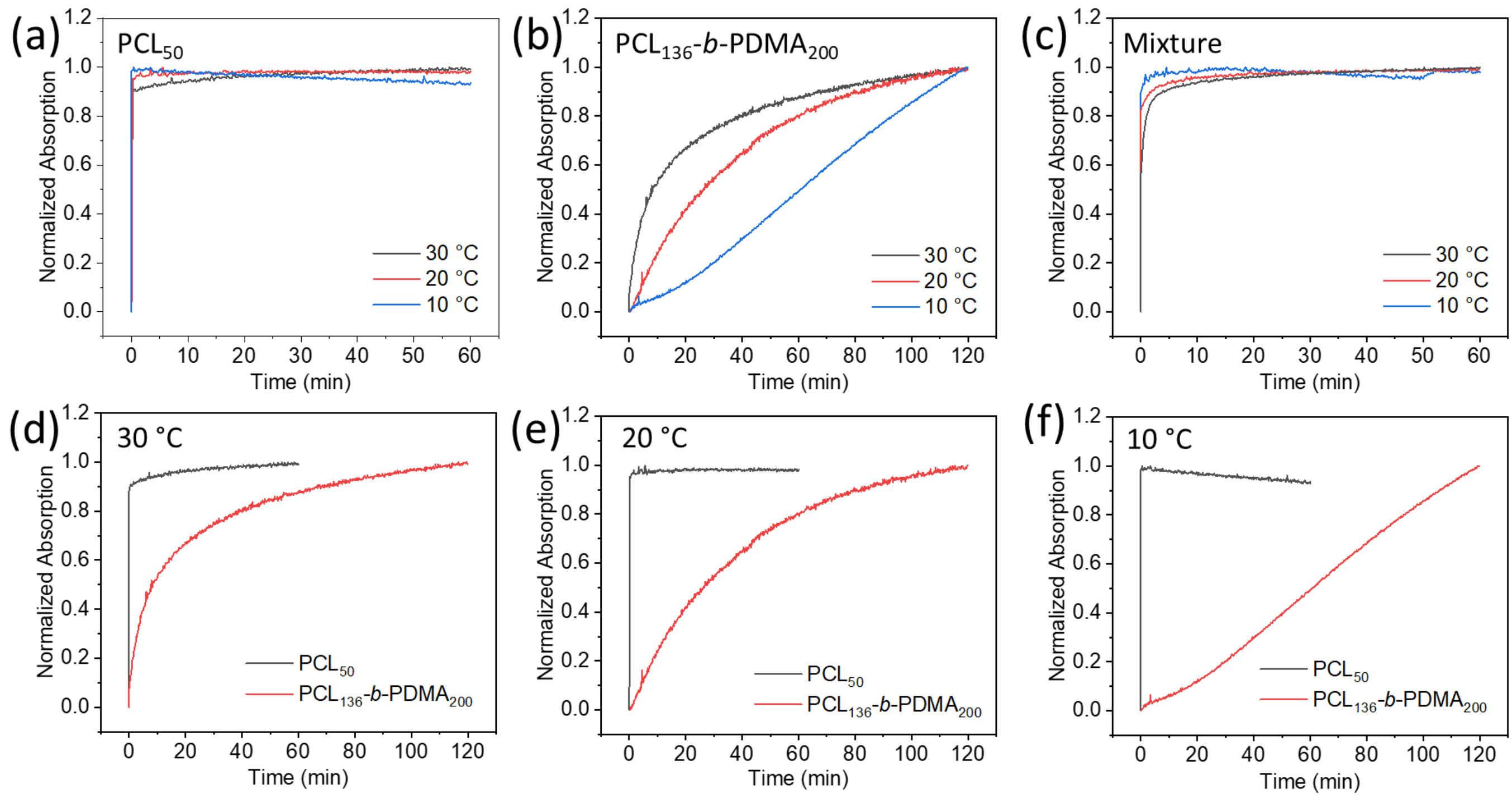

Figure S14. UV-Vis spectra used to monitor seeded growth kinetics by tracking signal intensity at 500 nm over time: (a)-(c) growth kinetics of PCL_{50}, PCL_{136}-*b*-$PDMA_{200}$, and a mixture of PCL_{50}/PCL_{136}-*b*-$PDMA_{200}$ (1:1 wt%), respectively, at various temperatures, and (d)-(f) comparison of the growth kinetics of PCL_{50} and PCL_{136}-*b*-$PDMA_{200}$ at 30, 20, and 10 °C respectively. All measurements were performed at a concentration of 0.1 mg mL^{-1}, except for PCL_{136}-*b*-$PDMA_{200}$ at 10 °C, which was conducted at 1 mg mL^{-1} due to the slow kinetics and weak UV–Vis signal changes observed at lower concentration.

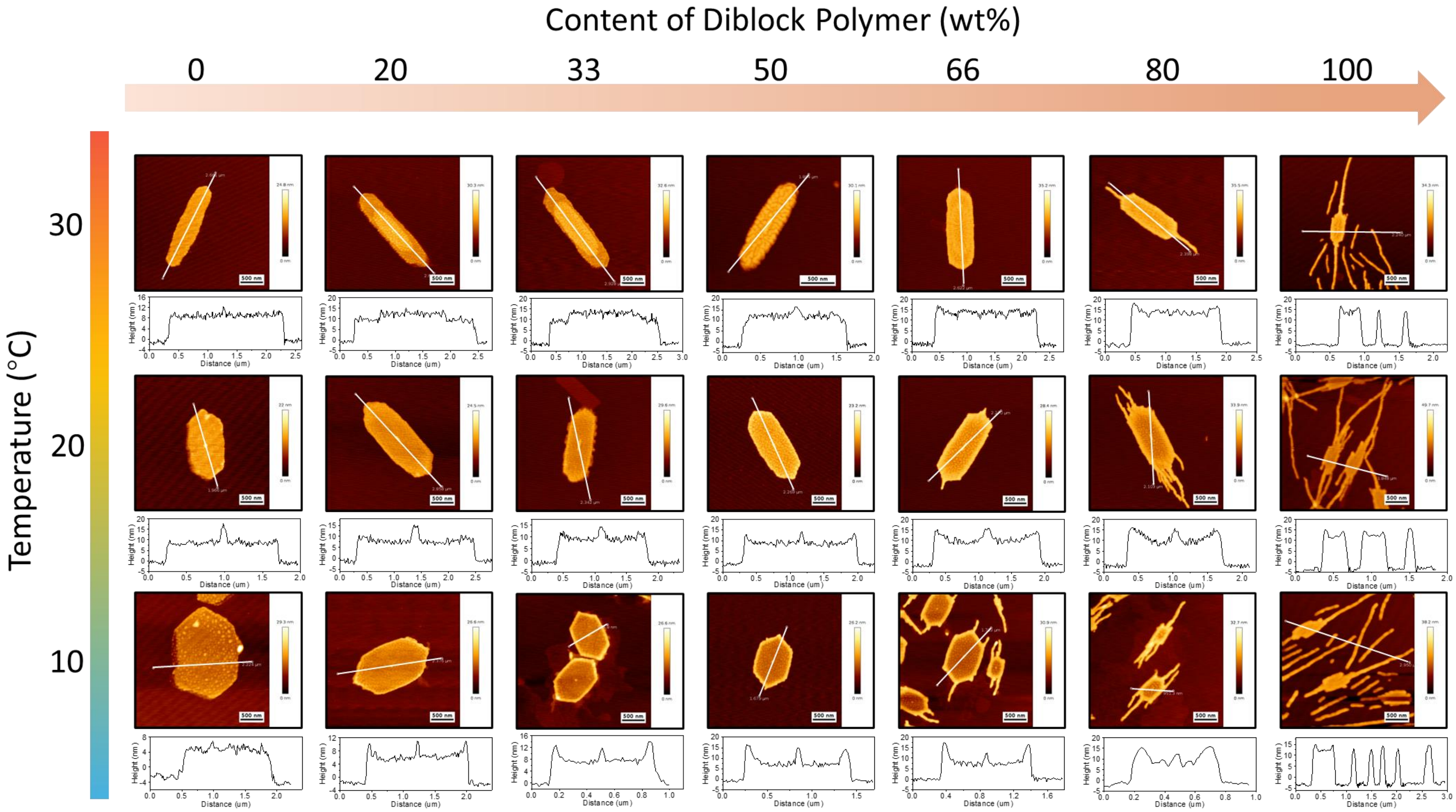

Figure S15. High-magnification AFM images of a single platelet showing the morphological variation as a function of temperature and the content of diblock copolymer (unimer-to-seed ratio = 10). Scale bar = 500 nm.

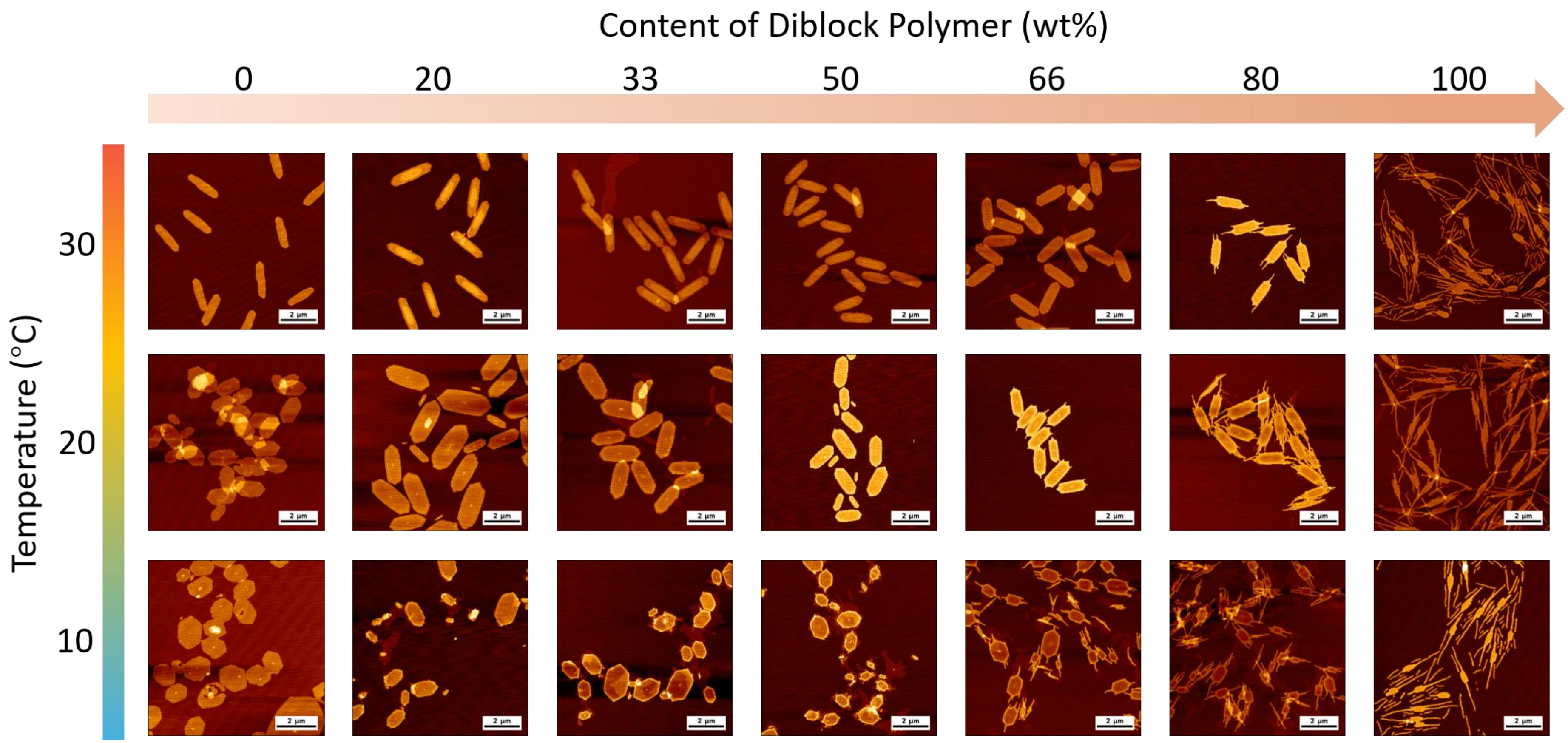

Figure S16. Low-magnification AFM images of a collection of platelets showing morphological variation as a function of temperature and the content of diblock copolymer (unimer-to-seed ratio = 10). Scale bar = 2 µm.

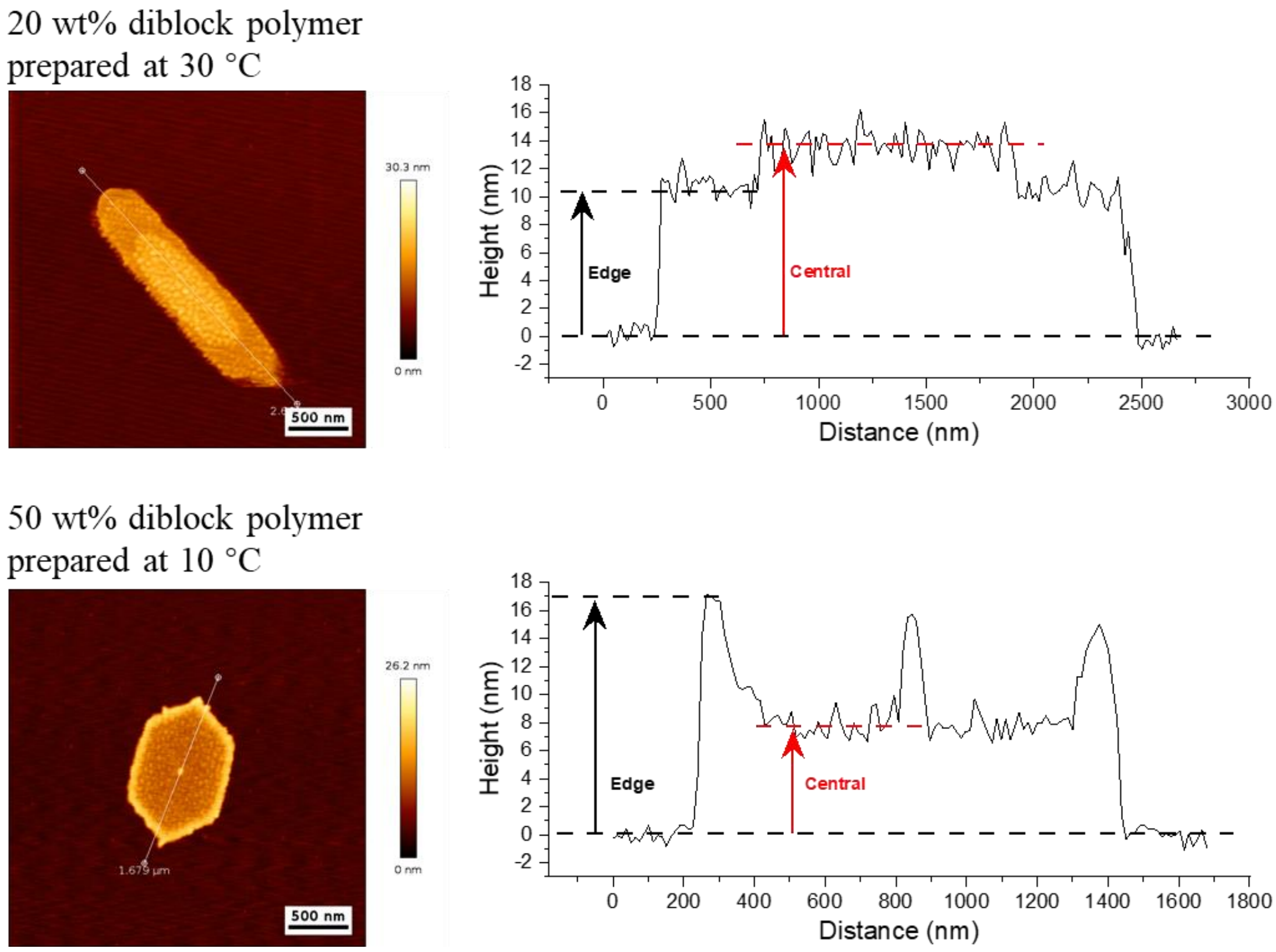

Figure S17. AFM images of platelets and corresponding height profiles illustrating center and edge height measurements for concave (lower) and convex (upper) surface morphologies.

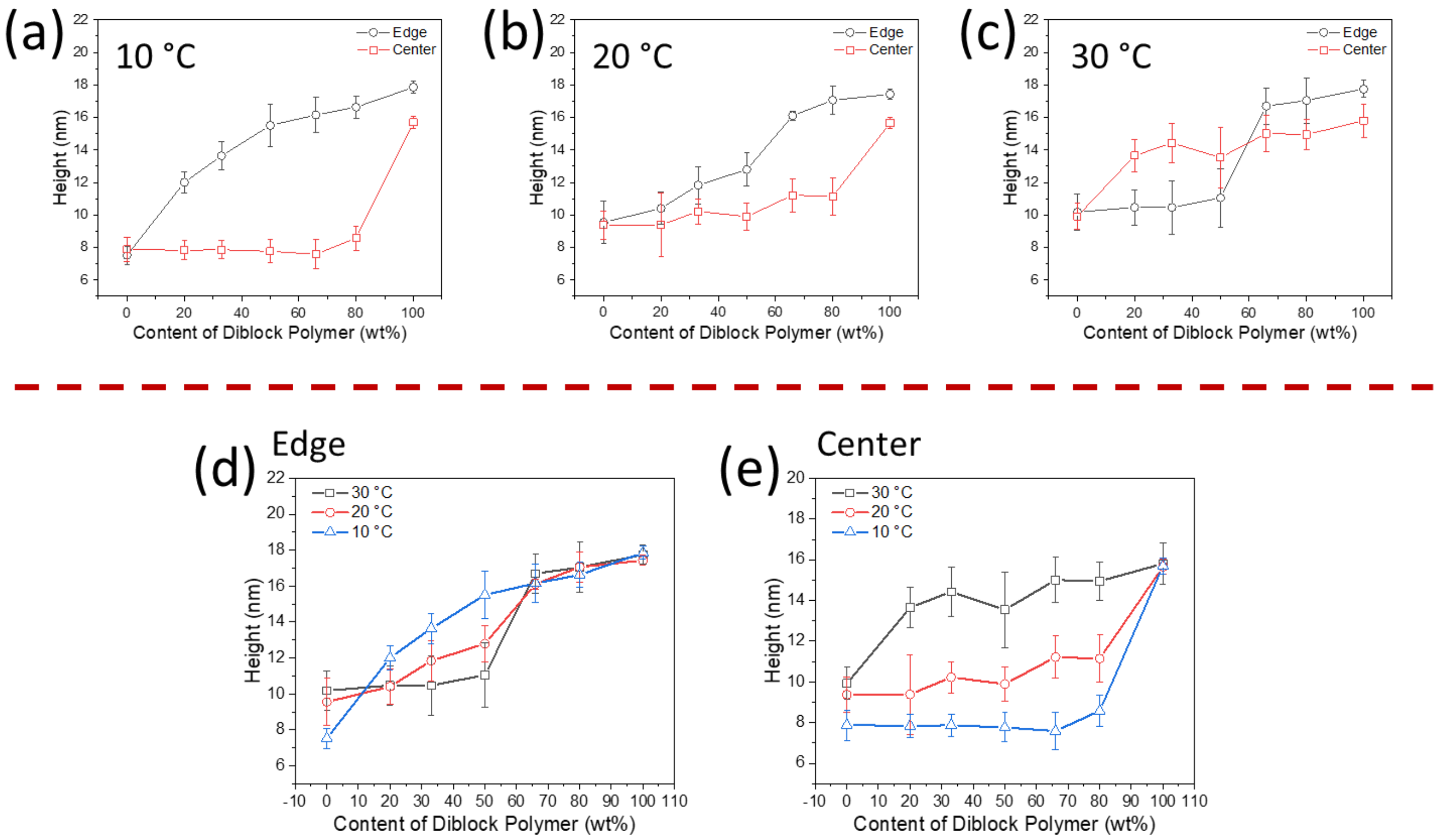

Figure S18. Height variation as a function of diblock copolymer content: (a)-(c) Height variation at the edge and center of platelets prepared at 10, 20, and 30 °C respectively, (d) Comparison of edge height variation across temperatures, and (e) Comparison of center height variation across temperatures. Data are presented as mean values +/- standard deviation of height values calculated from all data points within the plateau region of each height profile.

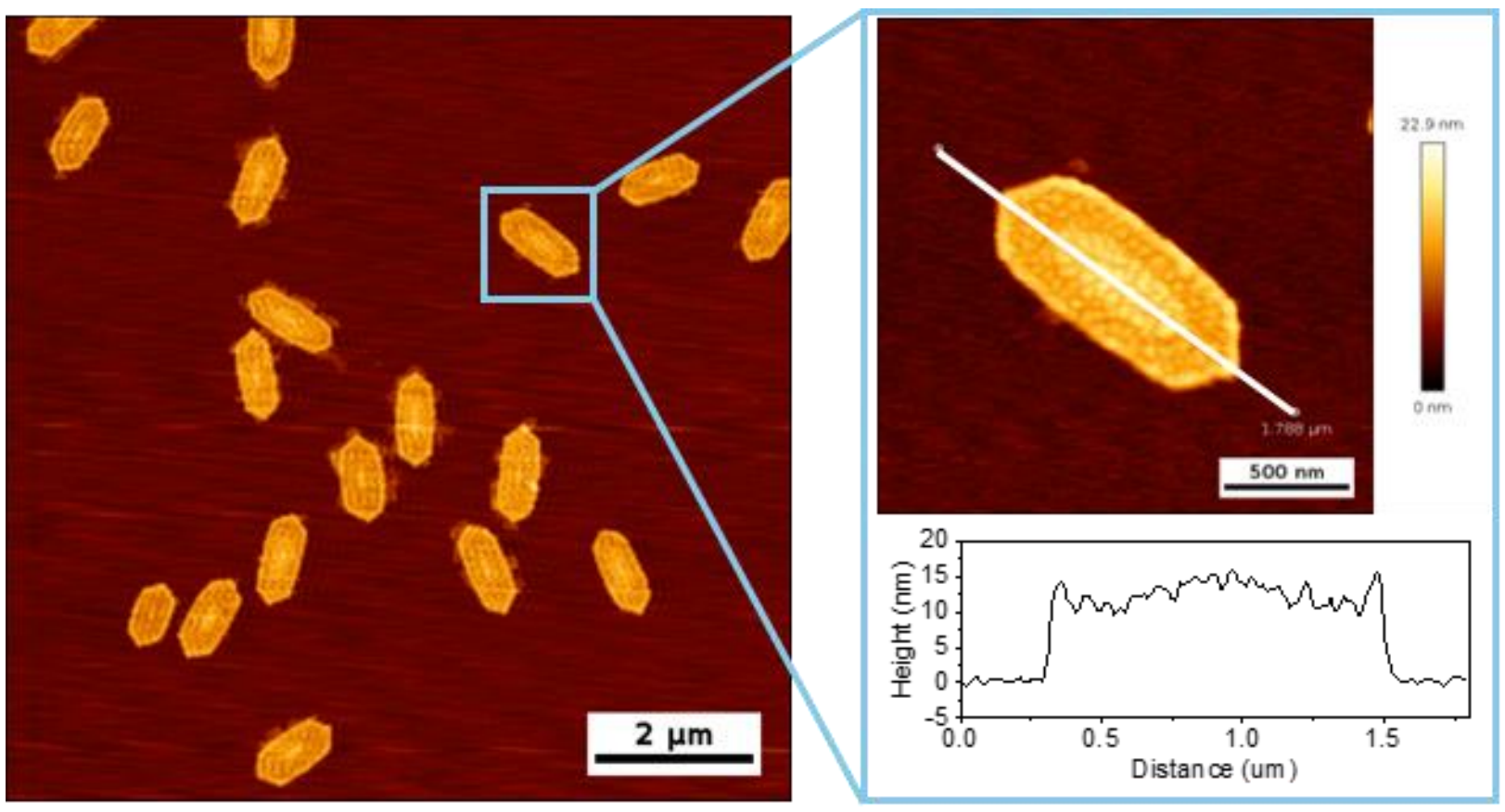

Figure S19. AFM images of platelets prepared in batch during an initial attempt to segment the living growth period by rapid temperature switching. After unimer addition to a 1 mL seed solution in a 2 mL glass vial, the mixture was aged at 30 °C for 10 s and subsequently at 10 °C for 2 min by rapidly transferring between water baths.

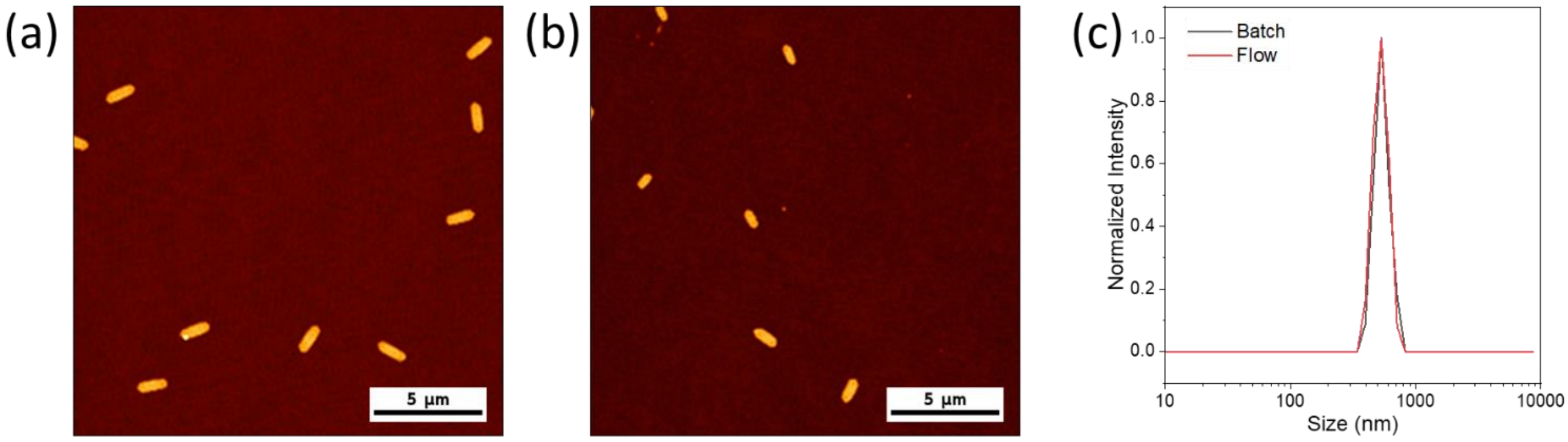

Figure S20. Comparison of platelet samples prepared from diluted seeds (0.0025 mg mL^{-1}) and unimer (1:1 wt % PCL_{50} / PCL_{136}-*b*-$PDMA_{200}$; 5 mg mL^{-1} in $CHCl_3$; unimer-to-seed ratio = 10) under batch and flow conditions: (a) AFM image of platelets prepared in batch, (b) AFM image of platelets prepared in flow, and (c) DLS size distributions of the resulting platelets based on scattering intensity. Both AFM and DLS results indicate that seeds were fully converted to platelets.

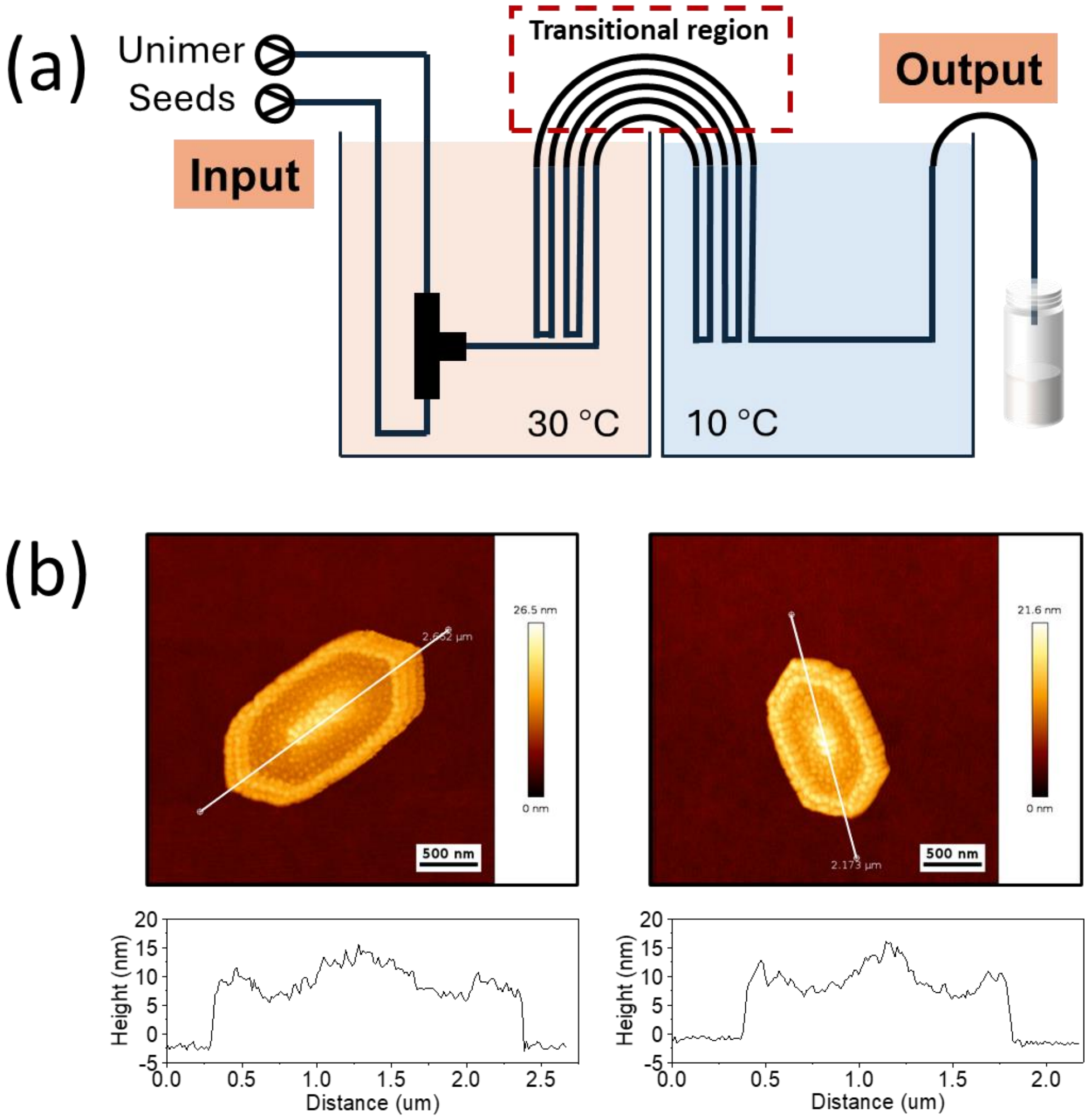

Figure S21. Layered platelets prepared under conditions lack rapid temperature-switching capability: (a) Simplfied flow scheme illustrating shuttling of tubing between two water baths at different temperatures and the resulting unavoidable transitional regions, and (b) AFM images of the resulting platelets lacking well-defined layered structures.

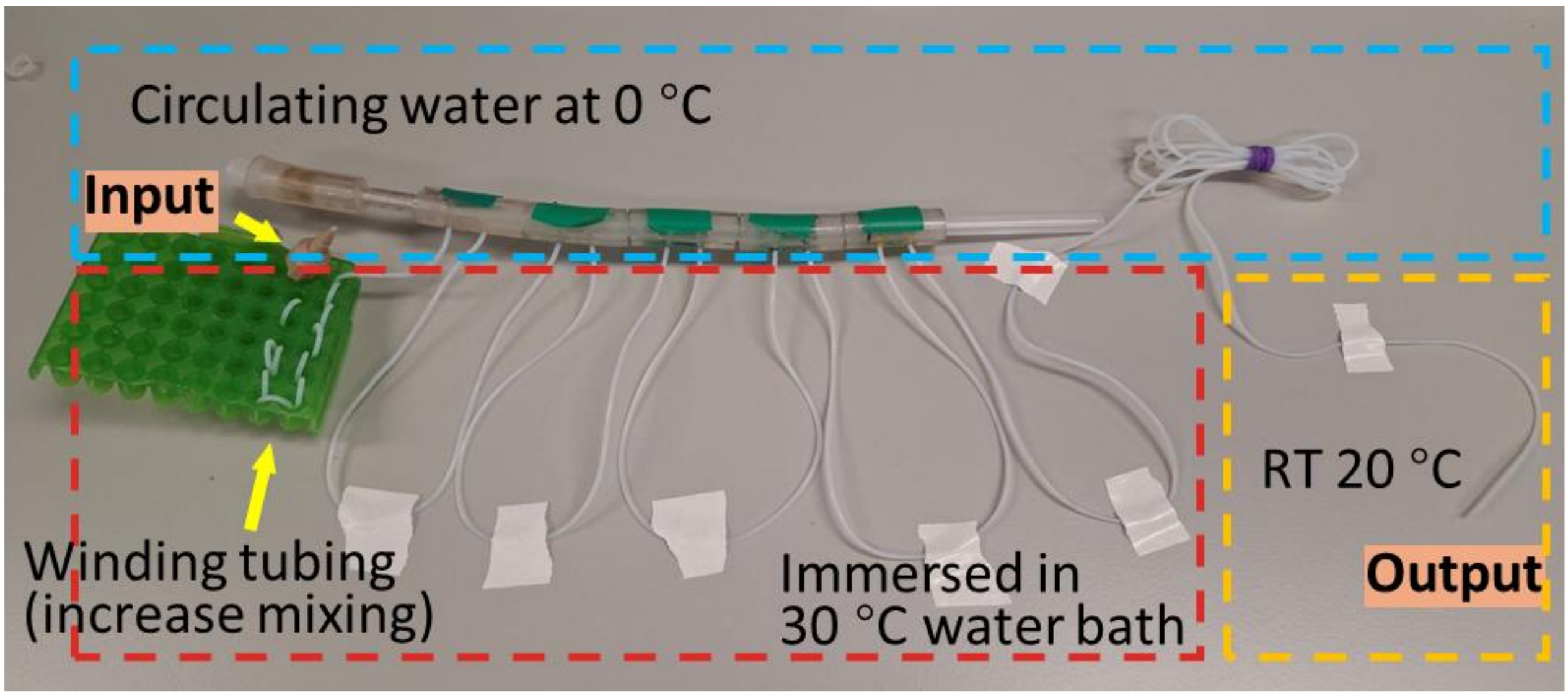

Figure S22. Photograph of flow setup to prepare platelets with layered patterns by rapid temperature switching in flow.

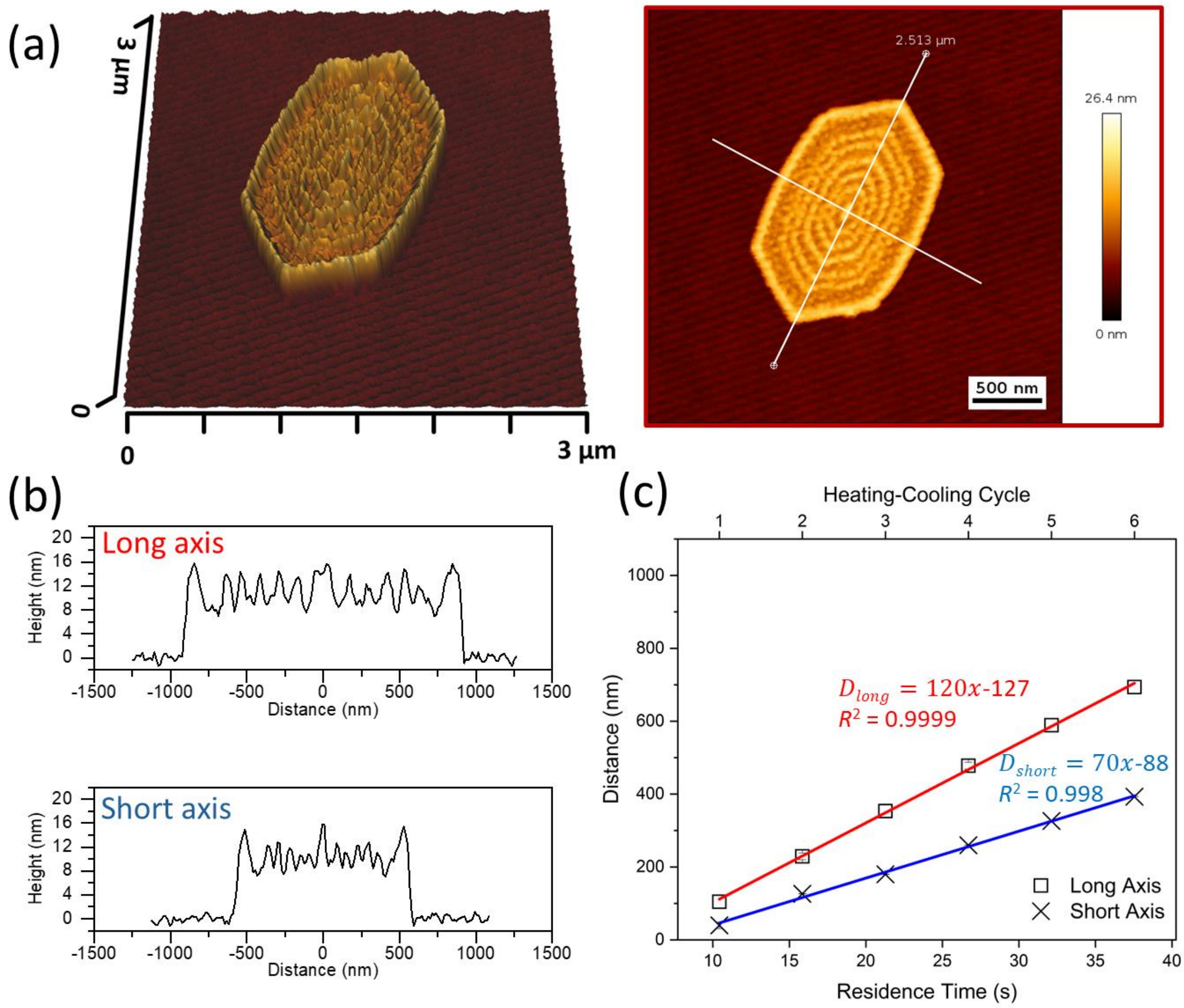

Figure S23. Additional kinetic analysis (Case 1): (a) AFM image of 13-layer platelets, shown as 3D rendering (left) and 2D height map (right), (b) Height profiles along the long (upper) and short (bottom) axis of platelets, and (c) Linear epitaxial growth of platelets. In the fitted equation, *D* represents the axial growth distance and *x* denotes the heating-cooling cycle. Date was collected at the troughs of height profiles. Scale bar = 500 nm.

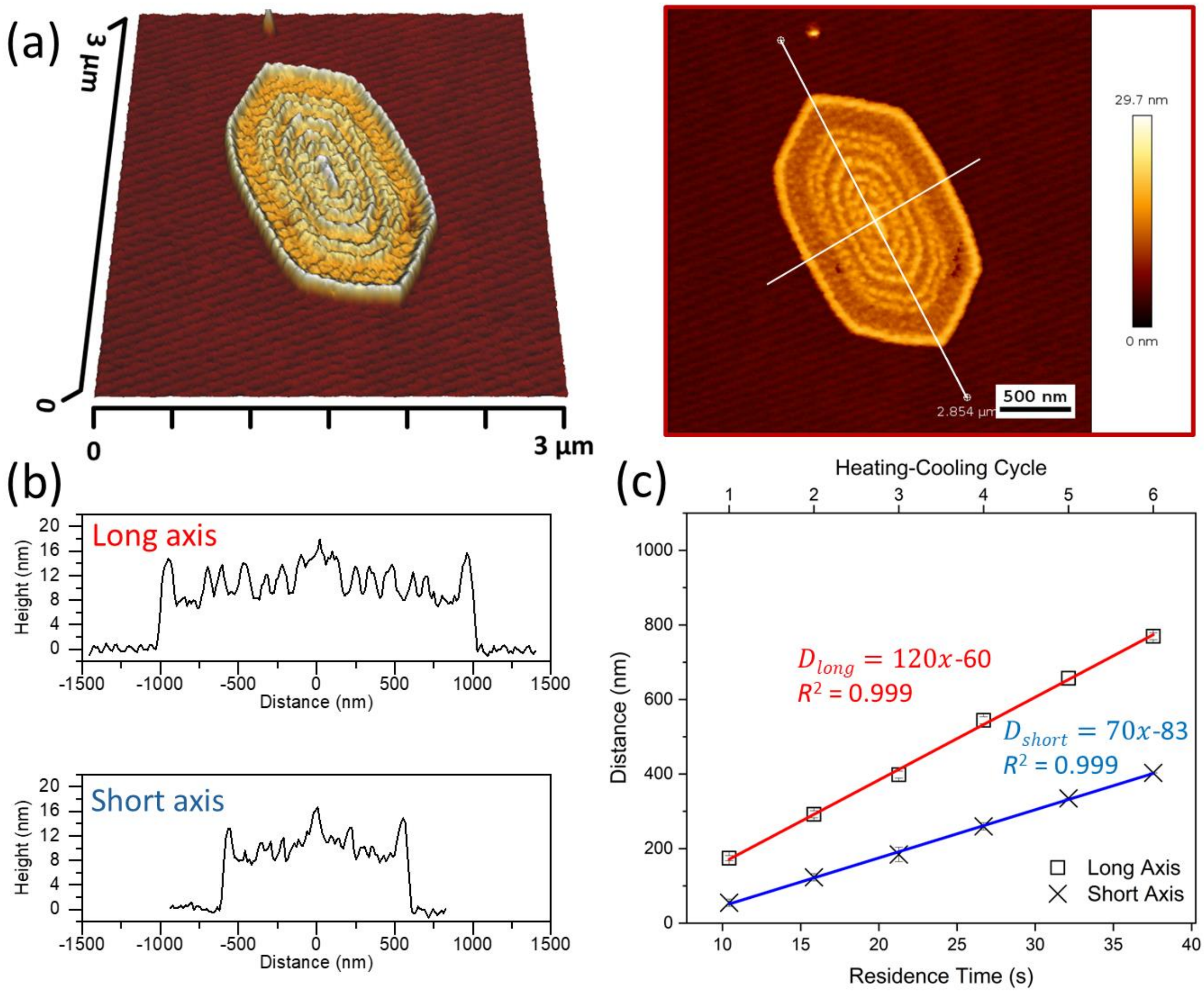

Figure S24. Additional kinetic analysis (Case 2): (a) AFM image of 13-layer platelets, shown as 3D rendering (left) and 2D height map (right), (b) Height profiles along the long (upper) and short (bottom) axis of platelets, and (c) Linear epitaxial growth of platelets. In the fitted equation, *D* represents the axial growth distance and *x* denotes the heating-cooling cycle. Date was collected at the troughs of height profiles. Scale bar = 500 nm.

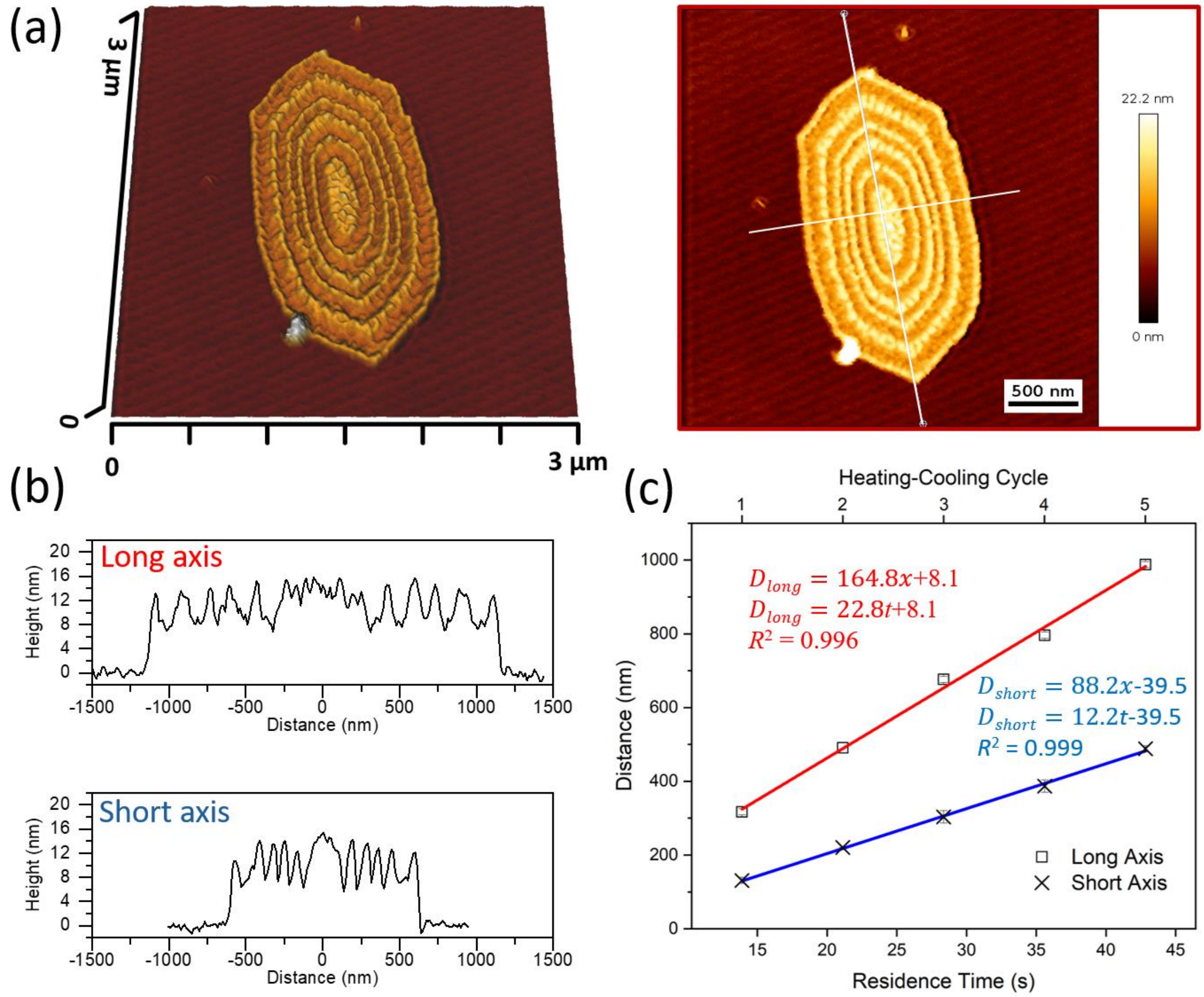

Figure S25. Kinetic analysis of platelets prepared at 150 µl min^{-1}: (a) AFM image of 11-layer platelets, shown as 3D rendering (left) and 2D height map (right), (b) Height profiles along the long (upper) and short (bottom) axis of platelets, and (c) Linear epitaxial growth of platelets. In the fitted equation, *D* represents the axial growth distance, *x* denotes the heating-cooling cycle, and *t* represents growth time. Date was collected at the troughs of height profiles. Scale bar = 500 nm.

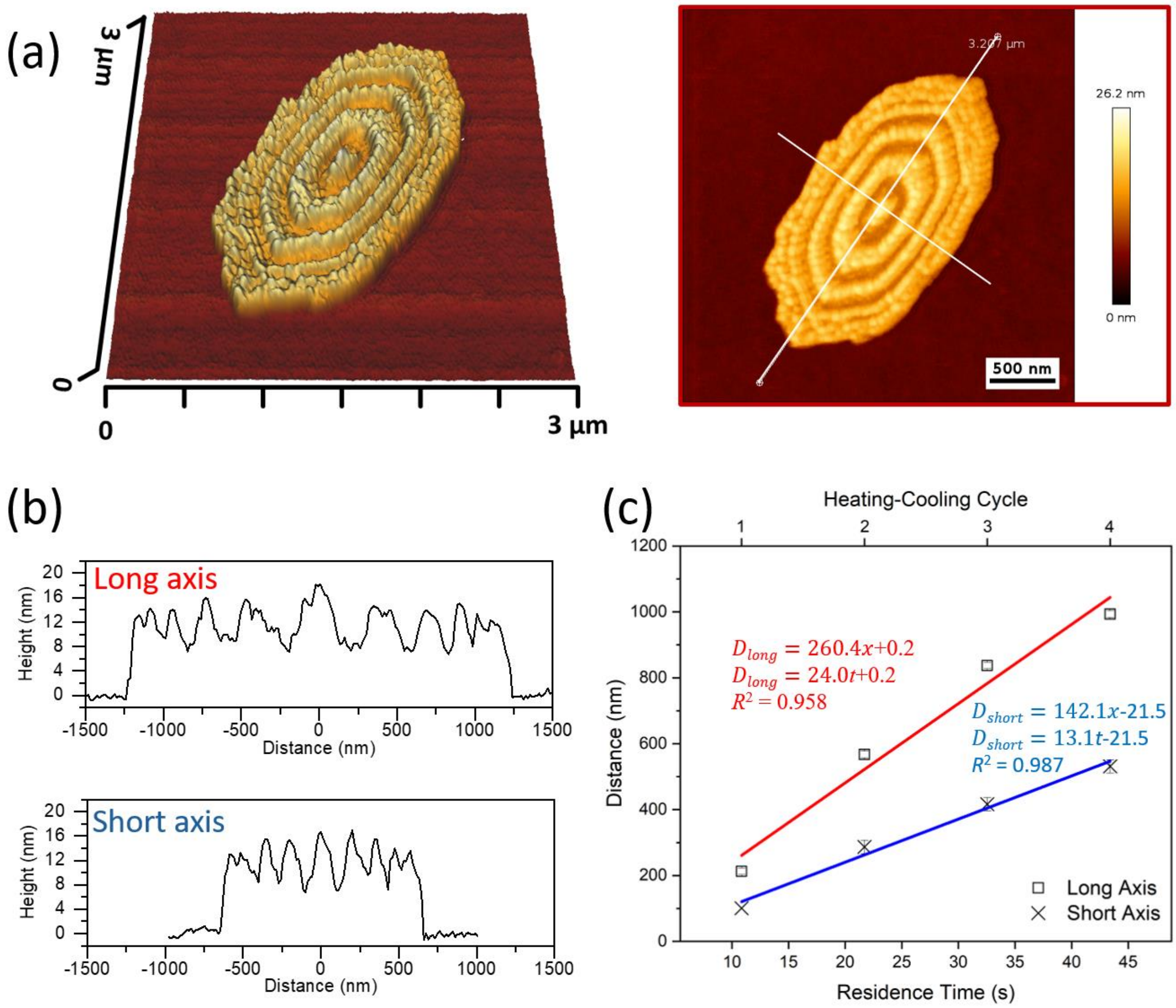

Figure S26. Kinetic analysis of platelets prepared at 100 µl min^{-1}: (a) AFM image of 9-layer platelets, shown as 3D rendering (left) and 2D height map (right), (b) Height profiles along the long (upper) and short (bottom) axis of platelets, and (c) Linear epitaxial growth of platelets. In the fitted equation, D represents the axial growth distance, x denotes the heating-cooling cycle, and t represents growth time. Date was collected at the troughs of height profiles. Scale bar = 500 nm. To facilitate the formation of sufficient layers for kinetic studies at a flow rate of 100 µl min^{-1}, the residence time of the first layer was maintained at 10 s.

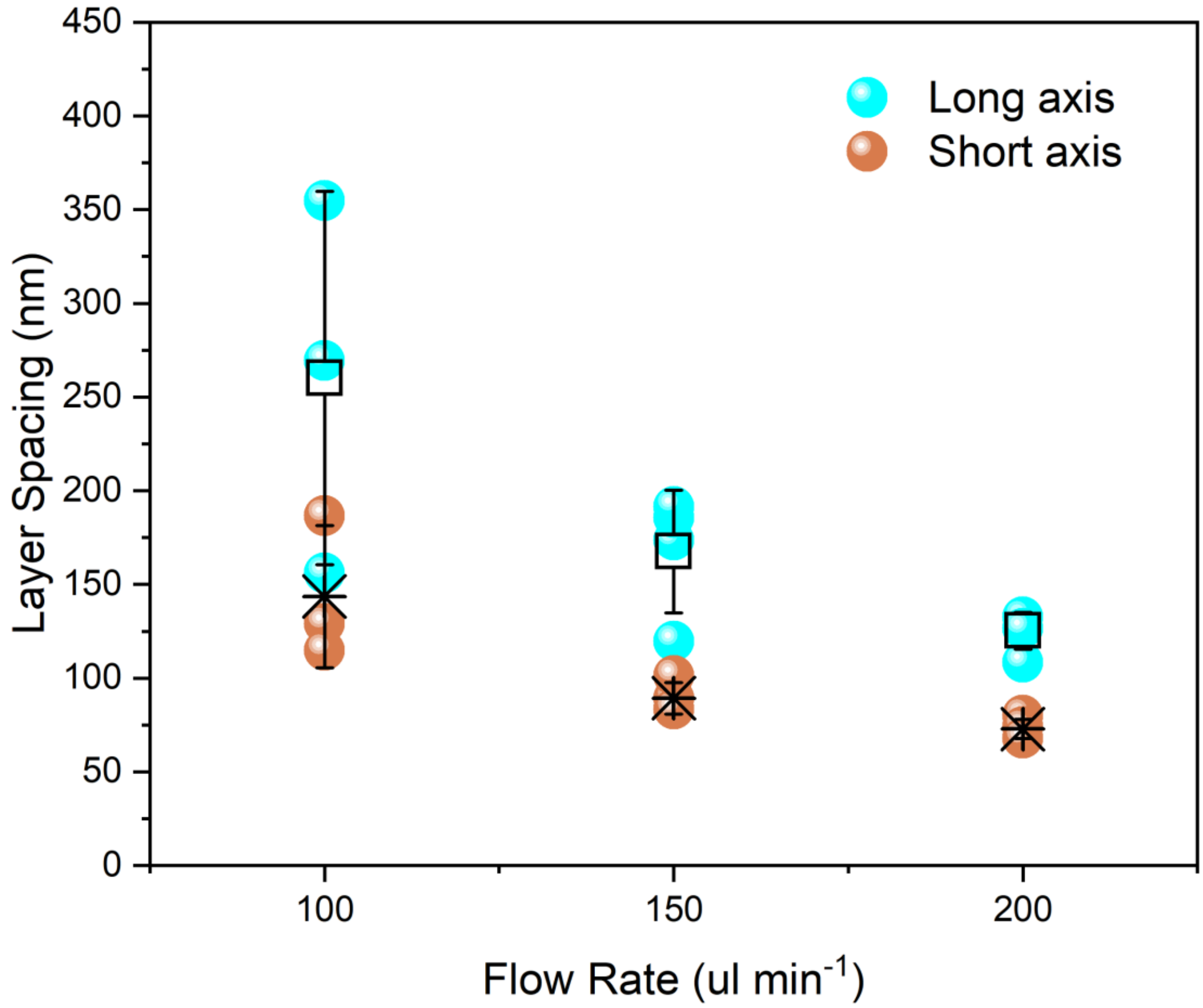

Figure S27. Layer distances of platelets prepared at various flow rates. Data are presented as mean values +/- standard deviation (n ≥ 3).

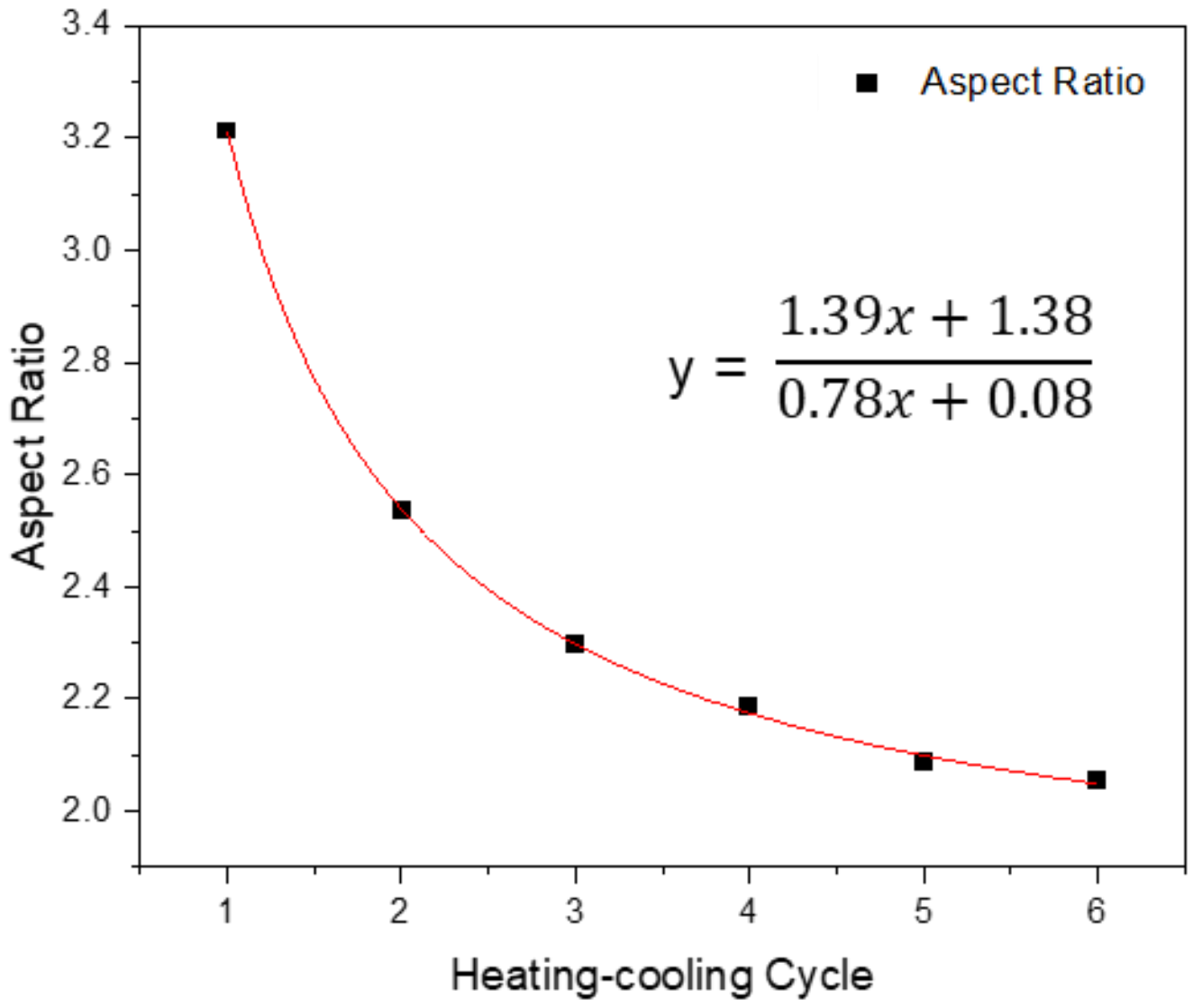

Model	NewFunction2 (User)
Equation	(A*x+B)/(C*x+D)
Plot	Aspect Ratio
A	1.39416 ± 7675.24735
B	1.37887 ± 7593.99152
C	0.77855 ± 4307.61912
D	0.08477 ± 465.70447
Reduced Chi-Sqr	1.67238E-4
R-Square (COD)	0.99965
Adj. R-Square	0.99912

Figure S28. Aspect ratio variation versus heating cooling cycle. Aspect ratio variation was fitted using Origin (OriginLab) with the model y = (A*X+B)/(C*X+D), based on the linearity of directional growth rates. Original data with fitted curves are shown on the left, and fitting results are presented on the right. The relatively large deviations associated with the individual fitting parameters arise from strong parameter correlation within the rational fitting function, where multiple parameter combinations can produce nearly identical fitting curves. Therefore, the fitting was primarily used to describe the overall trend and limiting behavior of the aspect ratio variation rather than to extract physically meaningful individual fitting parameters.

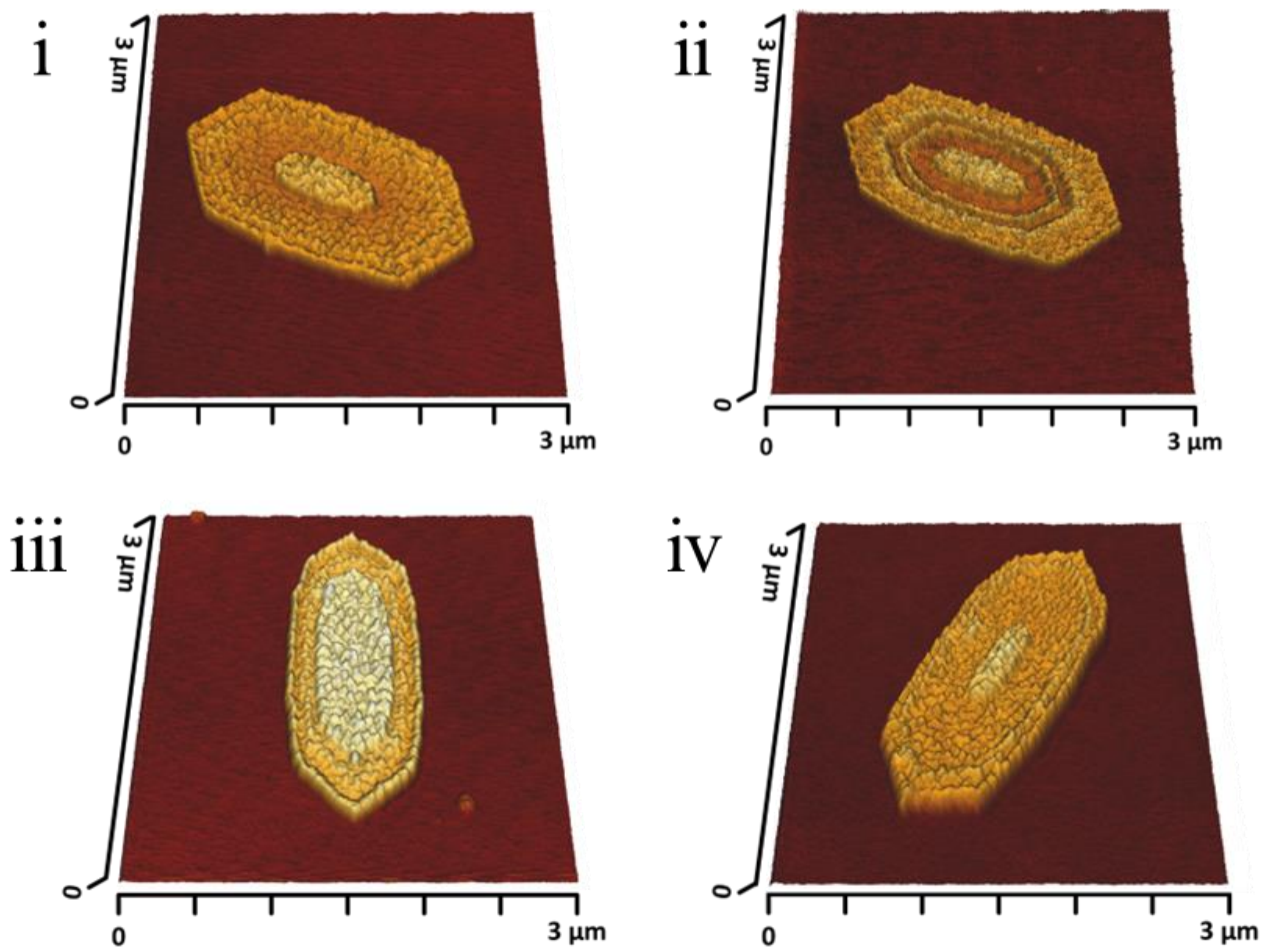

Figure S29. 3D AFM images of platelets prepared under various programmed temperature and residence time conditions, corresponding to the flow schemes and 2D AFM images shown in Figure 4d.

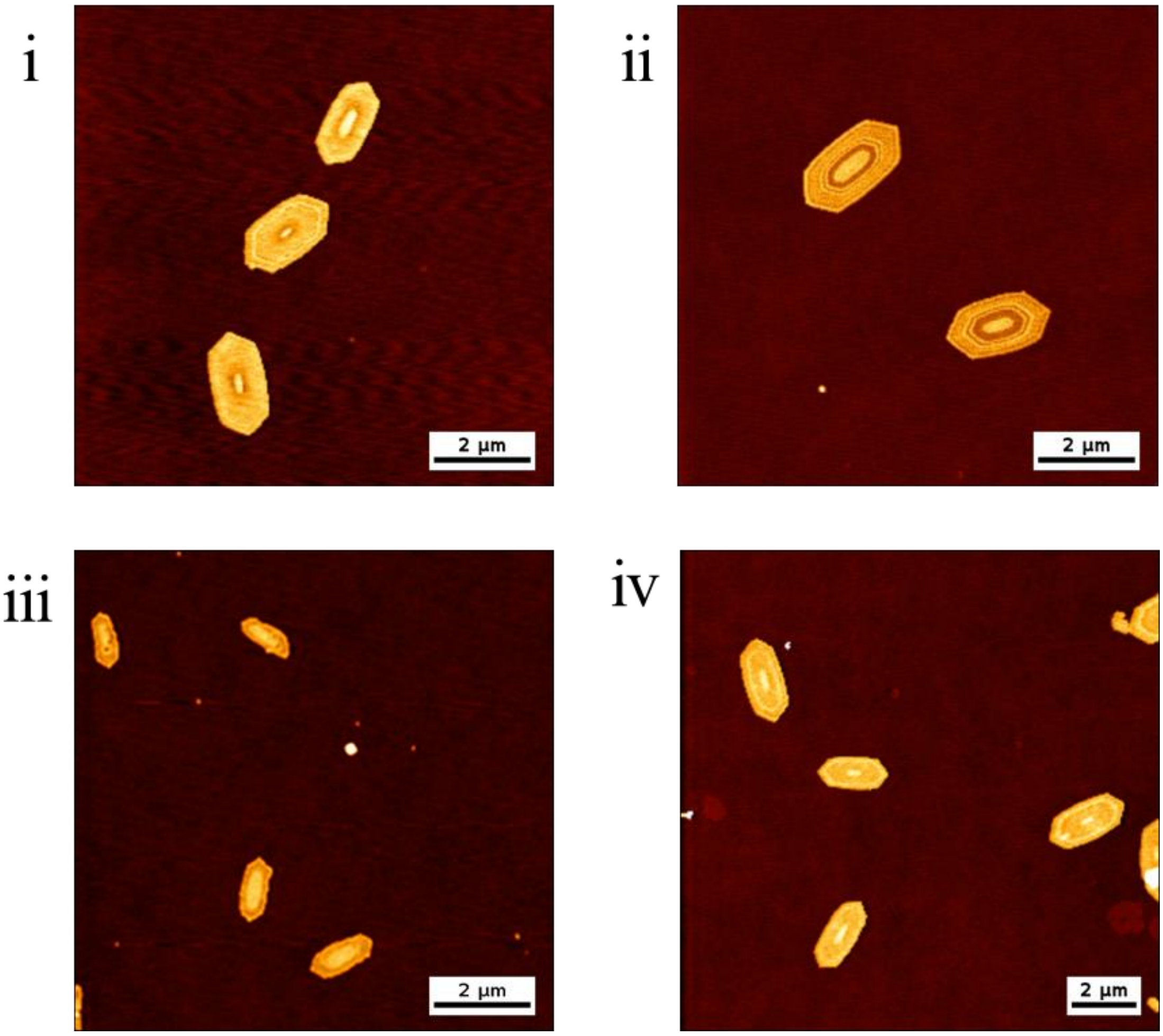

Figure S30. Low-magnification AFM images of a collection of platelets prepared under various programmed temperature and residence time conditions, corresponding to the flow schemes and 2D AFM images shown in Figure 4d.

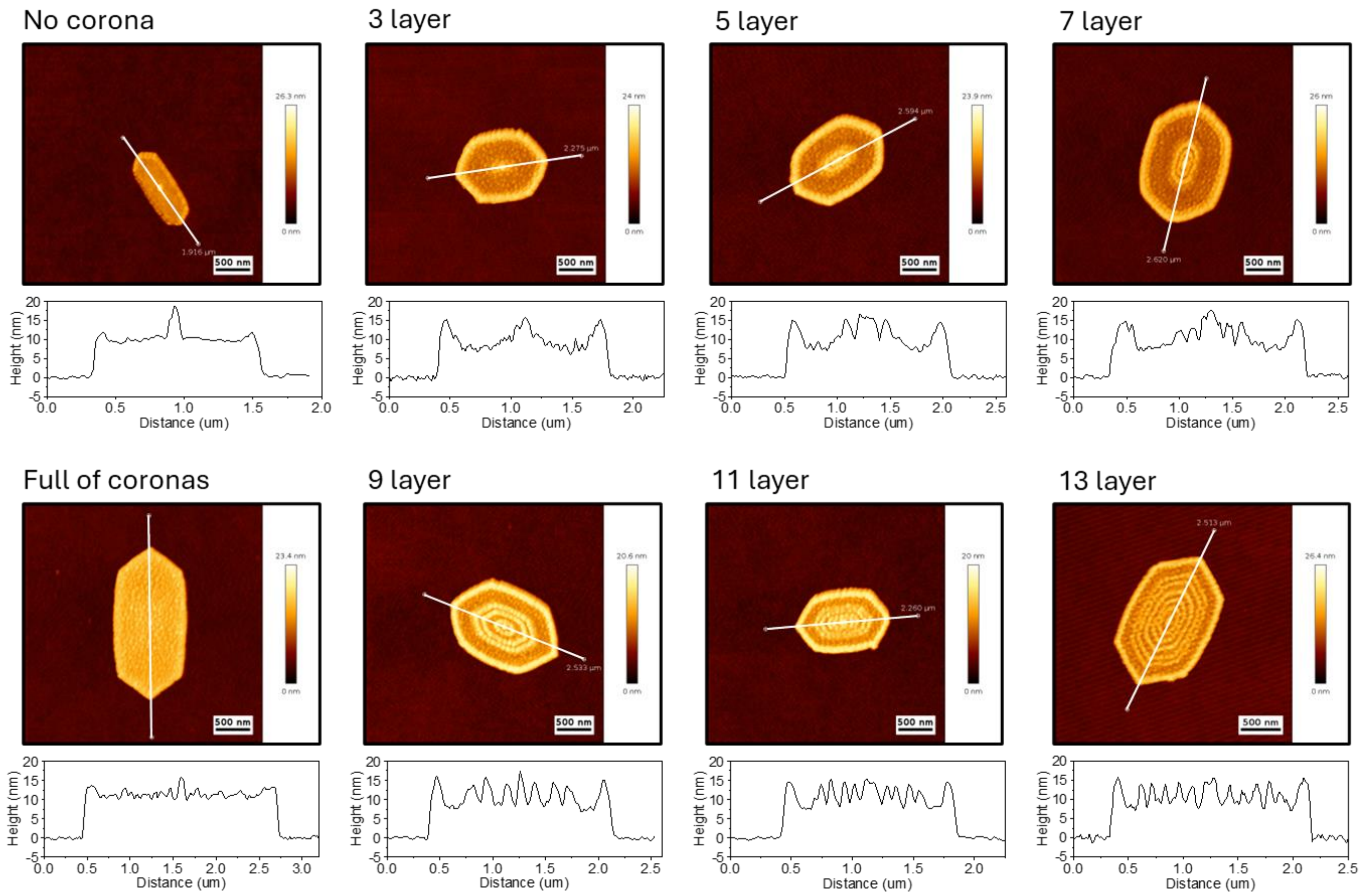

Figure S31. AFM images of platelets with various layered structures prepared in flow (Figure 3a) by changing the number of heating-cooling cycles and subsequently used for QCM tests. Scale bar = 500 nm.

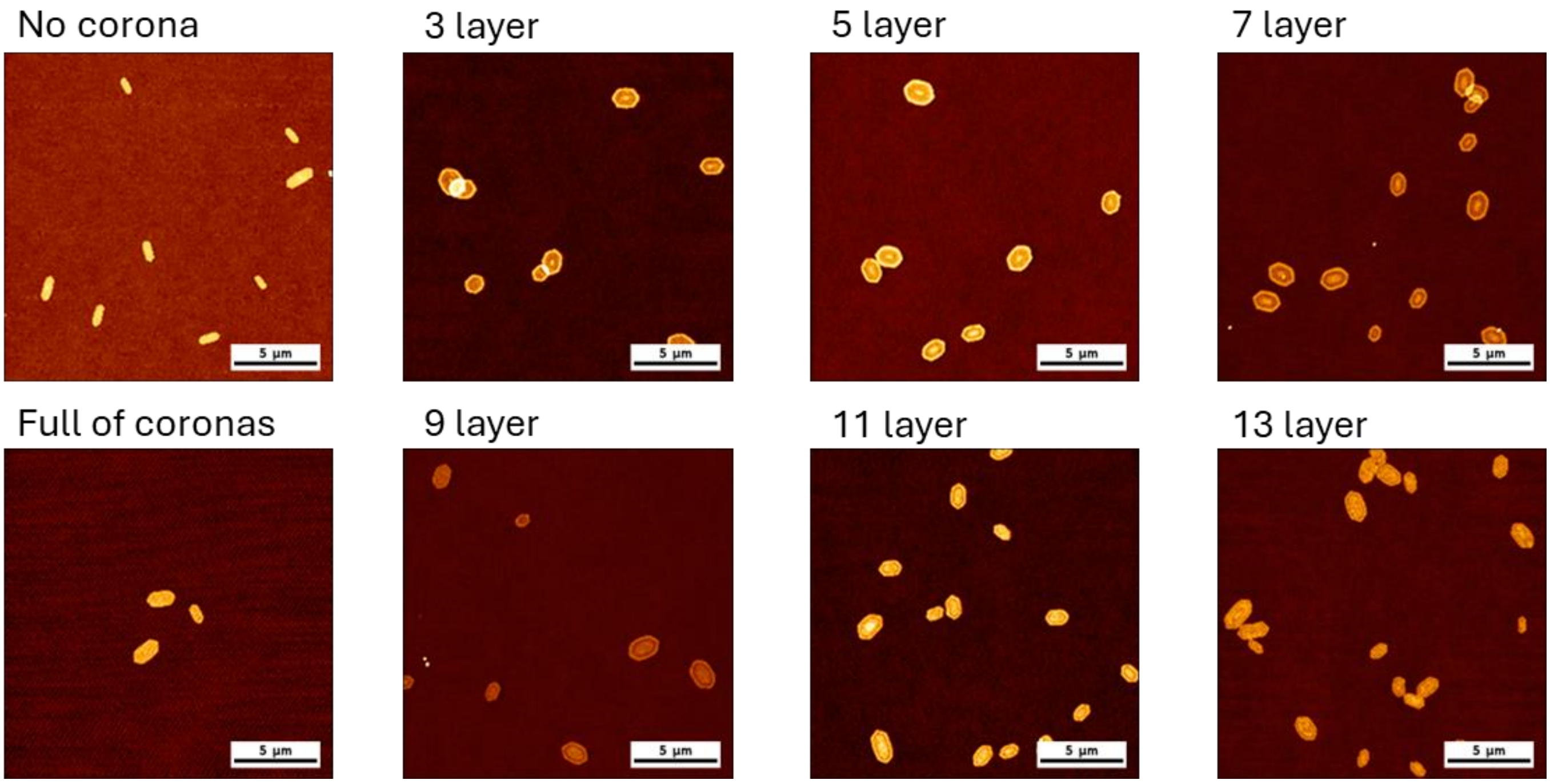

Figure S32. Low-magnification AFM images of a collection of platelets with various layered structures prepared in flow (Figure 3a) by changing the number of heating-cooling cycles and subsequently used for QCM tests.

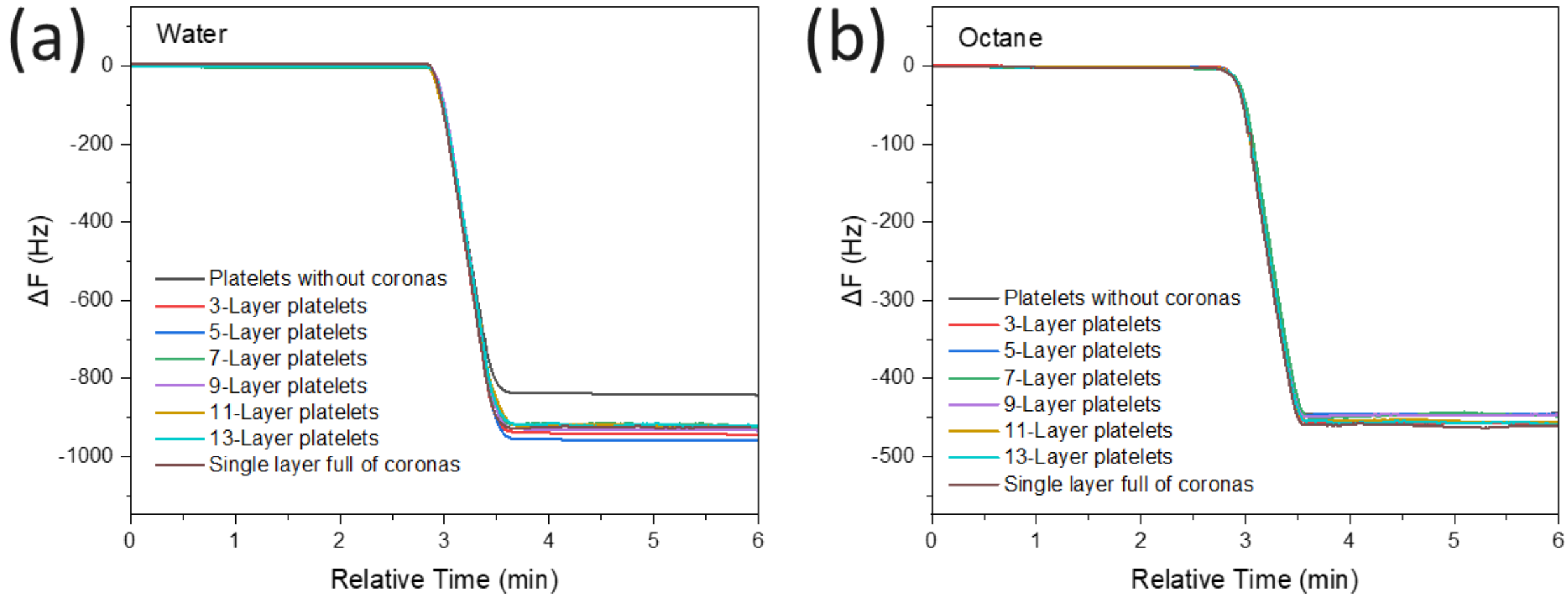

Figure S33. Representative QCM curves under water (a) and octane (b).

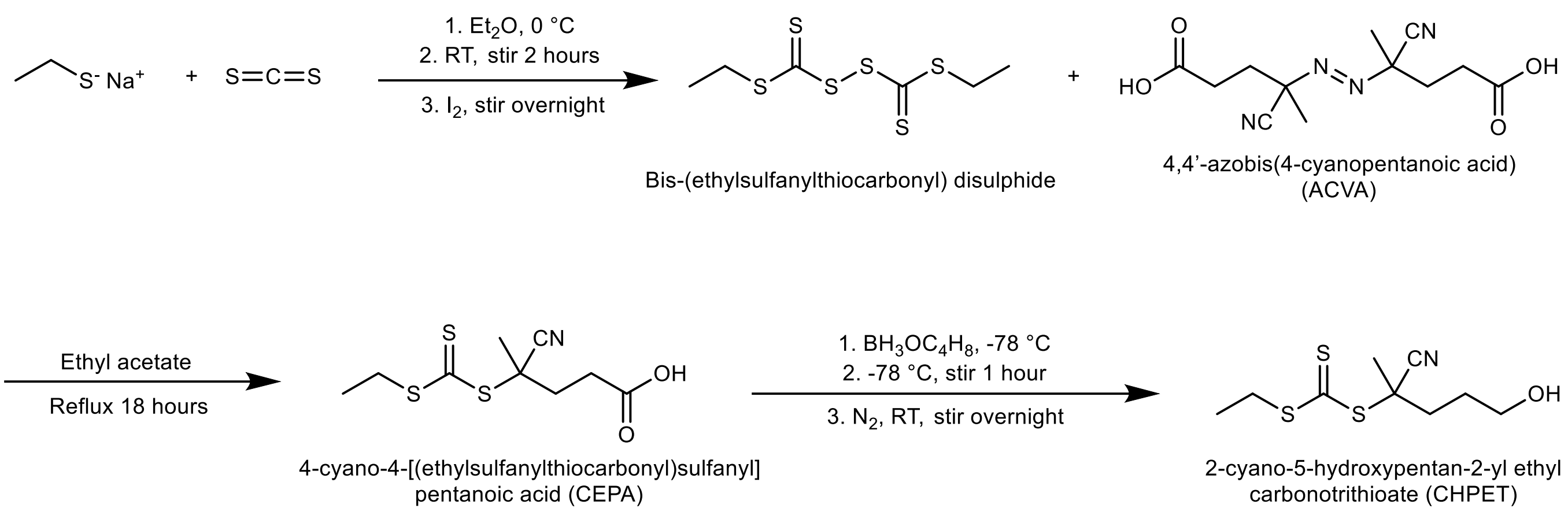

Figure S34. The synthetic route of dual-headed CTA (CHPET).

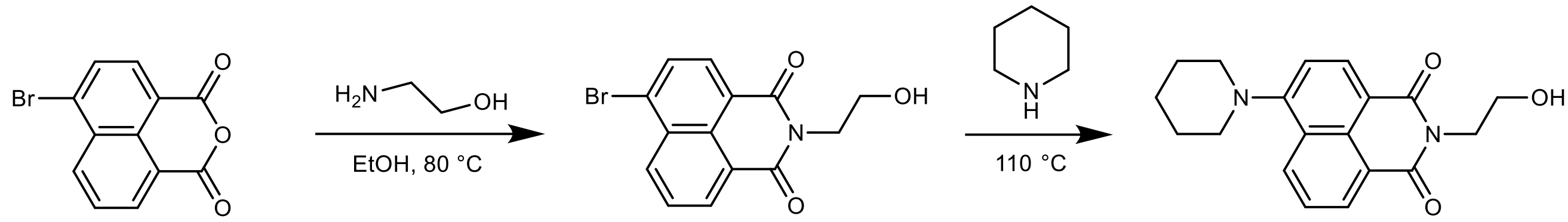

4-bromo-1,8-naphthalic anhydride

Br-NMI-OH

NMI-OH

Figure S35. The synthetic route of NMI-OH.

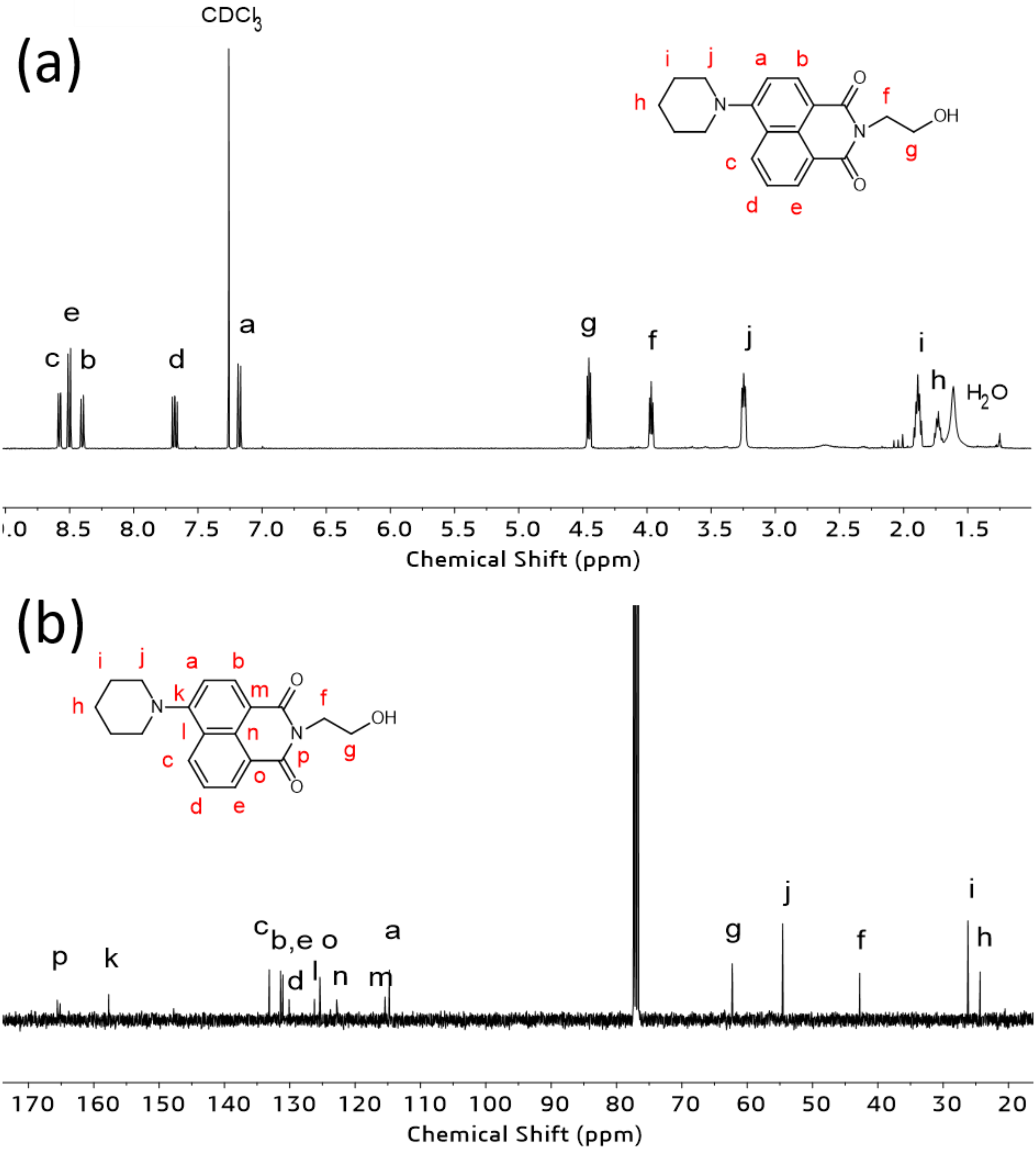

Figure S36. ^{1}H (a) and ^{13}C (b) NMR spectra of NMI-OH (400 MHz; 298 K; in $CDCl_3$).

Supplementary Table

Table S1. SEC results of polymers detected by RI.

Polymer	M_n (kg·mol^{-1})	M_w (kg·mol^{-1})	$Đ$
PCL_{50}*	13.3	14.2	1.06
NMI-PCL_{53}*	13.6	14.5	1.07
PCL_{136}	31.0	32.9	1.06
PCL_{136}-*b*-$PDMA_{200}$	43.8	51.7	1.18

*The preparation of these polymers was reported previously.[1, 3]

Table S2. Size information of platelets (Figure S6) prepared from PCL_{50} / PCL_{136}-*b*-$PDMA_{200}$ unimer at different temperatures.

Temperature	Area (μm^2)			Length (μm)			Width (μm)			L/W
(°C)	A_n	A_w	A_w/A_n	L_n	L_w	L_w/L_n	W_n	W_w	W_w/W_n	
30	0.82	0.82	1.00	1.80	1.80	1.00	0.57	0.57	1.00	3.16
20	0.73	0.97	1.34	1.41	1.56	1.11	0.59	0.66	1.13	2.39
10	0.71	0.95	1.34	1.19	1.28	1.08	0.78	0.84	1.08	1.52

Table S3. Size information of platelets (Figure S7) prepared from PCL_{50} / PCL_{50}-*b*-$PDMA_{200}$ unimer at different temperatures.

Temperature	Area (μm^2)			Length (μm)			Width (μm)			L/W
(°C)	A_n	A_w	A_w/A_n	L_n	L_w	L_w/L_n	W_n	W_w	W_w/W_n	
30	0.50	0.51	1.02	1.35	1.35	1.00	0.47	0.47	1.00	2.89
20	0.49	0.52	1.07	1.14	1.16	1.02	0.54	0.55	1.02	2.10
10	0.70	0.84	1.20	1.10	1.13	1.03	0.90	0.92	1.02	1.22

Table S4. Crystallinity of polymers in solution versus bulk state.

**Temperature (°C)**		**Enthalpy (J/g)**				**Crystallinity* (X_c, %)**			
		**Individual**			**Average+/-SD**	**Individual**			**Average+/-SD**
**PCL_{50}**	30	110.7	112.4	113.4	112.2 ± 1.4	79.3	80.6	81.3	80.4 ± 1.0
	20	113.1	112.2	110.0	111.7 ± 1.6	81.0	80.4	78.8	80.1 ± 1.1
	10	111.7	110.3	110.7	110.9 ± 0.8	80.1	79.0	79.3	79.5 ± 0.5
	bulk	79.4	78.7	78.9	79.0 ± 0.3	56.9	56.4	56.6	56.6 ± 0.3
**PCL_{136}-*b*-$PDMA_{200}$**	30	122.4	125.0	123.6	123.7 ± 1.3	87.8	89.6	88.6	88.7 ± 0.9
	20	133.4	134.7	128.8	132.8 ± 3.1	95.6	96.6	92.3	94.8 ± 2.2
	10	134.2	128.5	127.1	129.9 ± 3.8	96.2	92.1	91.1	93.1 ± 2.7
	bulk	58.3	60.1	60.1	59.5 ± 1.0	41.8	43.1	43.1	42.6 ± 0.7

* The crystallinity (X_c) of PCL was calculated according to $X_c = (\Delta H_m)/(\Delta H_m^0)$, where ΔH_m is the measured mass crystallization/melting enthalpy and ΔH_m^0 is the mass crystallization/melting enthalpy of completely crystalline PCL (139.5 J/g).[4]

Table S5. Size information of platelets (Figure S16) prepared from PCL_{50} / PCL_{136}-*b*-$PDMA_{200}$ unimer of various polymer composition at different temperatures.

Content of Diblock Copolymer (wt%)*	T (°C)	Area (μm^2) A_n	A_w	A_w/A_n	Length (μm) L_n	L_w	L_w/L_n	Width (μm) W_n	W_w	W_w/W_n	L/W
0	30	0.84	0.85	1.01	1.98	1.98	1.00	0.53	0.53	1.00	3.77
20	30	1.06	1.08	1.02	2.26	2.27	1.00	0.59	0.60	1.01	3.80
33	30	1.11	1.11	1.01	2.30	2.31	1.00	0.58	0.58	1.00	3.94
50	30	0.82	0.82	1.00	1.80	1.80	1.00	0.57	0.57	1.00	3.16
66	30	1.02	1.02	1.00	1.93	1.93	1.00	0.64	0.64	1.00	3.04
0	20	0.96	1.11	1.15	1.50	1.56	1.04	0.80	0.83	1.03	1.87
20	20	1.65	2.00	1.21	1.71	1.91	1.12	0.88	0.94	1.07	1.94
33	20	1.34	1.47	1.09	2.03	2.07	1.02	0.80	0.82	1.03	2.54
50	20	0.73	0.97	1.34	1.41	1.56	1.11	0.59	0.66	1.13	2.39
66	20	0.83	0.89	1.07	1.74	1.76	1.01	0.62	0.64	1.03	2.81
0	10	1.02	1.11	1.09	1.37	1.41	1.03	0.97	1.00	1.03	1.41
20	10	0.89	1.24	1.39	1.39	1.51	1.08	0.78	0.85	1.09	1.78
33	10	0.91	1.32	1.45	1.35	1.49	1.10	0.84	0.92	1.09	1.61
50	10	0.71	0.95	1.34	1.19	1.28	1.08	0.78	0.84	1.08	1.52

*Samples with high diblock copolymer content were not included, as the morphology shifted from 2D platelets toward 1D structures, resulting in unusually high length and aspect ratio values.

Table S6. Statistical analysis of height profile.

**Temperature (°C)**	**30**		**20**		**10**	
Diblock content (wt%)	Edge (nm)	Central (nm)	Edge (nm)	Central (nm)	Edge (nm)	Central (nm)
0	10.2 ± 1.1	9.9 ± 0.8	9.6 ± 1.3	9.4 ± 0.9	7.5 ± 0.6	7.9 ± 0.7
20	10.5 ± 1.1	13.7 ± 1.0	10.4 ±1.0	9.4 ± 2.0	12.0 ± 0.7	7.8 ± 0.6
33	10.5 ± 1.6	14.4 ± 1.2	11.8 ± 1.1	10.2 ± 0.8	13.6 ± 0.9	7.9 ± 0.5
50	11.1 ± 1.8	13.5 ± 1.9	12.8 ± 1.0	9.9 ± 0.8	15.5 ± 1.3	7.8 ± 0.7
66	16.7 ± 1.1	15.0 ± 1.1	16.1 ± 0.3	11.2 ± 1.0	16.2 ± 1.1	7.6 ± 0.9
80	17.0 ± 1.4	14.9 ± 0.9	17.1 ± 0.8	11.1 ± 1.2	16.6 ± 0.7	8.6 ± 0.8
100	17.7 ± 0.5	15.8 ± 1.0	17.4 ± 0.3	15.7 ±0.4	17.9 ± 0.4	15.7 ± 0.4

Table S7. Size information of platelets (Figure S19) prepared by temperature-switching in batch.

Sample	Area (μm^2)			Length (μm)			Width (μm)			L/W
	A_n	A_w	A_w/A_n	L_n	L_w	L_w/L_n	W_n	W_w	W_w/W_n	
Batch temperature switching	0.47	0.48	1.01	1.15	1.16	1.01	0.54	0.54	1.00	2.14

Table S8. Flow setup to prepare three-layered platelets.

Sample	Residence Time (s)	
	30 °C	10 °C
(1)	10	50
(2)	30	30
(3)	60	30

Note: Unimer-to-seed ratio was constant at 10, and flow rates of seeds and unimer were maintained at 100 and 1 μL·min $^{-1}$ respectively.

Table S9. Size information of 3-layer platelets (Figure 2) prepared in flow with tunable inner layer size.

Residence	Area (μm^2)			Length (μm)			Width (μm)			L/W
at 30°C (s)	A_n	A_w	A_w/A_n	L_n	L_w	L_w/L_n	W_n	W_w	W_w/W_n	
10	0.70	0.87	1.25	1.31	1.38	1.05	0.69	0.73	1.06	1.91
30	0.55	0.57	1.04	1.18	1.19	1.01	0.61	0.62	1.01	1.93
60	0.65	0.67	1.03	1.12	1.14	1.03	0.67	0.68	1.01	1.66

Table S10. Flow setup to prepare multi layered platelets.

Sample	R_{seed} [a] (μL·min^{-1})	R_{unimer} [a] (μL·min^{-1})	Residence Time (s)												
			30 °C	0 °C	30 °C	0 °C	30 °C	0 °C	30 °C	0 °C	30 °C	0 °C	30 °C	0 °C	Total
13 layer	200	2	10.0	0.4	5.0	0.4	5.0	0.4	5.0	0.4	5.0	0.4	5.0	22.9	60.0
11 layer	150	1.5	13.3	0.6	6.7	0.6	6.7	0.6	6.7	0.6	6.7	0.6	6.7	30.5	80.0
9 layer	100	1	10.0	0.9	10.0	0.9	10.0	0.9	10.0	0.9	10.0	0.9	10.0	55.8	120.0

[a] R_{seed} and R_{unimer} represent the flow rates of seeds and unimer respectively.

Note: Unimer-to-seed ratio was constant at 20.

Table S11. Original epitaxial growth data (Figure 3d) of the 13-layer platelets prepared at 200 μl min^{-1}.

	Heating-cooling cycle	Residence Time (s)	Epitaxial growth increment* (nm)	(nm)	Average increment (nm)	Deviation (nm)
Long axis	1	10.4	-349.4	325.3	337.4	17.0
	2	15.9	-469.9	469.9	469.9	0.0
	3	21.3	-602.4	590.4	596.4	8.5
	4	26.7	-747.0	710.9	728.9	25.5
	5	32.1	-843.3	831.4	837.4	8.5
	6	37.6	-963.8	963.9	963.9	0.0
Short axis	1	10.4	-111.2	98.8	105.0	8.7
	2	15.9	-185.3	185.3	185.3	0.0
	3	21.3	-259.5	259.5	259.5	0.0
	4	26.7	-333.6	333.6	333.6	0.0
	5	32.1	-407.7	395.4	401.5	8.7
	6	37.6	-469.5	469.5	469.5	0.0

*Data was collected in both directions along the axis of platelet, using the center as the origin. Negative (-) values indicate measurements in the opposite direction.

Table S12. Original data for additional kinetic analysis (Case 1, Figure S23c).

	Heating-cooling cycle	Residence Time (s)	Epitaxial growth increment* (nm)	(nm)	Average increment (nm)	Deviation (nm)
Long axis	1	10.4	-111.3	98.2	104.8	9.3
	2	15.9	-229.1	216.0	222.6	9.3
	3	21.3	-346.9	346.9	346.9	0.0
	4	26.7	-451.6	477.8	464.7	18.5
	5	32.1	-582.6	582.5	582.6	0.1
	6	37.6	-700.4	713.5	707.0	9.3
Short axis	1	10.4	-46.3	33.6	40.0	9.0
	2	15.9	-126.2	126.8	126.5	0.4
	3	21.3	-179.5	180.2	179.9	0.5
	4	26.7	-259.4	260.0	259.7	0.4
	5	32.1	-326.0	326.4	326.2	0.3
	6	37.6	-392.6	393.2	392.9	0.4

*Data was collected in both directions along the long axis of platelet, using the center as the origin. Negative (-) values indicate measurements in the opposite direction.

Table S13. Original data for additional kinetic analysis (Case 2, Figure S24c).

	Heating-cooling cycle	Residence Time (s)	Epitaxial growth increment* (nm)	(nm)	Average increment (nm)	Deviation (nm)
Long axis	1	10.4	-169.5	179.2	174.4	6.9
	2	15.9	-285.4	298.6	292.0	9.3
	3	21.3	-391.6	404.8	398.2	9.3
	4	26.7	-537.6	550.8	544.2	9.3
	5	32.1	-657.0	657.0	657.0	0.0
	6	37.6	-763.2	776.4	769.8	9.3
Short axis	1	10.4	-47.8	61.5	54.7	9.7
	2	15.9	-129.8	116.2	123.0	9.6
	3	21.3	-198.1	170.8	184.5	19.3
	4	26.7	-266.4	252.8	259.6	9.6
	5	32.1	-334.7	334.8	334.8	0.1
	6	37.6	-403.0	403.1	403.1	0.1

*Data was collected in both directions along the long axis of platelet, using the center as the origin. Negative (-) values indicate measurements in the opposite direction.

Table S14. Original epitaxial growth data (Figure S25c) of the 11-layer platelets prepared at 150 µl min^{-1}.

	Heating-cooling Cycle	Residence Time (s)	Epitaxial growth increment* (nm)	(nm)	Average increment (nm)	Deviation (nm)
Long axis	1	13.9	-323.2	311.2	317.2	8.5
	2	21.1	-490.8	490.8	490.8	0.0
	3	28.4	-682.3	670.3	676.3	8.5
	4	35.6	-802.0	790.0	796.0	8.5
	5	42.8	-993.5	981.6	987.6	8.5
Short axis	1	13.9	-125.1	137.0	131.0	8.4
	2	21.1	-220.3	220.3	220.3	0.0
	3	28.4	-291.8	315.6	303.7	16.8
	4	35.6	-375.2	399.0	387.1	16.8
	5	42.8	-482.4	494.2	488.3	8.4

*Data was collected in both directions along the long axis of platelet, using the center as the origin. Negative (-) values indicate measurements in the opposite direction.

Table S15. Original epitaxial growth data (Figure S26c) of the 9-layer platelets prepared at 100 µl min^{-1}.

	Heating-cooling cycle	Residence Time (s)	Epitaxial growth increment* (nm)	(nm)	Average increment (nm)	Deviation (nm)
Long axis	1	10.9	-219.9	205.7	212.8	10.0
	2	21.7	-574.6	560.4	567.5	10.0
	3	32.6	-844.2	830.0	837.1	10.0
	4	43.4	-1000.2	986.1	993.2	10.0
Short Axis	1	10.9	-100.5	100.5	100.5	0.0
	2	21.7	-272.7	301.4	287.1	20.3
	3	32.6	-401.9	430.6	416.3	20.3
	4	43.4	-545.5	516.8	531.1	20.3

*Data was collected in both directions along the long axis of platelet, using the center as the origin.

Negative (-) values indicate measurements in the opposite direction.

Table S16. Flow setup to encode growth histories in multi layered platelets.

Sample*	Residence Time (s)						
1	30 °C	0 °C	30 °C	0 °C	30 °C	20 °C	0 °C
	10	0.85	10	0.85	10	10	78.3
2	30 °C	0 °C	20 °C	30 °C	0 °C		
	20	0.85	10	10	79.15		
3	30 °C	0 °C	30 °C	0 °C	30 °C	20 °C	0 °C
	20	1.70	10	0.85	10	10	67.45
4	30 °C	0 °C	30 °C	20 °C	0 °C		
	40	0.85	10	10	59.15		
5	30 °C	20 °C	30 °C	0 °C			
	20	10	10	80			

*Sample 1 is shown in Figure 4b, while Samples 2–5 correspond to the images in Figure 4d (i–iv), respectively.

Note: Unimer-to-seed ratio was constant at 20, and flow rates of seeds and unimer were maintained at 100 and 1 $\mu L \cdot min^{-1}$ respectively.

Table S17. Size information of platelets (Figure 4d) prepared under various programmed temperature and residence time conditions.

Sample	Area (μm^2)			Length (μm)			Width (μm)			L/W
	A_n	A_w	A_w/A_n	L_n	L_w	L_w/L_n	W_n	W_w	W_w/W_n	
i	1.68	1.79	1.07	2.03	2.07	1.02	1.06	1.08	1.02	1.91
ii	1.54	1.75	1.14	1.89	1.96	1.04	1.05	1.09	1.04	1.80
iii	1.12	1.25	1.11	1.70	1.75	1.03	0.85	0.87	1.03	2.00
iv	1.96	2.09	1.06	2.31	2.34	1.02	1.11	1.13	1.01	2.07

Table S18. Flow setup to prepare multi layered platelets for QCM tests.

Sample	Residence Time (s)											
	30 °C	0 °C	30 °C	0 °C	30 °C	0 °C	30 °C	0 °C	30 °C	0 °C	30 °C	0 °C
13-layer	10.0	0.4	5.0	0.4	5.0	0.4	5.0	0.4	5.0	0.4	5.0	22.9
11-layer	10.0	0.4	5.0	0.4	5.0	0.4	5.0	0.4	5.0	28.3	--	--
9-layer	10.0	0.4	5.0	0.4	5.0	0.4	5.0	33.7	--	--	--	--
7-layer	10.0	0.4	5.0	0.4	5.0	39.1	--	--	--	--	--	--
5-layer	10.0	0.4	5.0	44.5	--	--	--	--	--	--	--	--
3-layer	10.0	50.0	--	--	--	--	--	--	--	--	--	--
No corona	120	--	--	--	--	--	--	--	--	--	--	--
	20 °C											
Single layer full of coronas	120	--	--	--	--	--	--	--	--	--	--	--

[a] R_{seed} and R_{unimer} represent the flow rates of seeds and unimer respectively.

Note: Unimer composition of PCL_{50}/PCL_{136}-*b*-$PDMA_{200}$ (1:1 wt%, 5 mg mL^{-1} in $CHCl_3$) was applied except for platelet sample without corona (PCL_{50} only, 5 mg mL^{-1} in $CHCl_3$). Unimer-to-seed ratio was constant at 20, and flow rates of seeds and unimer were maintained at 200 and 2 μL·min $^{-1}$ respectively.

Table S19. Size information of platelets (Figure S32) with various layered structures prepared in flow by changing the number of heating-cooling cycles and subsequently used for QCM tests..

Layer number	Area (μm^2)			Length (μm)			Width (μm)			L/W
	A_n	A_w	A_w/A_n	L_n	L_w	L_w/L_n	W_n	W_w	W_w/W_n	
3	1.37	1.51	1.10	1.52	1.56	1.03	1.17	1.20	1.02	1.29
5	1.39	1.46	1.05	1.59	1.61	1.01	1.16	1.17	1.01	1.38
7	1.32	1.45	1.10	1.56	1.60	1.03	1.08	1.12	1.03	1.44
9	1.41	1.58	1.12	1.64	1.70	1.03	1.13	1.15	1.02	1.46
11	1.09	1.18	1.09	1.48	1.51	1.02	0.96	0.98	1.02	1.53
13	1.47	1.67	1.14	1.76	1.83	1.04	1.06	1.09	1.03	1.66
1 (full of coronas)	0.81	0.95	1.17	1.35	1.41	1.04	0.75	0.78	1.04	1.80
1 (no corona)	0.58	0.65	1.12	1.30	1.35	1.04	0.59	0.60	1.02	2.22

Table S20. Comparison of QCM frequency changes upon transition from air to water.

Sample	Single layer full of coronas					Average	Deviation
Air	5001766	5001781	5001841	5001816	5001807		
Water	5000835	5000834	5000950	5000863	5000845		
Δ (Hz)	-931	-947	-891	-953	-962	-936.8	28.0
Sample	3-layer platelets					Average	Deviation
Air	5003733	5003719	5003733	5003765	5003752		
Water	5002759	5002760	5002780	5002820	5002841		
Δ (Hz)	-974	-959	-953	-945	-911	-948.4	23.4
Sample	5-layer platelets					Average	Deviation
Air	5004362	5004359	5004364	5004376	5004338		
Water	5003400	5003402	5003394	5003442	5003418		
Δ (Hz)	-962	-957	-970	-934	-920	-948.6	20.9
Sample	7-layer platelets					Average	Deviation
Air	5002063	5002041	5002038	5002067	5002037		
Water	5001132	5001101	5001116	5001160	5001136		
Δ (Hz)	-931	-940	-922	-907	-901	-920.2	16.2
Sample	9-layer platelets					Average	Deviation
Air	5002699	5002702	5002709	5002706	5002707		
Water	5001749	5001762	5001777	5001779	5001802		
Δ (Hz)	-950	-940	-932	-927	-905	-930.8	16.8
Sample	11-layer platelets					Average	Deviation
Air	5002381	5002366	5002389	5002390	5002370		
Water	5001457	5001411	5001483	5001486	5001450		
Δ (Hz)	-924	-955	-906	-904	-920	-921.8	20.5
Sample	13-layer platelets					Average	Deviation
Air	5001715	5001659	5001717	5001726	5001712		
Water	5000796	5000682	5000810	5000820	5000803		
Δ (Hz)	-919	-977	-907	-906	-909	-923.6	30.3
Sample	Platelets without coronas					Average	Deviation
Air	5003662	5003632	5003647	5003657	5003736		
Water	5002820	5002789	5002838	5002845	5002865		
Δ (Hz)	-842	-843	-809	-812	-871	-835.4	25.6

Table S21. Comparison of QCM frequency changes upon transition from air to octane.

Sample	Single layer full of coronas					Average	Deviation
Air	5001804	5001815	5001775	5001840	5001707		
Octane	5001333	5001357	5001312	5001382	5001250		
Δ (Hz)	-471	-458	-463	-458	-457	-461.4	5.9
Sample	3-layer platelets					Average	Deviation
Air	5003779	5003763	5003766	5003750	5003744		
Octane	5003324	5003339	5003308	5003294	5003282		
Δ (Hz)	-455	-424	-458	-456	-462	-451.0	15.3
Sample	5-layer platelets					Average	Deviation
Air	5004400	5004392	5004384	5004375	5004366		
Octane	5003949	5003953	5003937	5003920	5003921		
Δ (Hz)	-451	-439	-447	-455	-445	-447.4	6.1
Sample	7-layer platelets					Average	Deviation
Air	5002098	5002081	5002071	5002060	5002055		
Octane	5001646	5001631	5001633	5001615	5001614		
Δ (Hz)	-452	-450	-438	-445	-441	-445.2	5.9
Sample	9-layer platelets					Average	Deviation
Air	5002741	5002714	5002734	5002702	5002716		
Octane	5002286	5002274	5002285	5002256	5002275		
Δ (Hz)	-455	-440	-449	-446	-441	-446.2	6.1
Sample	11-layer platelets					Average	Deviation
Air	5002389	5002386	5002392	5002386	5002368		
Octane	5001935	5001929	5001939	5001952	5001911		
Δ (Hz)	-454	-457	-453	-434	-457	-451.0	9.7
Sample	13-layer platelets					Average	Deviation
Air	5001747	5001690	5001699	5001756	5001728		
Octane	5001298	5001227	5001245	5001297	5001274		
Δ (Hz)	-449	-463	-454	-459	-454	-455.8	5.4
Sample	Platelets without coronas					Average	Deviation
Air	5003665	5003654	5003656	5003646	5003635		
Octane	5003215	5003205	5003213	5003204	5003192		
Δ (Hz)	-450	-449	-443	-442	-443	-445.4	3.8

Supplementary References

(1) Xiao, L.; Parkinson, S. J.; Xia, T.; Edge, P.; O'Reilly, R. K. Enhancing the Scalability of Crystallization-Driven Self-Assembly Using Flow Reactors. *ACS Macro Lett.* **2023**, *12*, 1636-1641.

(2) Rajak, A.; Das, A. Crystallization-Driven Controlled Two-Dimensional (2D) Assemblies from Chromophore-Appended Poly(L-lactide)s: Highly Efficient Energy Transfer on a 2D Surface. *Angew. Chem. Int. Ed.* **2022**, *61*, e202116572.

(3) Xiao, L.; Xia, T.; Zhang, J.; Parkinson, S. J.; Rho, J. Y.; Dove, A. P.; O' Reilly, R. K. Direct preparation of two-dimensional platelets from polymers enabled by accelerated seed formation. *Nat. Synth.* **2025**, *4*, 808–815.

(4) Fernández-Tena, A.; Pérez-Camargo, R. A.; Coulembier, O.; Sangroniz, L.; Aranburu, N.; Guerrica-Echevarria, G.; Liu, G.; Wang, D.; Cavallo, D.; Müller, A. J. Effect of Molecular Weight on the Crystallization and Melt Memory of Poly(ε-caprolactone) (PCL). *Macromolecules* **2023**, *56*, 4602-4620.
