# Supplementary material for: Constructing and decoding tree-ring-like surface patterns on self-assembled polymer platelets
Source: Nat Commun. 2026 Aug 20;17:9961. doi: 10.1038/s41467-026-76483-7 (PMC13586395; doi:10.1038/s41467-026-76483-7)
Supplement: Supplementary file 2 [file 41467_2026_76483_MOESM2_ESM.pdf]

nature portfolio

Peer Review File

Constructing and Decoding Tree-Ring-Like Surface Patterns on Self-Assembled Polymer Platelets

Corresponding Author: Professor Rachel O'Reilly

This file contains all reviewer reports in order by version, followed by all author rebuttals in order by version.

Version 0:

Reviewer comments:

Reviewer #1

(Remarks to the Author)
This paper presents a novel, controllable, and scalable strategy for constructing polymer platelets with complex 3D surface patterns. By combining flow reactors with CDSA technology, it demonstrates the ability to encode and decode the growth history of materials at the nanoscale. The research features systematic content, sufficient data, and clear mechanisms, exhibiting strong innovation and broad application prospects. Publishing in Nature Communication is highly recommended. Below are a couple of detailed points for consideration to further improve the manuscript.

1. The QCM results show that an increase in the number of layers leads to a decrease in hydrophilicity, but no quantitative correlation has been established with parameters such as surface roughness or air entrapment. It is recommended to supplement the study with contact angle measurements or a more systematic analysis of surface energy.
2. Why is the degree of polymerization of homopolymer PCL inconsistent with that of the PCL block in PCL-b-PDMA block copolymer?
3. It is necessary for the authors to explain why a cycle period of 5.4 s was chosen.
4. The current study heavily relies on the difference in crystallization rates between the PCL/PDMA system. Whether this strategy is applicable to other polymer systems remains unclear. It is recommended to include a discussion on the applicability to other polymer systems, or to clearly define the scope and limitations of the proposed strategy.
5. It is recommended that the fitting equation in Figure 3d be labeled with the R^2 value.
6. Whether the seeds are affected by shear forces, temperature changes, or dilution effects before entering the flow reactor remains unclear. These factors may influence the uniformity of growth and the homogeneity of the layered structures. It is recommended to perform control experiments comparing the morphology and growth activity of seeds under batch and flow conditions to confirm that the flow conditions do not compromise seed activity.

Reviewer #2

(Remarks to the Author)
This manuscript provides an efficient, easy-handling and scalable strategy to fabricate 2D nanostructures with tunable surface pattern by living crystallization-driven self-assembly from PCL-containing homopolymer/copolymer. What stands out in this study is clever combination of temperature-dependent kinetic difference in crystallization rates of PCL homopolymers and copolymers and the concept of flow reactor. By varying the temperature, a series of interesting nanostructures with layered, concave, and convex features can be generated in a controlled manner within just several minutes. Almost all of formed nanostructures were uniform and had beautiful surface patterns, which can be pre-designed and generated in programmed way. Moreover, the authors also systematically examined the influence of composition/surface-pattern/chemistry on the wettability by QCM, which provides some useful guidelines in the preparation of functional nanomaterials with smart surface. This manuscript is well-structured, rigorously conducted, and clearly presented. It offers new opportunities for programmable materials in nanotechnology and functional systems, making it a substantial addition to the field of nanomaterials. The publication of this manuscript is strongly recommended after addressing following minor comments:
1) It could be true the variations of height of micelles were attributed to difference in the spatial distributions of block copolymers. However, another important factor also should be taken into account---the different levels of folding of PCL chains. Some previous reports showed that crystalline polymers had different levels of chain folding when the crystallization

occurred at the different temperatures. The folding of PCL not only affected the height of micelles, but also degree of crowding of corona, and thus, it should be important factor determining the height of micelles. However, the authors just focused on the contribution from the corona chains. It will be more reasonable if this factor would be considered.
2) The authors only focused on the surface features. More information about the size of resulting micelles should be provided.
3) In most of images, only one or two micelles were provided. How about the efficiency in the seeded growth? Were there present of some imperfect micelles which were not as-designed? Some more information of percentages of perfect and imperfect micelles should be provided.
4) In the whole work, only one homopolymer and one block copolymer were used. It was well-known that the crystallization rate/kinetics was highly dependent on the composition of crystalline polymers/copolymers. Were there any strict requirements for the chain length and composition of homopolymer/copolymer? If it is, some comments on this points should be provided.
5) Just for curiosity, what would happen for this system at lower or higher temperatures? Most readers might ask the same question. It will be much better if additional results could be provided on this point.
6) In figure 1, it appeared that the temperature can affect the aspect ratio of resulting micelles. Interestingly, in figure 3, it appeared that the aspect ratio of resulting micelles almost did not change obtained at different heating-cooling cycles. It will be better if some comments on this point could be provided.

Reviewer #3

(Remarks to the Author)
This highly intriguing paper deals with the preparation of well-defined platelets with a tree-ring-like surface pattern employing co-crystallization-driven self-assembly of poly(ε-caprolactone)-block-poly(N,N-dimethylacrylamide) (PCL-b-PDMA) diblock copolymers with PCL homopolymers. The use of precisely programmed flow reactors allows continuous production of the platelets and an efficient tuning of corona structure by variations in temperature and residence time, whereby temperature is the decisive parameter to control co-assembly over self-sorting due to different crystallization kinetics of the diblock copolymer and homopolymer. This enables a precise encoding of self-assembly history into the corona structure of the platelets, resembling the encoding of growth history (in terms of environmental conditions) observed for trees. Moreover, growth kinetics can be directly extracted from the encoded structures, which is difficult (or impossible) to achieve by “conventional” CDSA methods. The approach presented is highly appealing and there are currently no alternative methods available to achieve tree-ring-like structures with such high precision. Hence, publication in Nat. Commun. is strongly supported after minor revisions as detailed below.

I) Rate constants derived from UV-vis
The authors describe a kinetic model that allows determination of growth constants from UV-vis measurements. The model is sound; however, the evaluation of UV-vis data raises some questions. First, the fits presented in Figure S13 do not always properly describe the experimental data. Especially for the diblock copolymer at 30 °C there is a significant deviation. In addition, the standard deviations for variables a, b, and c are very high, even exceeding the mean in most cases (most pronounced for homopolymer kinetics). The chosen fitting model does also deviate from the kinetic model. For fitting A(t) = a*(1-exp(-b*t^c)) is used, whereby the kinetics model is A(t) = K*(1-exp(-k’*t)) (k’ = k [S]0). According to the model, the extracted apparent rate constant should have the unit [L/(mol*min)] as seed concentration is included in k’; one page 9 1/min is used as unit instead. Hence, it is recommended to discuss the data only qualitatively without giving rate constants. For example, plotting the kinetics for diblock copolymer and homopolymer in one plot for a given temperature would also show that homopolymer crystallization is faster at low temperatures and comparable at high temperatures. Is it possible to use the Nano-DSC to study crystallization kinetics?

II) Readability of Figures
In Figure 1d the axes labels are hard to read. It is recommended to rearrange the Figure.
In Figure 4d it is difficult to distinguish between pale orange and pale red color in flow schemes. It is recommended to either use a different color combination or reduce transparency. In addition, due to the small size it is hard to see differences in the provided height profiles. The Figure might be rearranged to allow larger sized plots or some of the results could be moved to Supporting Information.

III) QCM data
On page 20 the authors state that the hydrophobic nature of PCL renders the surface of the PCL platelets less hydrophilic. This might be kind of misleading, as the surface of neat PCL platelets should be rather hydrophilic due to the presence of polar RAFT groups and OH-end groups at the fold surface. This is also supported by the rather small difference in frequency shift between platelets with and without corona. Nevertheless, the neat PCL platelets are less hydrophilic with respect to the layered platelets with hydrophilic PDMA domains. The measured differences between platelets with varying layer numbers are comparable to the standard deviations. Hence, it is difficult to draw a conclusion on the dependence on layer number and only the difference between neat PCL and layered platelets might be discussed. Measuring the interfacial tension might be a more sensitive method to probe the influence of corona pattern.

IV) Minor comments on manuscript
- A recent report on the preparation of platelets with stripe-like surface patterns by co-crystallization-driven self-assembly might be added to the Introduction, which also allows to “write” defined corona patterns in a one-step procedure (Macromolecules 2026, 59, 3092).
- The commonly used term “diblock polymer” might be replaced by “diblock copolymer”

- Polycaprolactone should be replaced by poly(ε-caprolactone).
- Page 6: it is recommended to add a short note that the used seeds were dispersed in ethanol at c = 0.01 mg/mL, otherwise the procedure might not be clear to the reader.
- Figures 2 - 4: please specify the diblock copolymer content in the unimer mixture, employed unimer-to-seed ratio, and flow rate for means of clarity. In addition, a note might be added on page 12 that details on employed flow rates and concentrations can be found in Supporting Information.
- Can the authors comment on why for the growth kinetics (e.g. Fig. S22, S23) negative y-axis intercepts were found. In principle, one might expect that dimensions of seeds (ca. 25 nm) define the intercept.

V) Supporting information
- Figure S6, S15, S19: height profiles are hard to read due to limited resolution and font size.
- Figure S5, S16: it is recommended to provide z-scales.
- Table S6-S10: it is recommended to give only one digit for time and increment.
- Figure S22a-S25a: it is recommended to increase the size of the AFM images, as details are difficult to recognize.
- Figure 24c, 25c: here, growth time t is used for fitting the data; however, in all other related Figures the cycle number x is used. It is recommended to uniformly use the cycle number x for fitting to allow for comparison.
- Figure S27: the standard deviations given in the Table are very high, maybe there is a problem with units? Please replace "layer thickness" by "aspect ratio".
- Figure S28: height profiles might be included for better visualization of layer numbers.
- Please check SI for typos and correct wording. For example: last sentence on page S3 seems to be incomplete, polydimethylacetamide should read poly(N,N-dimethylacrylamide) on page S6, "normalized size exclusion chromatograms" should be replaced by "apparent molar mass distribution" on page S12 and S18, "for stain" might be replaced by "as staining agent" on page S14, please use uniform notation for diblock copolymer in Figure S4 and S5 (PCL_{50}-*b*-$PDMA_{196}$),…

Version 1:

Reviewer comments:

Reviewer #1

(Remarks to the Author)
In the revised manuscript, the authors have properly addressed all my previous concerns. I am happy to recommend the publicaiton of the manuscript in Nature Communications.

Reviewer #2

(Remarks to the Author)
It is fair to say that the authors have done a nice job on the revision. The points raised by the reviewers have been properly addressed. It appears that the manuscript is suitable for the publication as it is.

Reviewer #3

(Remarks to the Author)
The authors have very convincingly addressed all points raised by the reviewers.
This is a very sound and intriguing work, presenting a central and highly relevant contribution to the field, paving the way to the production of well-defined 2D assemblies with tailored surface patterns. Hence, publication of the manuscript in Nat. Commun in its current version is strongly supported.

Open Access This Peer Review File is licensed under a Creative Commons Attribution 4.0 International License, which permits use, sharing, adaptation, distribution and reproduction in any medium or format, as long as you give appropriate credit to the original author(s) and the source, provide a link to the Creative Commons license, and indicate if changes were made.
In cases where reviewers are anonymous, credit should be given to 'Anonymous Referee' and the source.
The images or other third party material in this Peer Review File are included in the article's Creative Commons license, unless indicated otherwise in a credit line to the material. If material is not included in the article's Creative Commons license and your intended use is not permitted by statutory regulation or exceeds the permitted use, you will need to obtain permission directly from the copyright holder.
To view a copy of this license, visit https://creativecommons.org/licenses/by/4.0/

Responses to the Reviewers' Reports：Constructing and Decoding Tree-Ring-Like Surface Patterns on Self-Assembled Polymer Platelets

We thank the reviewers for their constructive and insightful comments, which we have addressed in detail in this point-by-point response. The corresponding changes to the manuscript and supporting information were highlighted in yellow.

Reviewer #1

This paper presents a novel, controllable, and scalable strategy for constructing polymer platelets with complex 3D surface patterns. By combining flow reactors with CDSA technology, it demonstrates the ability to encode and decode the growth history of materials at the nanoscale. The research features systematic content, sufficient data, and clear mechanisms, exhibiting strong innovation and broad application prospects. Publishing in Nature Communication is highly recommended. Below are a couple of detailed points for consideration to further improve the manuscript.

1. The QCM results show that an increase in the number of layers leads to a decrease in hydrophilicity, but no quantitative correlation has been established with parameters such as surface roughness or air entrapment. It is recommended to supplement the study with contact angle measurements or a more systematic analysis of surface energy.

Response: We thank the reviewer for this constructive suggestion. We agree that contact angle measurements are commonly used to evaluate surface hydrophilicity and were initially considered in this study. However, during preliminary experiments, we found that the contact angle results were not sufficiently reproducible. This is likely due to the hierarchical nature of our system: in addition to the intrinsic surface patterns on the platelet, the macroscopic wettability is strongly influenced by factors under mesoscale, such as platelet distribution, surface coverage, and degree of alignment on the test matrix (Science 318, 1618-1622 (2007); Current Opinion in Colloid & Interface Science 19, 355-367 (2014)). These structural variations can lead to significant changes in the apparent contact angle, thereby introducing

considerable measurement variability. For this reason, we chose to employ QCM measurements, which are more sensitive to interfacial interactions at the molecular level and less affected by mesoscale platelet arrangement. Importantly, the QCM results showed good reproducibility across repeated experiments and provided a more consistent basis for comparing samples with different layer numbers. We acknowledge that a fully quantitative correlation between hydrophilicity, surface roughness, and air entrapment remains an interesting topic for further investigation, and we will consider incorporating more systematic surface energy analysis in future studies.

2. Why is the degree of polymerization of homopolymer PCL inconsistent with that of the PCL block in PCL-b-PDMA block copolymer?

Response: We thank the reviewer for raising this important point. In this study, the degree of polymerization (DP) of the PCL block in the PCL-*b*-PDMA copolymer was intentionally increased to achieve a crystallization capacity comparable to that of the PCL_{50} homopolymer. This design allows external stimuli, such as temperature, to be used to tune the relative crystallization rates of the two polymers. As demonstrated in our previous work and shown in our control (Figure S6), PCL_{50} homopolymer exhibits significantly faster crystallization kinetics than the PCL_{50}-*b*-$PDMA_{200}$ block copolymer with an equivalent DP of PCL block, regardless of temperature variation. Therefore, simply matching the DP does not lead to temperature-responsive growing behavior.

To clarify this rationale, we have added the following explanation on **Page 6, Line 9-12** in the revised manuscript.

In contrast to our previous work,[12, 14, 18, 26, 29] a diblock copolymer (PCL_{136}-*b*-$PDMA_{200}$) with a much longer PCL block was prepared to increase the crystallization capacity to a level comparable to that of the PCL_{50} homopolymer, thereby enabling external stimuli to modulate their relative crystallization rates.

3. It is necessary for the authors to explain why a cycle period of 5.4 s was chosen.

Response: We thank the reviewer for raising this point. The cycle period of 5.4 s corresponds to the residence time of a single heating-cooling cycle during living growth in flow at a flow rate of 200 μL min^{-1}. Accordingly, cycle periods of 7.2 s and 10.9 s were used to study the cases at flow rates of 150 and 100 μL min^{-1}, respectively, based on their corresponding residence times.

We have added the following explanation to clarify this point on **Page 16, Line 20-23** in the revised manuscript.

Using the cycle period (5.4 s), defined as the residence time for one complete heating-cooling cycle at a flow rate of 200 μL min^{-1}, we calculated growth rates of 123 nm per cycle (22.8 nm/s) along the long axis and 73 nm per cycle (13.4 nm/s) along the short axis.

4. The current study heavily relies on the difference in crystallization rates between the PCL/PDMA system. Whether this strategy is applicable to other polymer systems remains unclear. It is recommended to include a discussion on the applicability to other polymer systems, or to clearly define the scope and limitations of the proposed strategy.

Response: We thank the reviewer for this insightful comment. The following paragraph was added on **Page 20, Line 10-18** in the revised manuscript.

The key concept of this study is the temperature-dependent tuning of the relative crystallization rates of PCL-based homopolymer and block copolymer, coupled with flow processing to precisely control the thermal history, thereby enabling programmable nanoparticle morphologies during growth. In principle, this strategy can be extended to other CDSA systems comprising multiple components, provided that their self-assembly kinetics are sufficiently distinct yet responsive to external stimuli, allowing modulation of their relative rates. Such behavior may be achieved through polymer design or by tuning assembly conditions. More broadly, this flow-enabled approach offers a framework for achieving precise control over multicomponent self-assembly processes governed by nucleation-growth mechanisms.

5. It is recommended that the fitting equation in Figure 3d be labeled with the R^2 value.

Response: We thank the reviewer for this helpful suggestion. The fitting equation in Figure 3d has been updated to include the corresponding R^2 value in the revised manuscript. In addition, R^2 values have also been included for Figures S22-S25.

6. Whether the seeds are affected by shear forces, temperature changes, or dilution effects before entering the flow reactor remains unclear. These factors may influence the uniformity of growth and the homogeneity of the layered structures. It is recommended to perform control experiments comparing the morphology and

growth activity of seeds under batch and flow conditions to confirm that the flow conditions do not compromise seed activity.

Response: We thank the reviewer for raising this point. The influence of key parameters, including temperature and shear force (flow rate), has been systematically investigated in our previous work (ACS Macro Lett. 2023, 12, 12, 1636–1641.). These studies showed that shear force in flow has no significant effect on the size or uniformity of the resulting platelets. In contrast, temperature plays a more critical role, as higher temperatures enhance mass transport under flow conditions, leading to improved growth uniformity. Consistent trends are also observed in the present study (Figures S5 and S15). Accordingly, all flow living CDSA experiments were initiated at 30 °C to ensure efficient mixing of seeds and unimer prior to switching to other temperatures.

Regarding dilution, our previous results indicate that seed activity and the morphology of the resulting platelets are not significantly affected (Adv. Mater.2024, 36, 2308154.; Macromolecules 2024, 57, 23, 11210–11220.). To further confirm this, we performed living CDSA using more diluted seeds (0.0025 mg mL^{-1}) under both batch and flow conditions (Figure S19). In all cases, the resulting platelets exhibited comparable morphology and size distributions. DLS analysis showed similar particle sizes without detectable low-size populations corresponding to residual seeds, indicating uniform seed activity and complete conversion to platelets.

The following sentences have been added to the revised manuscript.

Page 12, Line 22-25

As our previous results show no significant differences between living CDSA performed under batch and flow conditions,[26] and dilution does not markedly affect seed activity (Figure S19),[14] the flow setup and procedure used for living CDSA in this study was adapted from our earlier work.

Page 19, Line 2-5

As previous studies have shown that elevated temperatures facilitate mass diffusion and improve platelet uniformity,[26] all flow schemes were initiated at 30 °C, with a relatively larger proportion of the total residence time maintained at this temperature.

Figure S19 in Supporting Information

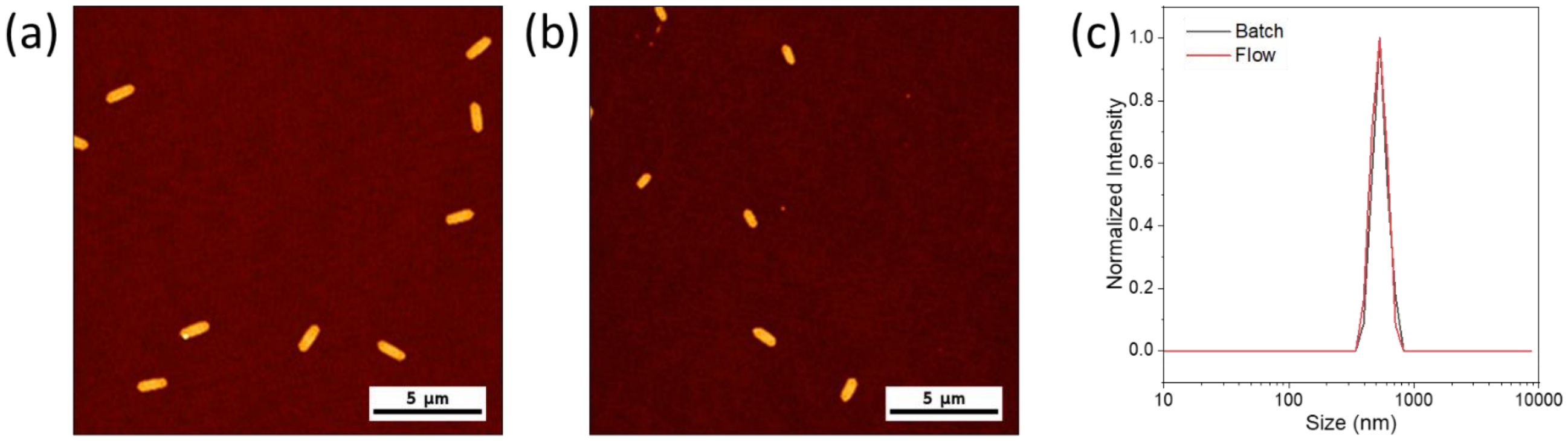

Figure S19. Comparison of platelet samples prepared from diluted seeds (0.0025 mg mL^{-1}) and unimer (1:1 wt % PCL_{50} / PCL_{136}-*b*-$PDMA_{200}$; 5 mg mL^{-1} in $CHCl_3$; unimer-to-seed ratio = 10) under batch and flow conditions: (a) AFM image of platelets prepared in batch, (b) AFM image of platelets prepared in flow, and (c) DLS size distributions of the resulting platelets based on scattering intensity.

Reviewer #2

This manuscript provides an efficient, easy-handling and scalable strategy to fabricate 2D nanostructures with tunable surface pattern by living crystallization-driven self-assembly from PCL-containing homopolymer/copolymer. What stands out in this study is clever combination of temperature-dependent kinetic difference in crystallization rates of PCL homopolymers and copolymers and the concept of flow reactor. By varying the temperature, a series of interesting nanostructures with layered, concave, and convex features can be generated in a controlled manner within just several minutes. Almost all of formed nanostructures were uniform and had beautiful surface patterns, which can be pre-designed and generated in programmed way. Moreover, the authors also systematically examined the influence of composition/surface-pattern/chemistry on the wettability by QCM, which provides some useful guidelines in the preparation of functional nanomaterials with smart surface. This manuscript is well-structured, rigorously conducted, and clearly presented. It offers new opportunities for programmable materials in nanotechnology and functional systems, making it a substantial addition to the field of nanomaterials. The publication of this manuscript is strongly recommended after addressing following minor comments:

1) It could be true the variations of height of micelles were attributed to difference in the spatial distributions of block copolymers. However, another important factor also should be taken into account---the different levels of folding of PCL chains. Some previous reports showed that crystalline polymers had different levels of chain folding when the crystallization occurred at the different temperatures. The folding of PCL not only affected the height of micelles, but also degree of crowding of corona, and thus, it should be important factor determining the height of micelles. However, the authors just focused on the contribution from the corona chains. It will be more reasonable if this factor would be considered.

Response: We thank the reviewer for this insightful comment. We agree that crystallization temperature can influence the folding of PCL chains, leading to variations in the thickness of the crystalline core. Consistent with previous reports (*Nat Commun* 7, 11119 (2016)), we also observe that higher temperatures result in thicker platelets when using PCL homopolymer alone (Figure S17e, Table S6), confirming that chain folding contributes to thickness variations.

However, this effect does not alter our main conclusion. In batch experiments where platelet samples were prepared at a fixed temperature, the PCL core thickness within a given sample is expected to be relatively uniform, and it is therefore reasonable to attribute the observed extra height variations primarily to differences in corona distribution. This also applies to experiments in which the homopolymer-to-block copolymer ratio was varied at a fixed temperature (Figure S17a-c), where systematic height differences between the platelet center and edge are observed due to the corona distribution as core thickness is constant at the fixed temperature.

Regarding the effect of corona crowding arising from differences in PCL chain folding, our results (Figure S17e) show that nanostructures formed solely from the diblock copolymer exhibit similar heights even when prepared at different temperatures. This observation suggests that variations in corona crowding induced by differences in PCL folding do not have a significant effect on the overall platelet height.

We acknowledge that PCL chain folding may also contribute to height variations under temperature-switching conditions in flow. However, its contribution appears to be comparatively minor. Specifically, the maximum thickness variation associated with PCL folding is ~2 nm (as observed for living growth using homopolymer only, Figure S17e), whereas the overall height variation induced by temperature switching reaches ~8-10 nm (e.g. Figure 2c). This significant difference indicates that changes in block copolymer distribution are the dominant factor governing the observed height variations.

To indicate this point, the following content was updated on **Page 13, Line 1-10** in the revised manuscript:

The high surface area-to-volume ratio of the flow reactor facilitated rapid heat transfer,[44-46] allowing precise temperature control that modulated the self-assembly behavior from co-assembly to self-sorting. Under these flow conditions, uniform three-layer platelets were consistently formed within 2 min (Figure 2b and Table S9). Notably, a significant height variation (~9 nm) was observed between the layers, which we attribute to differences in corona distribution arising from the temperature-dependent self-assembly behavior. Although temperature can also influence the thickness of the PCL crystalline core, the variation observed is limited (~2 nm, Figure S17e and Table S3), indicating that the observed height differences primarily arise from changes in block copolymer distribution rather than crystalline core thickness.

2) The authors only focused on the surface features. More information about the size of resulting micelles should be provided.

Response: We thank the reviewer for this comment. Indeed, the primary focus of this work is the regulation and encoding of surface topographical features. Nevertheless, we agree that size information is also valuable for evaluating the resulting assemblies. Therefore, additional statistical size information shown in **Tables S2, S3, S5, S7, S9, S17, and S19**, including platelet area, length, width, and aspect ratio, has been added to the revised Supporting Information.

3) In most of images, only one or two micelles were provided. How about the efficiency in the seeded growth? Were there present of some imperfect micelles which were not as-designed? Some more information of percentages of perfect and imperfect micelles should be provided.

Response: We thank the reviewer for this important comment. In practice, we investigated more flow schemes than those reported in the manuscript, and the examples presented here were selected as representative samples that reproducibly matched the intended designs. We did observe some imperfect micelles in certain experiments, with the primary issue being the absence of one or more designed layers. Based on our observations, we reason that the platelet dimensions are limited during the growth process, and therefore the size of each individual layer must be carefully controlled to ensure that all designed layers can be successfully expressed within a single platelet. For example, when one layer grows disproportionately large and occupies a substantial fraction of the platelet area, the available space for subsequent growth becomes more limited, which can hinder the clear formation and resolution of the remaining designed layers within the final platelet.

In addition, we found that consideration of the residence time distribution is critical for achieving well-defined structures. Specifically, longer residence times at 30 °C, particularly during the initial stages of growth, resulted in more uniform platelets. This observation is consistent with our previous findings that higher temperatures facilitate more efficient mixing between seeds and unimer. Consequently, all flow schemes reported in this work were initiated at 30 °C and designed to maintain a relatively larger proportion of the residence time at this temperature.

As the system involves complex self-assembly behavior and structural distributions, precise statistical quantification of “perfect” versus “imperfect” micelles remains challenging. Nevertheless, the reported conditions reproducibly generated the targeted structures as the dominant morphology observed by AFM.

In addition, we have included AFM images containing a larger number of platelets in the revised Supporting Information.

The following sentence was added on **Page 19, Line 2-5** in the revised manuscript for further clarification.

As previous studies have shown that elevated temperatures facilitate mass diffusion and improve platelet uniformity,[26] all flow schemes were initiated at 30 °C, with a relatively larger proportion of the total residence time maintained at this temperature.

4) In the whole work, only one homopolymer and one block copolymer were used. It was well-known that the crystallization rate/kinetics was highly dependent on the composition of crystalline polymers/copolymers. Were there any strict requirements for the chain length and composition of homopolymer/copolymer? If it is, some comments on this points should be provided.

Response: We thank the reviewer for raising this important point. In our previous work, we employed a well-defined PCL_{50} homopolymer and PCL_{50}-*b*-$PDMA_{200}$ diblock copolymer as model systems for platelet formation. However, these two polymers exhibited markedly different crystallization kinetics and did not display thermally responsive behavior during living growth. Based on this, our design strategy was to use PCL_{50} as a benchmark and systematically adjust the composition of the diblock copolymer to achieve a crystallization capacity comparable to that of the homopolymer. This matching of crystallization kinetics is critical, as it enables external stimuli (e.g., temperature) to effectively modulate the relative crystallization rates of the two components.

More generally, while other combinations of homopolymers and block copolymers could in principle be employed—as demonstrated in our earlier studies (Macromolecules 2024, 57, 23, 11210–11220.)—there are additional constraints beyond crystallization compatibility. In particular, the overall kinetics of living growth must be compatible with the flow setup. Systems with excessively fast crystallization kinetics hinder precise temporal control of temperature changes within short residence times, whereas very slow kinetics would require impractically long tubing to achieve sufficient aging times. Therefore, both chain length and composition must be carefully selected to balance crystallization behavior with processability under flow conditions.

To clarify this rationale, we have added the following explanation on **Page 6, Line 9-12** in the revised manuscript.

In contrast to our previous work,[12, 14, 18, 26, 29] a diblock copolymer (PCL_{136}-*b*-$PDMA_{200}$) with a much longer PCL block was prepared to increase the crystallization capacity to a level comparable to that of the PCL_{50} homopolymer, thereby enabling external stimuli to modulate their relative crystallization rates.

5) Just for curiosity, what would happen for this system at lower or higher temperatures? Most readers might ask the same question. It will be much better if additional results could be provided on this point.

Response: We thank the reviewer for this insightful comment. Temperature indeed plays a critical role in governing the crystallization behavior and structural integrity of the platelets in this system.

At higher temperatures, the system approaches the melting point of PCL in ethanol (~36 °C as determined by nano DSC), which introduces a significant risk of partial or complete melting of both the seed crystallites and the already-formed platelets. This can disrupt the living growth process and compromise the structural stability of the assemblies. In contrast, at lower temperatures, although increased supercooling promotes crystallization, the reduced chain mobility can lead to imperfect chain packing and the formation of crystallinity defects within the platelets. Upon returning to room temperature, the resulting temperature increase may further drive chain rearrangement, potentially disrupting the platelet structure and leading to fragmented platelets, as observed in previous work (Nat. Synth 3, 903–912 (2024).). Therefore, an intermediate temperature window is required to balance sufficient chain mobility with stable crystallization.

6) In figure 1, it appeared that the temperature can affect the aspect ratio of resulting micelles. Interestingly, in figure 3, it appeared that the aspect ratio of resulting micelles almost did not change obtained at different heating-cooling cycles. It will be better if some comments on this point could be provided.

Response: We thank the reviewer for raising this point. Indeed, temperature plays an important role in determining the aspect ratio of the resulting platelets by modulating the overall growth kinetics. Although the homopolymer and diblock copolymer exhibit different temperature-dependent growth behaviors, the overall growth kinetics of the mixed system are primarily dominated by the homopolymer component. As a result, when mixtures of homopolymer and diblock copolymer are used as the unimer feed, the aspect ratio shows a temperature dependence similar to that of the homopolymer. PCL crystallizes along the (110) and (200) planes, and elevated temperatures reduce the overall crystallization rate, favoring growth along the (110) plane, which has lower surface energy (Macromolecules 2024, 57, 23, 11210-11220). This preferential growth leads to platelets with higher aspect ratios. In contrast, under cooling conditions, the

crystallization rate increases significantly, providing sufficient driving force to overcome the energy barriers associated with both crystallographic planes more uniformly, resulting in platelets with lower aspect ratios.

For the samples shown in Figure 3, we do observe gradual changes in aspect ratio with increasing heating-cooling cycles, as presented in Figure S27. Specifically, as the growth is initiated under heating conditions, the first layer exhibits a relatively high aspect ratio (3.21). With additional heating-cooling cycles, a larger fraction of the platelet area forms under cooling conditions, leading to a gradual decrease in the overall aspect ratio.

To clarify this point, we added the following sentence on **Page 10, Line 6-13** in the revised manuscript to comment on the results in Figure 1.

Differences in crystallization kinetics at varying temperatures play a critical role in determining the aspect ratio of the resulting platelets (Figure 1d and Table S2). PCL crystallizes along the (110) and (200) planes, and at elevated temperatures the overall crystallization rate is reduced, favoring growth along the (110) plane, which possesses lower surface energy.[38] This preferential growth leads to platelets with higher aspect ratios at 30 °C. In contrast, under cooling conditions (e.g., 10 °C), the overall crystallization rate increases significantly, providing sufficient driving force to overcome the energy barriers associated with both crystallographic planes more uniformly, resulting in platelets with lower aspect ratios.

The following sentences were added on **Page 17, Line 17-25** in the revised manuscript to comment on the results in Figure 3.

Additionally, directional growth rates offer direct morphological insight into the anisotropic growth behavior of the platelets. Notably, the aspect ratio of the platelets decreased with increasing number of heating–cooling cycles (Figure S27). As growth is initiated under heating conditions, the first layer exhibits a relatively high aspect ratio (3.21). With additional heating–cooling cycles, a larger fraction of the platelet area is formed under cooling conditions, resulting in a gradual reduction in the overall aspect ratio. This trend can be mathematically predicted that under conditions of sufficient unimer supply, increasing heating cooling cycles would drive the aspect ratio toward a limiting value of 1.79 (approximate to the theoretical value of 1.68, the ratio of growth rates along the long and short axes).

Reviewer #3

This highly intriguing paper deals with the preparation of well-defined platelets with a tree-ring-like surface pattern employing co-crystallization-driven self-assembly of poly(ε-caprolactone)-block-poly(N,N-dimethylacrylamide) (PCL-b-PDMA) diblock copolymers with PCL homopolymers. The use of precisely programmed flow reactors allows continuous production of the platelets and an efficient tuning of corona structure by variations in temperature and residence time, whereby temperature is the decisive parameter to control co-assembly over self-sorting due to different crystallization kinetics of the diblock copolymer and homopolymer. This enables a precise encoding of self-assembly history into the corona structure of the platelets, resembling the encoding of growth history (in terms of environmental conditions) observed for trees. Moreover, growth kinetics can be directly extracted from the encoded structures, which is difficult (or impossible) to achieve by "conventional" CDSA methods. The approach presented is highly appealing and there are currently no alternative methods available to achieve tree-ring-like structures with such high precision. Hence, publication in Nat. Commun. is strongly supported after minor revisions as detailed below.

I) Rate constants derived from UV-vis

The authors describe a kinetic model that allows determination of growth constants from UV-vis measurements. The model is sound; however, the evaluation of UV-vis data raises some questions. First, the fits presented in Figure S13 do not always properly describe the experimental data. Especially for the diblock copolymer at 30 °C there is a significant deviation. In addition, the standard deviations for variables a, b, and c are very high, even exceeding the mean in most cases (most pronounced for homopolymer kinetics). The chosen fitting model does also deviate from the kinetic model. For fitting A(t) = a(1-exp(-b*t^c)) is used, whereby the kinetics model is A(t) = K*(1-exp(-k'*t)) (k' = k [S]0). According to the model, the extracted apparent rate constant should have the unit [L/(mol*min)] as seed concentration is included in k'; one page 9 1/min is used as unit instead. Hence, it is recommended to discuss the data only qualitatively without giving rate constants. For example, plotting the kinetics for diblock copolymer and homopolymer in one plot for a given temperature would also show that homopolymer crystallization is faster at low temperatures and comparable at high temperatures. Is it possible to use the Nano-DSC to study crystallization kinetics?*

Response: We thank the reviewer for this careful and insightful comment. We initially aimed to quantitatively compare the growth behavior of the different polymers using apparent rate constants. However, we acknowledge that the fitting model and extracted parameters were not fully consistent with the kinetic model, and that the reported units were incorrect. In the revised manuscript, we have therefore removed all rate constant analysis and instead focus on direct qualitative comparison of the growth kinetics.

Accordingly, in the revised manuscript we have removed the discussion of extracted rate constants and instead directly compared the growth kinetics of the homopolymer and diblock copolymer at different temperatures. These revised plots clearly show that the difference in growth rate between the two polymers is relatively small at 30 °C, whereas at 10 °C the homopolymer exhibits substantially faster growth kinetics, even though the diblock copolymer measurements were conducted at elevated concentration. In addition, we have included the growth kinetics of the polymer mixture at different temperatures to better represent the overall growth behavior under the experimental conditions used for self-assembly. The results demonstrate that the overall growth kinetics decrease with increasing temperature, while all systems still complete the growth process within minutes, despite the relatively slow kinetics observed for the diblock copolymer alone at 10 °C.

Regarding the reviewer's suggestion to use nano DSC, we agree that DSC techniques can provide valuable information on crystallization behavior. However, in our seeded growth system the kinetic monitoring begins immediately upon mixing the seed micelles with the unimer solution. Capturing this rapid mixing-initiated growth process in a nano DSC experiment is experimentally challenging, particularly with respect to achieving sufficiently fast and reproducible mixing prior to the onset of crystallization and growth.

This following paragraph has been updated on **Page 9 Line 17-27**, and **Page 10 Line 1** in the revised manuscript.

The growth kinetics of the two polymers were tested separately to evaluate their temperature dependence (Figures S13a-b). The homopolymer exhibited faster growth kinetics at lower temperatures due to increased supercooling, consistent with prior reports on crystallization in the bulk state.[24, 39] In contrast, the diblock copolymer showed the opposite trend, with lower temperatures resulting in slower growth kinetics. Consequently, the difference in growth kinetics between the two polymers was relatively small at 30 °C, but became substantially larger at 10 °C (Figures S13d-f), even though the diblock copolymer measurements at 10 °C were conducted at elevated concentration. These observations are consistent with

the experimentally observed co-assembly and self-sorting behavior at 30 °C and 10 °C, respectively. Nevertheless, when a mixture of homopolymer and diblock copolymer was used as the unimer, all systems investigated between 10 and 30 °C completed the growth process within minutes (Figure S13c).

Updated Figure S13 in the revised Supporting Information

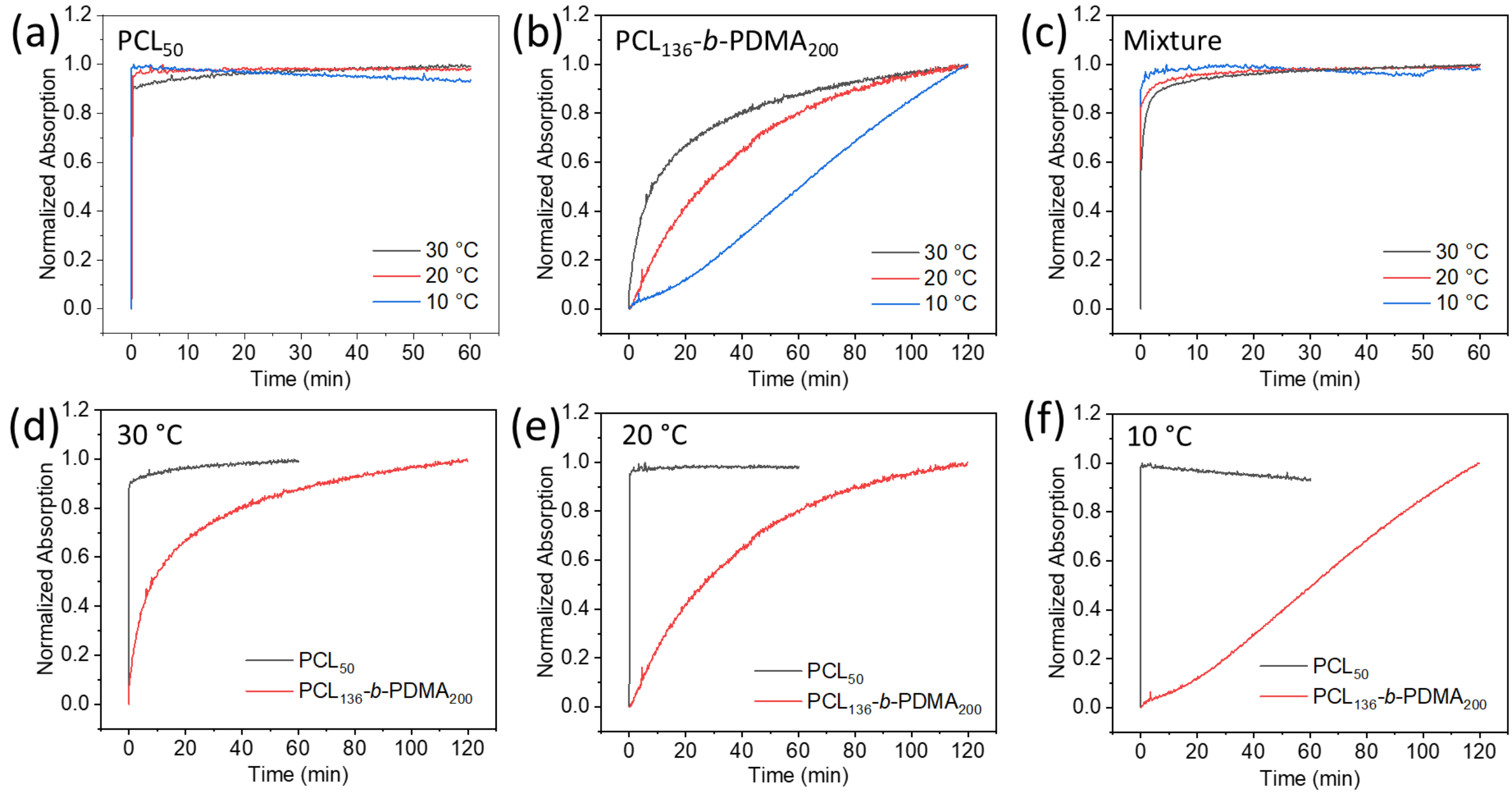

Figure S13. UV-Vis spectra used to monitor seeded growth kinetics by tracking signal intensity at 500 nm over time: (a)-(c) growth kinetics of PCL_{50}, PCL_{136}-*b*-$PDMA_{200}$, and a mixture of PCL_{50}/PCL_{136}-*b*-$PDMA_{200}$ (1:1 wt%), respectively, at various temperatures, and (d)-(f) comparison of the growth kinetics of PCL_{50} and PCL_{136}-*b*-$PDMA_{200}$ at 30, 20, and 10 °C respectively. All measurements were performed at a concentration of 0.1 mg mL^{-1}, except for PCL_{136}-*b*-$PDMA_{200}$ at 10 °C, which was conducted at 1 mg mL^{-1} due to the slow kinetics and weak UV–Vis signal changes observed at lower concentration.

II) Readability of Figures

In Figure 1d the axes labels are hard to read. It is recommended to rearrange the Figure.

In Figure 4d it is difficult to distinguish between pale orange and pale red color in flow schemes. It is recommended to either use a different color combination or reduce transparency. In addition, due to the

small size it is hard to see differences in the provided height profiles. The Figure might be rearranged to allow larger sized plots or some of the results could be moved to Supporting Information.

Response: We thank the reviewer for these helpful suggestions. In the revised manuscript, Figures 1d and 4d have been restructured to improve readability and overall clarity. Specifically, the color combination in the flow schemes has been adjusted to provide clearer visual distinction, and the transparency has been reduced accordingly. In addition, the height profile figures have been enlarged and highlighted to improve visibility of the differences between samples.

III) QCM data

On page 20 the authors state that the hydrophobic nature of PCL renders the surface of the PCL platelets less hydrophilic. This might be kind of misleading, as the surface of neat PCL platelets should be rather hydrophilic due to the presence of polar RAFT groups and OH-end groups at the fold surface. This is also supported by the rather small difference in frequency shift between platelets with and without corona. Nevertheless, the neat PCL platelets are less hydrophilic with respect to the layered platelets with hydrophilic PDMA domains. The measured differences between platelets with varying layer numbers are comparable to the standard deviations. Hence, it is difficult to draw a conclusion on the dependence on layer number and only the difference between neat PCL and layered platelets might be discussed. Measuring the interfacial tension might be a more sensitive method to probe the influence of corona pattern.

Response: We thank the reviewer for this insightful comment and agree that the presence of polar RAFT end groups and hydroxyl chain ends at the fold surface can contribute to increased surface hydrophilicity of neat PCL platelets. However, these functionalities are located at chain ends and therefore represent only a small fraction of the overall polymer composition. As a result, the interfacial properties are still largely governed by the hydrophobic PCL crystalline domains. Consistent with this, we observe a measurable difference in frequency shift between neat PCL platelets and layered platelets containing hydrophilic PDMA domains under identical QCM conditions.

To avoid any ambiguity, the corresponding discussion on **Page 21 Line 11-14** in the revised manuscript has been clarified accordingly, as shown below.

When the sensor was modified with platelets lacking coronas, surface exhibited relatively lower hydrophilicity compared to corona-containing platelets, as reflected by a reduced QCM frequency shift response under identical conditions.

We acknowledge that there are deviations among the measured results. To minimize experimental error, the measurements were repeated multiple times, and the reported values represent the averages with corresponding standard deviations. Although the differences between samples are relatively small, the consistent trends observed across repeated measurements suggest that the results remain qualitatively meaningful and reasonably reliable within the experimental uncertainty. We also appreciate the reviewer's suggestion regarding interfacial tension measurements. We attempted such measurements; however, the obtained data showed considerable variability and large experimental errors, preventing reliable interpretation at this stage. We therefore believe that a more systematic investigation using more sensitive and accurate characterization methods would be valuable to further elucidate the influence of corona patterns in future work.

IV) Minor comments on manuscript

Response: We thank the reviewer for the detailed and helpful suggestions. We have addressed each of them in our point-by-point response below.

- A recent report on the preparation of platelets with stripe-like surface patterns by co-crystallization-driven self-assembly might be added to the Introduction, which also allows to "write" defined corona patterns in a one-step procedure (Macromolecules 2026, 59, 3092).

The paper was cited as **Ref 17** in the Introduction of the revised manuscript, as shown below.

Importantly, varying the polymer composition and processing conditions impacts key structural features, such as morphology, stability, and size, allowing precise engineering of functional nanostructures.[17]

- The commonly used term "diblock polymer" might be replaced by "diblock copolymer"

We have replaced "diblock polymer" with "diblock copolymer" throughout the files of manuscript and Supporting Information to align with standard terminology.

- Polycaprolactone should be replaced by poly(ε-caprolactone).

We have replaced "polycaprolactone" with "poly(ε-caprolactone)" throughout the text.

- Page 6: it is recommended to add a short note that the used seeds were dispersed in ethanol at c = 0.01 mg/mL, otherwise the procedure might not be clear to the reader.

A note has been added to **Page 6 Line 19-20** of the revised manuscript, as shown below.

Following the addition of unimer (PCL_{50} / PCL_{136}-*b*-$PDMA_{200}$, 1:1 weight ratio, 10 mg mL^{-1} in $CHCl_3$; Figure 1a) to seed solution (0.01 mg mL^{-1} in ethanol), platelets with diverse morphologies were formed, as shown in the atomic force microscopy (AFM) images.

- Figures 2 - 4: please specify the diblock copolymer content in the unimer mixture, employed unimer-to-seed ratio, and flow rate for means of clarity. In addition, a note might be added on page 12 that details on employed flow rates and concentrations can be found in Supporting Information.

The following notes were added in the captions for Figure 2-4 respectively in the revised manuscript.

Figure 2. Discrete stage control of living CDSA via temperature switching in flow: (a) Schematic illustration of the flow setup with temperature-switching capability, (b) 3-Layer platelets prepared by sequential aging at 30 °C for 30 s, followed by 10 °C for 60 s, and (c) Tunable inner layer size achieved by varying the first-stage aging time at 30 °C (10, 30, and 60 s respectively), followed by aging at 10 °C. Scale bar = 2 μm (b) and 500 nm (c). Unimer (PCL_{50} / PCL_{136}-*b*-$PDMA_{200}$, 1:1 weight ratio, 10 mg mL^{-1} in $CHCl_3$) and seed solution (0.01 mg mL^{-1} in ethanol) were introduced at flow rates of 100 and 1 μL min^{-1}, respectively.

Figure 3. Cyclic temperature programming and growth kinetics in the formation of tree-ring-like multilayered platelets: (a) Schematic illustration of the flow setup with multistep temperature-switching capability, (b) AFM image of 13-layer platelets, shown as 3D rendering (left) and 2D height map (right), (c) Height profiles along the long (upper) and short (bottom) axis of platelets, and (d) Linear epitaxial growth of platelets. In the fitted equation, *D* represents the axial growth distance and *x* denotes the heating-

cooling cycle. Data was collected at the troughs of height profiles. Scale bar = 500 nm. Unimer (PCL_{50} / PCL_{136}-*b*-$PDMA_{200}$, 2:1 weight ratio, 5 mg mL^{-1} in $CHCl_3$) and seed solution (0.0025 mg mL^{-1} in ethanol) were introduced at flow rates of 200 and 2 μL min^{-1}, respectively. Further details of the flow conditions and concentrations are provided in the Supporting Information.

Figure 4. Encoding growth histories in platelets via programmable crystallization kinetics: (a) Schematic illustration of the flow setup used to fabricate multilayered platelets through controlled temperature and residence time programming, (b) AFM images of 7-layer platelets, shown as 3D rendering (left) and 2D height map (right), (c) Decoding growth histories from height profiles of multilayered platelets, and (d) Platelets prepared under various programmed temperature and residence time conditions. Top row: simplified flow schemes; middle row: 3D AFM images; bottom row: 2D AFM images with corresponding height profiles. Scale bar = 500 nm. Unimer (PCL_{50} / PCL_{136}-*b*-$PDMA_{200}$, 2:1 weight ratio, 5 mg mL^{-1} in $CHCl_3$) and seed solution (0.0025 mg mL^{-1} in ethanol) were introduced at flow rates of 100 and 1 μL min^{-1}, respectively.

- Can the authors comment on why for the growth kinetics (e.g. Fig. S22, S23) negative y-axis intercepts were found. In principle, one might expect that dimensions of seeds (ca. 25 nm) define the intercept.

We thank the reviewer for raising this point. We reason that the negative y-axis intercepts arise from the initiation efficiency of the seeds. Although all seeds are ultimately activated for growth, an initial self-regulation period is likely required before unimers can efficiently access the growing fronts. In addition, delayed homogenization of the seed and unimer streams under laminar flow conditions may further contribute to this induction period. Together, these effects introduce a time delay in the onset of epitaxial growth, which may lead to the observed negative intercepts in the linear fitting. Therefore, the fitted intercepts do not directly correspond to the initial seed dimensions.

To indicate this point, the following sentences were added to **Page 16 Line 25-27** and **Page 17 Line 1-2** of the revised manuscript.

This variability may arise from differences in seed initiation efficiency, likely due to partial shielding of growth fronts by coronas, or from delayed homogenization of the seed and unimer streams under laminar flow conditions.[49, 50] Such delayed initiation may also account for the negative y-axis intercepts observed

in the linear fittings, as an induction period is required before unimers can efficiently access the active growth fronts.

V) Supporting information

Response: We appreciate the reviewer's careful reading and constructive feedback on the Supporting Information. A point-by-point response to each comment is provided below.

- Figure S6, S15, S19: height profiles are hard to read due to limited resolution and font size.

The height profiles in Figures S6, S15, and S19 have been regenerated in Origin with improved resolution and larger font sizes to provide better visibility and readability. The updated figures are now **Figures S6, S14, and S18** in the revised Supporting Information.

- Figure S5, S16: it is recommended to provide z-scales.

We thank the reviewer for this suggestion. Previous Figures S5 and S16 (now Figures S5 and S15) are low-magnification AFM images corresponding to the higher-magnification images shown in Figures 1d and S14, where the z-scales are already provided. The purpose of these figures is primarily to present the overall particle morphology and uniformity over a larger area. Adding z-scales, particularly in Figure S16, would reduce the available image area and make the particle features more difficult to observe clearly. Therefore, z-scales were not added to the low-magnification overview images, while all high-magnification AFM images used for height analysis include z-scale information.

- Table S6-S10: it is recommended to give only one digit for time and increment.

The values in these tables have now been revised to one decimal place for consistency and clarity. Please see the updated tables presented as **Table S11-S15** in the revised Supporting Information.

- Figure S22a-S25a: it is recommended to increase the size of the AFM images, as details are difficult to recognize.

These figures have been rearranged, and the AFM images have been enlarged to improve the visibility of structural details. Please see the updated figures presented as Figure S22-S25 in the revised Supporting Information.

- Figure 24c, 25c: here, growth time t is used for fitting the data; however, in all other related Figures the cycle number x is used. It is recommended to uniformly use the cycle number x for fitting to allow for comparison.

We thank the reviewer for this helpful suggestion. In the revised manuscript, equations fitted using the cycle number (x) have been added to Figures S24c and S25c. At the same time, the equations fitted using growth time (t) have also been retained, as different flow rates were employed in the main text to demonstrate comparable growth rates in terms of nm s^{-1}.

- Figure S27: the standard deviations given in the Table are very high, maybe there is a problem with units? Please replace "layer thickness" by "aspect ratio".

We thank the reviewer for raising this important point, which we had not previously noticed. The large uncertainties associated with the individual fitting parameters arise from strong parameter correlation within the rational fitting function. For example, multiplying all parameters (A, B, C, and D) by the same factor (e.g., 10) produces an identical fitting curve for the function $(Ax+B)/(Cx+D)$. As a result, multiple parameter combinations can generate nearly indistinguishable fitting results, leading to excellent overall fitting quality despite poorly constrained individual parameters. Therefore, the fitting was used primarily to describe the overall trend and limiting behavior rather than to extract physically meaningful individual fitting constants.

We have added the following sentences below **Figure S27** to clarify this point for readers.

The relatively large deviations associated with the individual fitting parameters arise from strong parameter correlation within the rational fitting function, where multiple parameter combinations can produce nearly identical fitting curves. Therefore, the fitting was primarily used to describe the overall trend and limiting behavior of the aspect ratio variation rather than to extract physically meaningful individual fitting parameters.

“Layer thickness” has been replaced by “aspect ratio” in the revised Supporting Information.

- Figure S28: height profiles might be included for better visualization of layer numbers.

Height profiles have been included in the revised figure. Please see the updated figure presented as Figure S30 in the revised Supporting Information.

- Please check SI for typos and correct wording. For example: last sentence on page S3 seems to be incomplete, polydimethylacetamide should read poly(N,N-dimethylacrylamide) on page S6, “normalized size exclusion chromatograms” should be replaced by “apparent molar mass distribution” on page S12 and S18, “for stain” might be replaced by “as staining agent” on page S14, please use uniform notation for diblock copolymer in Figure S4 and S5 (PCL_{50}-b-$PDMA_{196}$),…

We thank the reviewer for carefully checking the Supporting Information and for identifying these issues. The typos, wording, and notation inconsistencies mentioned above have all been corrected in the revised Supporting Information.
